# Identification of metabolically stable 5′-phosphate analogs that support single-stranded siRNA activity

**DOI:** 10.1093/nar/gkv162

**Published:** 2015-03-09

**Authors:** Thazha P. Prakash, Walt F. Lima, Heather M. Murray, Wenyu Li, Garth A. Kinberger, Alfred E. Chappell, Hans Gaus, Punit P. Seth, Balkrishen Bhat, Stanley T. Crooke, Eric E. Swayze

**Affiliations:** Isis Pharmaceuticals Inc., 2855 Gazelle Ct, Carlsbad, CA 92010, USA

## Abstract

The ss-siRNA activity *in vivo* requires a metabolically stable 5′-phosphate analog. In this report we used crystal structure of the 5′-phosphate binding pocket of Ago-2 bound with guide strand to design and synthesize ss-siRNAs containing various 5′-phosphate analogs. Our results indicate that the electronic and spatial orientation of the 5′-phosphate analog was critical for ss-siRNA activity. Chemically modified ss-siRNA targeting human apoC III mRNA demonstrated good potency for inhibiting ApoC III mRNA and protein in transgenic mice. Moreover, ApoC III ss-siRNAs were able to reduce the triglyceride and LDL cholesterol in transgenic mice demonstrating pharmacological effect of ss-siRNA. Our study provides guidance to develop surrogate phosphate analog for ss-siRNA and demonstrates that ss-siRNA provides an alternative strategy for therapeutic gene silencing.

## INTRODUCTION

The demonstration that the reduction of mRNA expression in cells using externally delivered duplex RNA (siRNA) by activating the RISC mechanism enabled the development of siRNA therapeutic (1,2). This approach has a potential to treat a wide variety of human diseases through genetic modulation (3). However, broader utility of siRNA is limited due to the requirement for complex lipid formulations or conjugation strategy to deliver siRNA to peripheral tissues (4–6). In contrast, single-strand antisense oligonucleotides do not require special formulations to distribute broadly to peripheral tissues (7). Moreover, it was shown that human Dicer and Ago-2, the enzymes involved in the RNAi pathway, bind short single-stranded RNAs with affinities comparable to siRNAs suggesting that single-stranded RNAs are capable of activating the RNAi pathway (8). Consistent with these observations we recently demonstrated single stranded short interfering RNA (ss-siRNA) activity in mice (9–11).

The critical determinants for ss-siRNA activity *in vivo* were a configuration resistant to exonuclease and endonuclease degradation and a metabolically stable 5′-phosphate (9). The 5′-phosphate was required for ss-siRNA but not for double-stranded siRNA activity (9). The 5′-phosphate is important for Ago-2 cleavage activity and binding (12–15). In this study we utilized the crystal structure of the 5′-phosphate binding pocket of Ago-2 protein to design chemical modifications to improve the metabolic stability of 5′-phosphate while maintaining the activity of ss-siRNA (12–15). This rational approach establishes stereoelectronics for 5′-phosphate of ss-siRNA to elicit RNAi. Finally, we show that a modified ss-siRNA targeting human ApoC III mRNA potently reduced target mRNA level *in vivo* resulting in reducing plasma triglyceride levels in a transgenic mouse model.

## MATERIALS AND METHODS

### General method for the preparation of ss-siRNAs containing 5′-deoxy-5′-methylenephosphonate and 5′-deoxy-5′-vinylphosphonate using solid phase synthesis

Unless otherwise stated, all reagents and solutions used for oligonucleotide synthesis were purchased from commercial sources. The standard phosphoramidites and solid supports were used for incorporation of A, U, G and C residues. A 0.1-M solution of 2′-F, 2′-*O*-Me, 2′-*O*-MOE, 5′-modified 2′-*O*-MOE phosphoramidites **9**, **11**, **30b**-**32b**, **36** and 2′-C16 U phosphoramidite **72** in anhydrous acetonitrile (CH_3_CN) were used for the synthesis. Phosphoramidites containing 5′-methylenephosphonate and its analogs **39**, **43b**, **46–47**, **49**, **53**, **55–57** and **71** were dissolved in 30% dichloromethane (CH_2_Cl_2_) in anhydrous CH_3_CN (0.1 M) and used for the solid phase synthesis. The modified oligonucleotides were synthesized on VIMAD UnyLinker^TM^ solid support and the appropriate amounts of solid supports were packed in the column for synthesis. Dichloroacetic acid (6%) in toluene was used as detritylating reagent. 4,5-dicyanoimidazole in the presence of *N*-methylimidazole or 1*H*-tetrazole in CH_3_CN was used as activator during the coupling step. The synthesis of modified oligonucleotides was performed either on an ÄKTAOligopilot synthesizer (GE Healthcare Bioscience) or an ABI 394 synthesizer on a 2–200-μmol scale using the procedures set forth below.

A solid support preloaded with the Unylinker^TM^ was loaded into a synthesis column after closing the column bottom outlet and acetonitrile (CH_3_CN) was added to form slurry. The swelled support-bound Unylinker^TM^ was treated with a detritylating reagent containing 6% dichloroacetic acid in toluene to provide the free hydroxyl groups. During the coupling step, four to fourteen equivalents of phosphoramidite solutions were delivered and the coupling was allowed to carry out for 10 min. All other steps in the protocol supplied by the manufacturer were used without modification. Phosphorothioate linkages were introduced by sulfurization with a 0.05-M solution of DDTT (3-((dimethylamino-methylidene)amino)-3H-1,2,4-dithiazole-3-thione) in 1:1 pyridine/CH_3_CN for a contact time of 3 min. Phosphate diester linkages were incorporated via oxidation of phosphite triesters using a solution of *tert*-butyl hydroperoxide/CH_3_CN/water (10:87:3) for a contact time of 12 min. After the desired sequence was assembled, the solid-support bound oligonucleotide was washed with CH_2_Cl_2_ and dried under high vacuum. After 4 h, the dried solid support was suspended in a solution of iodotrimethylsilane (TMSI) and pyridine in CH_2_Cl_2_ to remove the 5′-phosphonate protecting group (ethyl or methyl group). The deprotection solution was prepared by dissolving 0.75-ml TMSI and 0.53-ml pyridine in 28.2-ml CH_2_Cl_2_ (used 0.5 ml/μmol of solid support). After 30 min at room temperature, the reaction was quenched with 1-M 2-mercaptoethanol in 1:1 TEA/CH_3_CN (used 0.5 ml/μmol of solid support). The supernatant was decanted and the solid support was washed with the same 2-mercaptoethanol solution mixture from above. This step was repeated one more time, except after 45 min at room temperature, the supernatant was decanted and the solid-support-bound oligonucleotide was suspended in ammonia (28–30 wt%) : 1-M 2-mercaptoethanol (used 0.75 ml/μmol of solid support) and heated at 55°C for 2 h to complete cleavage from support. The cleaved solution was allowed to cool to ambient temperature over 24 h. The unbound oligonucleotide was then filtered and the support was rinsed and filtered with water:ethanol (1:1) followed by water. The combined filtrate and washing were neutralized using acetic acid, cooled at −20°C for 3–4 h. The ss-siRNAs were precipitated and it was collected by centrifugation and decanting the supernatant. It was red-dissolved in water and purified by HPCL on a reverse phase column (Waters X-Bridge C-18 5 μm, 19 × 250 mm, A = 5-mM tributylammonium acetate in 5% aqueous CH_3_CN, B = CH_3_CN, 0 to 90% B in 80 min, flow 7 ml min^−1^, λ = 260 nm). Fractions containing full-length oligonucleotides were pooled together (assessed by High Pressure Liquid Chromatography-Mass Spectrometry (HPLC-MS) analysis >95%) and the tributylammonium counter ion was exchanged to sodium by HPLC on a strong anion exchange column (GE Healthcare Bioscience, Source 30Q, 30 μm, 2.54 × 8 cm, A = 100-mM ammonium acetate in 30% aqueous CH_3_CN, B = 1.5 M NaBr in A, 0–40% of B in 60 min, flow 14 ml min^−1^). The residue was desalted by HPLC on reverse phase column to yield the ss-siRNAs in an isolated yield of 15–20% based on solid-support loading. The oligonucleotides were characterized by ion-pair-HPLC-MS analysis with Agilent 1100 MSD system.

### *In vitro* potency of ss-siRNA and siRNA in transfected HeLa cells

HeLa cells were seeded in 96-well plates at 5000–10 000 cells/well 16 h prior to treatment with the exception of liver hepatocytes which were immediately plated and transfected 2 h post perfusion. Transfection was performed at indicated concentrations using Opti-MEM medium (Life Technologies) containing 4–6-μg/ml Lipofectamine 2000 (Life Technologies) for 4 h at 37°C. Growth medium, Dulbecco's modified Eagle's medium for HeLa and Mouse Fibroblast (MEF) cell lines and Williams E for hepatocytes, was replaced and cells were incubated overnight at 37°C in 5% CO_2_. Cells were lysed 16 h post transfection and total RNA was purified using RNeasy 3000 Bio Robot (Qiagen). Reduction of target mRNA was determined by quantitative Reverse Transcriptase Polymerase Chain Reaction (qRT-PCR) as previously described (16). The primer-probe sequences used for detection of human PTEN were forward AATGGCTAAGTGAAGATGACAATCAT, reverse TGCACATATCATTACACCAGTTCGT and probe TTGCAGCAATTCACTGTAAAGCTGGAAAGG. Target mRNA levels were normalized to total RNA using RiboGreen (Life Technologies). IC_50_ curves and values were generated using Prism 4 software (GraphPad Prism regression analysis Software).

### *In vivo* activity of ss-siRNA in mice

Animal experiments were conducted according to American Association for the Accreditation of Laboratory Animal Care guidelines and were approved by the Animal Welfare Committee (Cold Spring Harbor Laboratory's Institutional Animal Care and Use Committee guidelines). Male Balb/c mice (Charles River Laboratories), aged 6–8 weeks, were maintained at a constant temperature of 23°C and were allowed to standard lab diet and water. Dosing solutions were prepared in phosphate-buffered saline, sterile filtered and quantified. Mice were dosed by single administration (*n* = 4), intravenous or subcutaneous, injection with the exception of subcutaneous doses above 50 mg/kg which consisted of subdivided injections of 25 mg/kg twice a day for indicated number of days. Mice were sacrificed 48 h post treatment. Animals were anesthetized with isoflurane and terminal bleed was performed as previously described (17). Immediately following terminal blood draw, mice were sacrificed by cervical dislocation while under anesthesia. Liver, kidney and spleen weights were taken and liver tissue was homogenized in guanidine isothiocyanate (Life Technologies) containing 8% β-mercaptoethanol (Sigma) immediately following the sacrifice. Liver homogenate was loaded onto Purelink PCR columns (Life Technologies) and total RNA was purified according to manufacture instructions. Reduction of target mRNA expression was determined by qRT-PCR as previously described (16). Target mRNA levels were normalized to cyclophilin levels and values were confirmed by RiboGreen. RNA purification and qRT-PCR was run as described above using the following hu apoc iii primer-probe sequences: forward GCCGTGGCTGCCTGAG, reverse AGGAGCTCGCAGGATGGAT and probe CCTCAATACCCCAAGTCCACCTGCC (18).

### Determination of tissue concentrations and metabolites of ssRNAs using LC-MS

Tissues were minced and 50–200-mg samples were homogenized in 500-μl homogenization buffer (0.5% NP40 substitute (Calbiochem) in Tris-buffered saline, pH8) with homogenization beads (Mo Bio Laboratories, Carlsbad, CA, USA) on a Retsch shaker (Mo Bio). Standard curves of each ss-siRNA were established in 500-μl aliquots control tissue homogenate (50–200-mg/ml homogenization buffer). A 27-mer, fully PS, MOE/DNA oligonucleotide was added as an internal standard (Int. Std.) to all standard curves and study samples. Samples and curves were extracted with phenol/chloroform followed by solid-phase extraction (SPE) of the resulting aqueous extract using phenyl-functionalized silica sorbent (Biotage, Upsalla, Sweden). Eluate from SPE was dried down using a warm forced-air (argon) evaporator and reconstituted in 100–200-μl 4-M urea, 25-mM ethylenediaminetetraacetic acid. Samples were analyzed by LC-MS using a modification of a previously described method (17). Brieﬂy, separation was accomplished using an 1100 HPLCMS system (Agilent Technologies, Wilmington, DE, USA) consisting of a quaternary pump, UV detector, a column oven, an autosampler and a single quadrupole mass spectrometer. Samples were injected on an X-bridge OST C18 column (2.1 x 50 mm, 2.5-μm particles; Waters, Milford, MA, USA) equipped with a SecurityGuard C18 guard column (Phenomenex, Torrance, CA, USA). The columns were maintained at 55°C. Tributylammonium acetate buffer (5 mM) and acetonitrile were used as the mobile phase at a flow rate of 0.3 ml/min. Acetonitrile was increased (gradient) from 20 to 70% over 11 min. Mass measurements were made online using a single quadrupole mass spectrometer scanning 1000–2100 *m/z* in the negative ionization mode. Molecular masses were determined using ChemStation analysis package (Agilent, Santa Clara, CA, USA). Manual evaluation was performed by comparing a table of calculated *m/z* values corresponding to potential metabolites with the peaks present in a given spectrum. Peak areas from extracted ion chromatograms were determined for ss-siRNAs, 3′ N-1 metabolites, and Int. Std. and a trendline established using the calibration standards, plotting concentration of ssRNA against the ratio of the peak areas ssRNA:Int. Std. Concentration of ssRNAs and 3′ N-1 metabolites in study samples were determined using established trendlines and reported as μg/g tissue.

## RESULTS

### 5′-phosphate is a critical determinant for ss-siRNA activity *in vivo*

**Figure 1.**
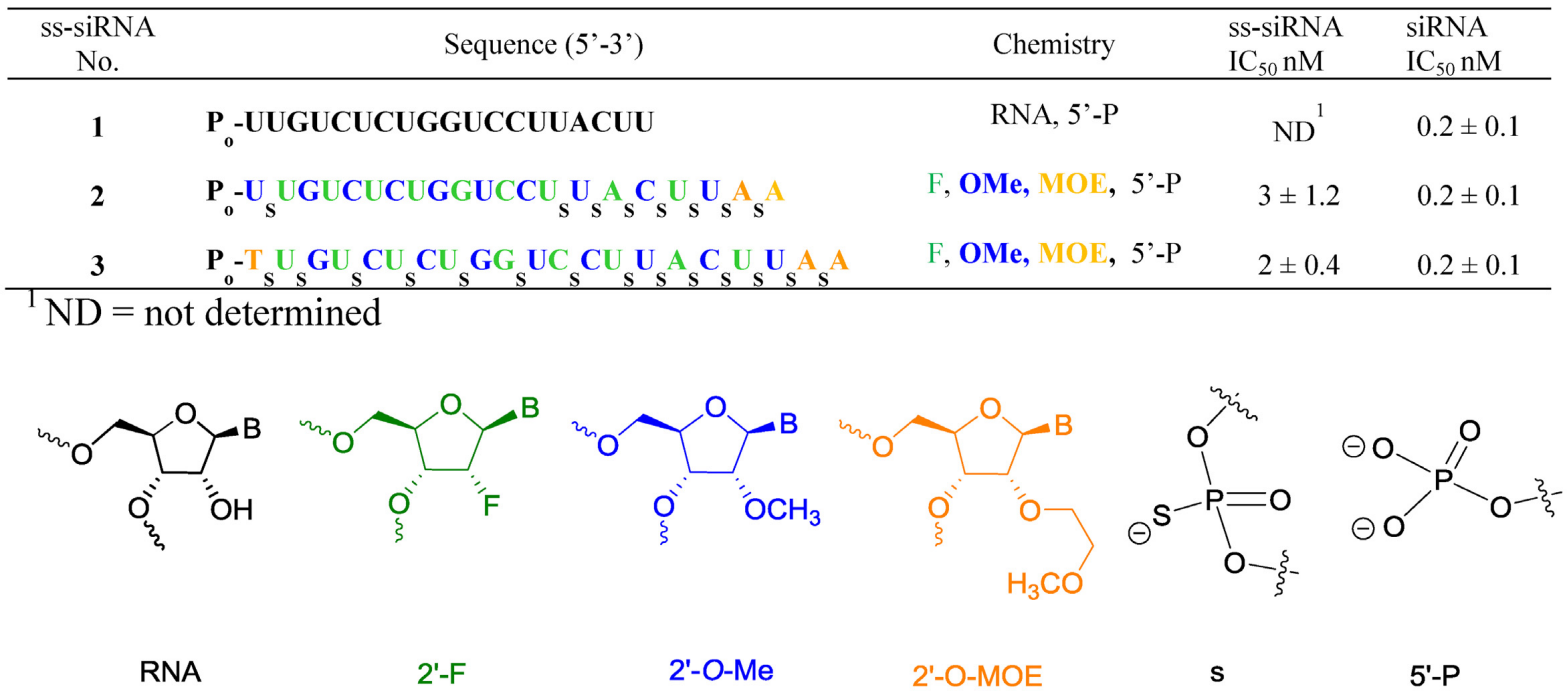
*In vitro* activity of chemically modified PTEN ss-siRNA and siRNA.

Our earlier structure-activity relationship (SAR) efforts identified chemically modified ss-siRNAs which exhibited excellent activity and metabolic stability in cell culture experiments (Figure 1) (9). However, these ss-siRNAs (Figure 1, **2–3**) showed no reduction in the Phosphatase and tensin homolog (PTEN) mRNA levels when evaluated in mice (9). Analysis of the livers of mice treated with ss-siRNAs **2–3** showed no intact ss-siRNAs. The predominant metabolite observed for ss-siRNA **2** consisted of the 3′-pole of the ss-siRNA containing the nine contiguous phosphorothioates. The predominant metabolite identified for ss-siRNA 3 consisted of full length compound without 5′-phosphate (9). The lack of *in vivo* activity observed for the ss-siRNA **3** was consistent with our observations that the 5′-phosphate is required for ss-siRNA activity (9).

### Approaches to study the effect of altering sterioelectronics of 5′-phosphate for ss-siRNA to activate RNAi

**Figure 2.**
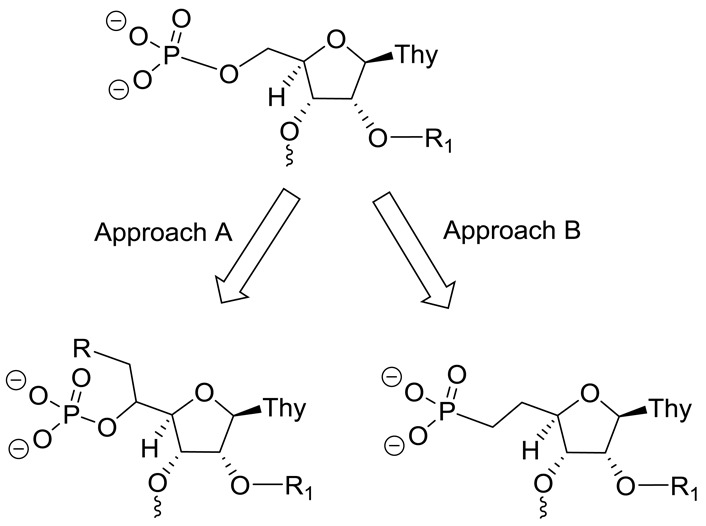
Chemical approaches to improve metabolic stability of 5′-phosphate of ss-siRNA.

Our *in vitro* and mechanistic studies showed that ss-siRNA activity requires a phosphate at the 5′-terminus (9). Given that the modified ss-siRNA **3** extracted from liver was rapidly dephosphorylated even after 6 h (9), we concluded that the natural phosphate was not metabolically stable for ss-siRNA to activate RNAi in animals (9,19). This prompted us to search for 5′-phosphate analogs that are stable in animals. We investigated two approaches to identify metabolically stable phosphate analogs for ss-siRNA. In the first approach, we evaluated the effect of substitution at the 5′-position (Figure 2, Approach A) on improving metabolic stability of 5′-phosphate. In an alternate approach, we replaced the bridging oxygen of the phosphate with carbon (Figure 2, Approach B) to provide 5′-methylenephosphonate which is stable to phosphatase-mediated cleavage.

We used our lead PTEN ss-siRNA **3** (Figure 1) for our phosphate structure activity study. The approach we utilized for this study comprises replacing 2′-*O*-(2-methoxyethyl)thymidine-5′-phosphate at the 5′-end of the ss-siRNA **3** with nucleotides containing 5′-modified phosphate analogs and evaluated *in vitro* potency in cell culture. We also compared the activity of ss-siRNA with siRNA generated by paring our ss-siRNAs with a complementary unmodified RNA. The ss-siRNAs with activities comparable to the control ss-siRNA **3** were then evaluated in animals to assess their metabolic stability. Animals were sacrificed at different time intervals and metabolites were determined using LC-MS analysis.

### Approach A: modification at the 5′-position of the 5′-terminal nucleotide of ss-siRNA

**Figure 3.**
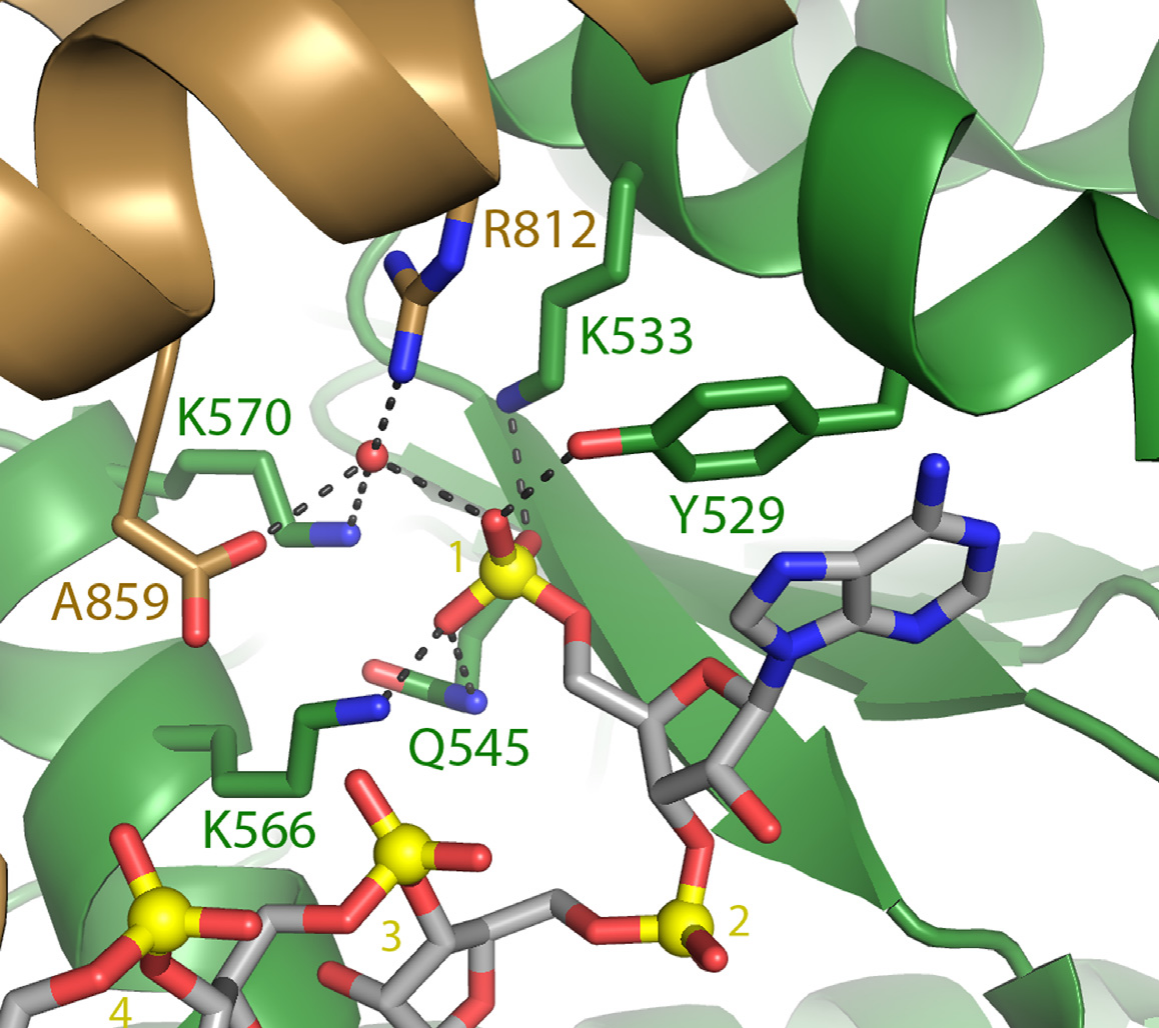
Conformation of 5′-phosphate of a guide strand bound to MID and PIWI domain of human Ago2; R: argine, K: lysine, Y: tyrosine, A: alanine, N: asparagine (12).

It has been reported that introduction of a methyl group (1:1 mixture of *R*- and *S*-isomers) at the 5′-position of the furanose ring in DNA greatly increases stability toward nuclease degradation (20). It was apparent from the crystal structure that the guide strand of siRNA bound to MID domain of human Ago-2 (Figure 3) (12), a methyl group at the 5′-position, would be tolerated.

**Figure 4.**
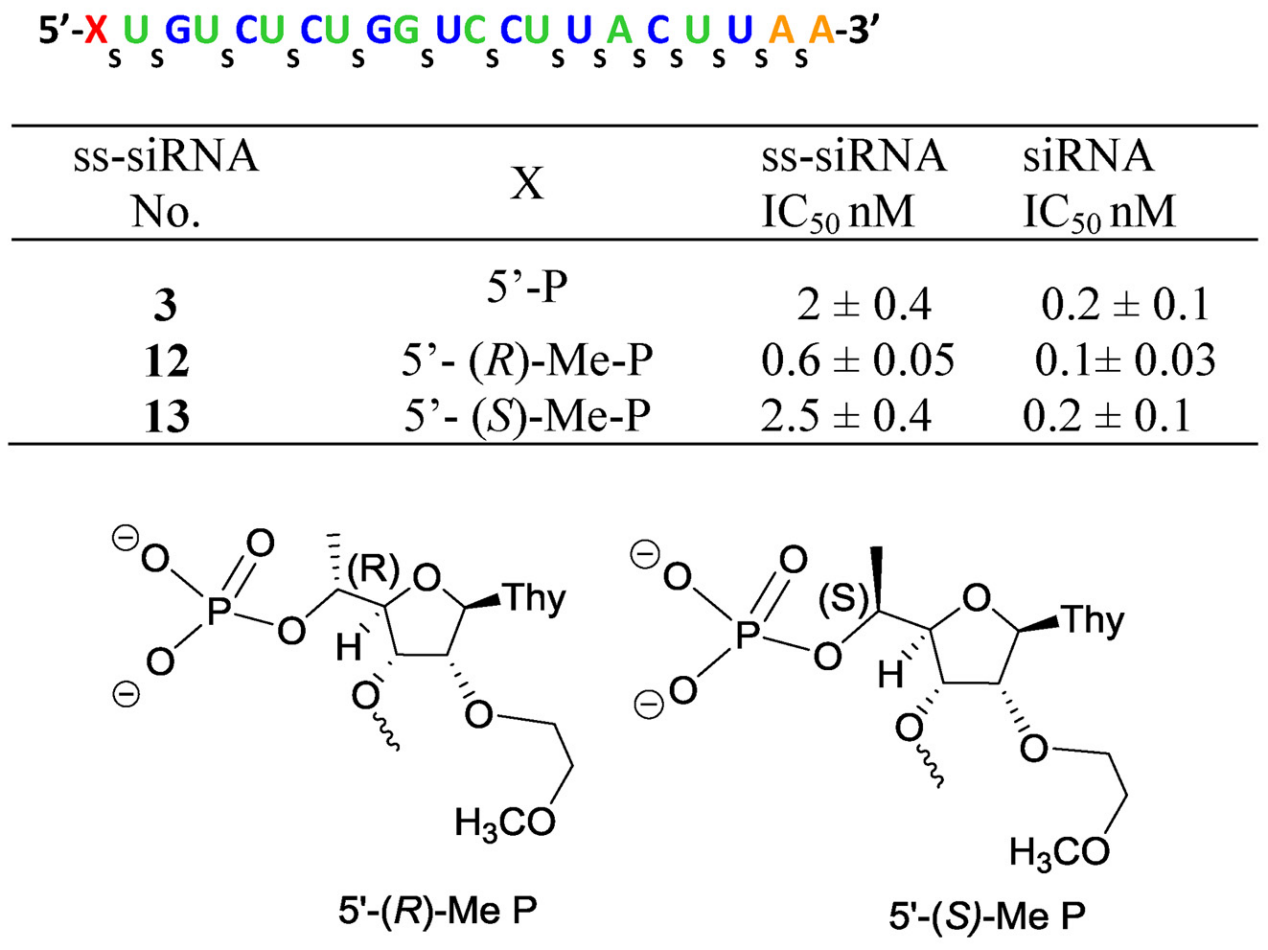
*In vitro* activity of 5′-modified PTEN ss-siRNA and siRNA in HeLa cells.

**Scheme 1.**
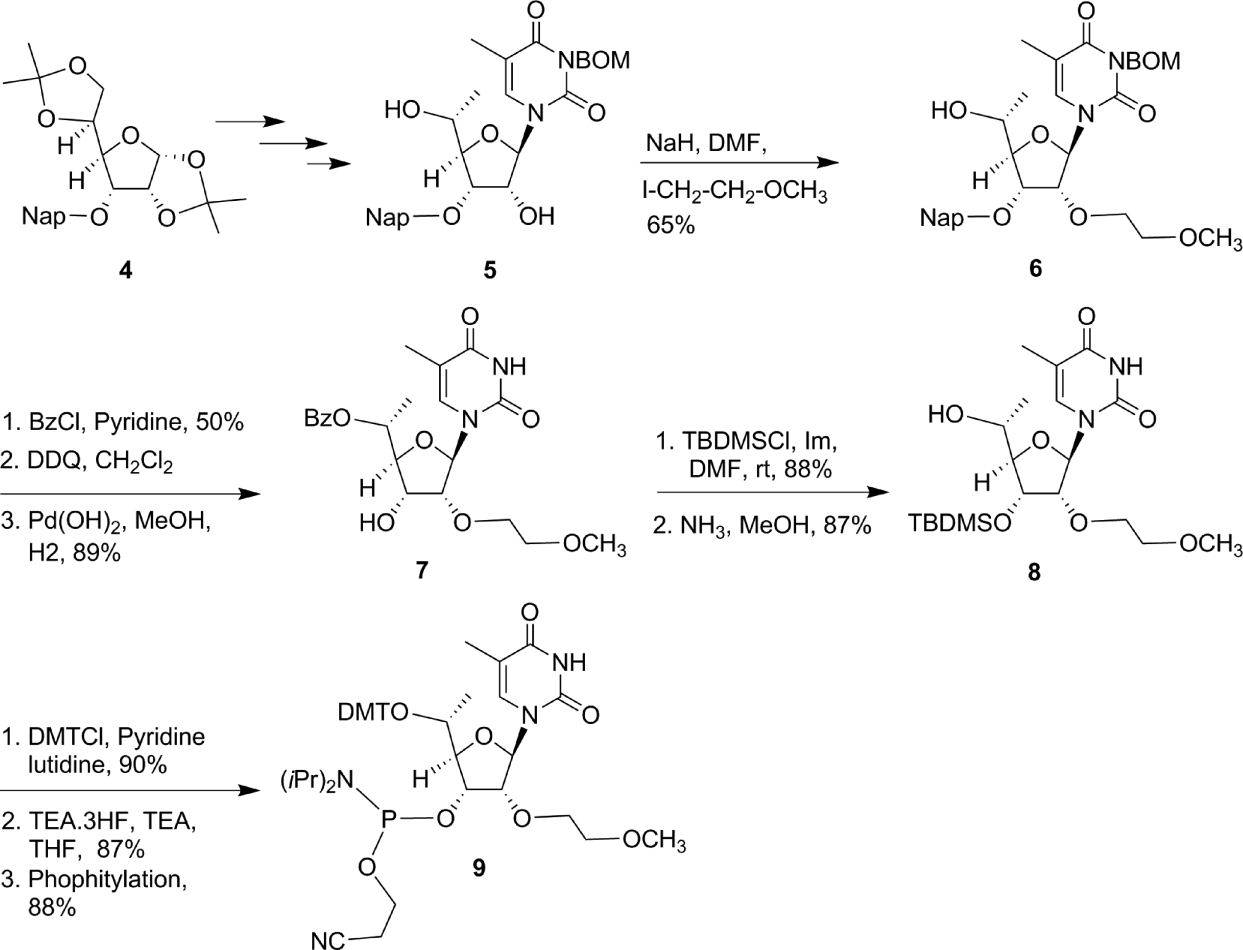
Synthesis of 5′-*O*-(4′,4′-dimethoxytrityl-(*R*)-5′-methyl-2′-*O*-(2-methoxyethyl)-thymidine-3′-phosphoramidite (**9**); Nap: 2-(methyl)naphthalene; BOM: benzyloxymethyl; Bz: benzoyl; DMT: 4,4′-dimethoxytrityl; TBDMS: *tert*-butyldimethylsilyl.

**Scheme 2.**
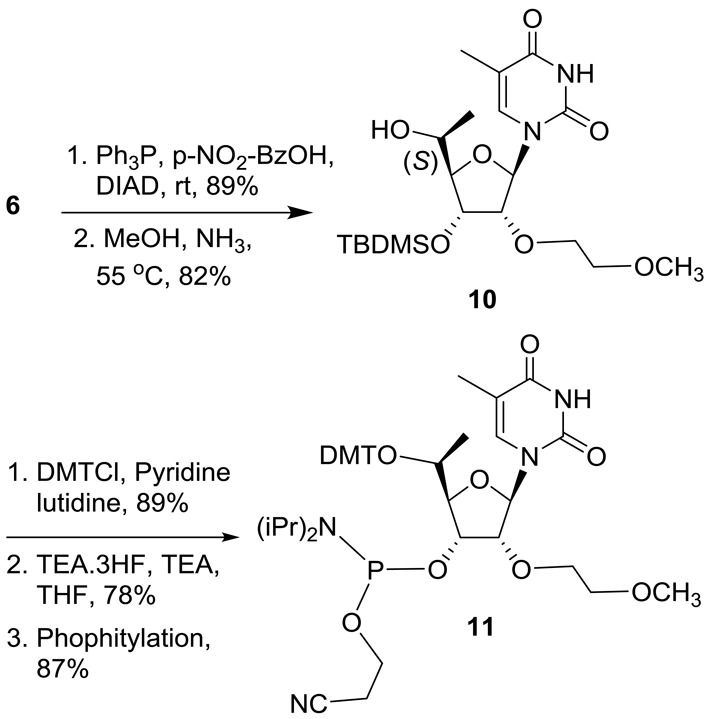
Synthesis of 5′-*O*-(4′,4′-dimethoxytrityl)- (*S*)-5′-methyl-2′-*O*-(2-methoxyethyl)-thymidine-3′-phosphoramidite (**11**).

In order to investigate this hypothesis we designed and synthesized (*R*)-5′-methyl-5′-*O*-(4,4′-dimethoxytrityl)-(2′-*O*-(2-methoxyethyl)thymidine-3′-phosphoramidite **9** (Scheme 1; Supplementary data) and (*S*)-5′-methyl-5′-*O*-(4,4′-dimethoxytrityl)-2′-*O*-(2-methoxyethyl) thymidine 3′-phosphoramidites **11** (Scheme 2; Supplementary data). ss-siRNA containing the (*R*)-5′-methyl **12** (Figure 4) and (*S*)-5′-methyl **13** (Figure 4) modifications at position 1 was synthesized. ss-siRNAs **12–13** were transfected to HeLa cells using Lipofectamine 2000. Reduction of target mRNA was determined by qRT-PCR as previously described (9). Target mRNA levels were normalized to total RNA using RiboGreen. The ss-siRNA **12** (IC_50_ 0.6 nM; Figure 4) containing (*R*)-5′-methyl was 5-fold more potent than (*S*)-5′-methyl modified ss-siRNA **13**, (IC_50_ 0.6 nM; Figure 4) and 3-fold more potent than the parent siRNA **3** (IC_50_ = 2 nM; Figure 4). In contrast, the potency of siRNA containing (*R*)-5′-methyl and (*S*)-5′-methyl ss-siRNA as guide strand and a fully complementary RNA as passenger strand were similar (Figure 4).

**Figure 5.**
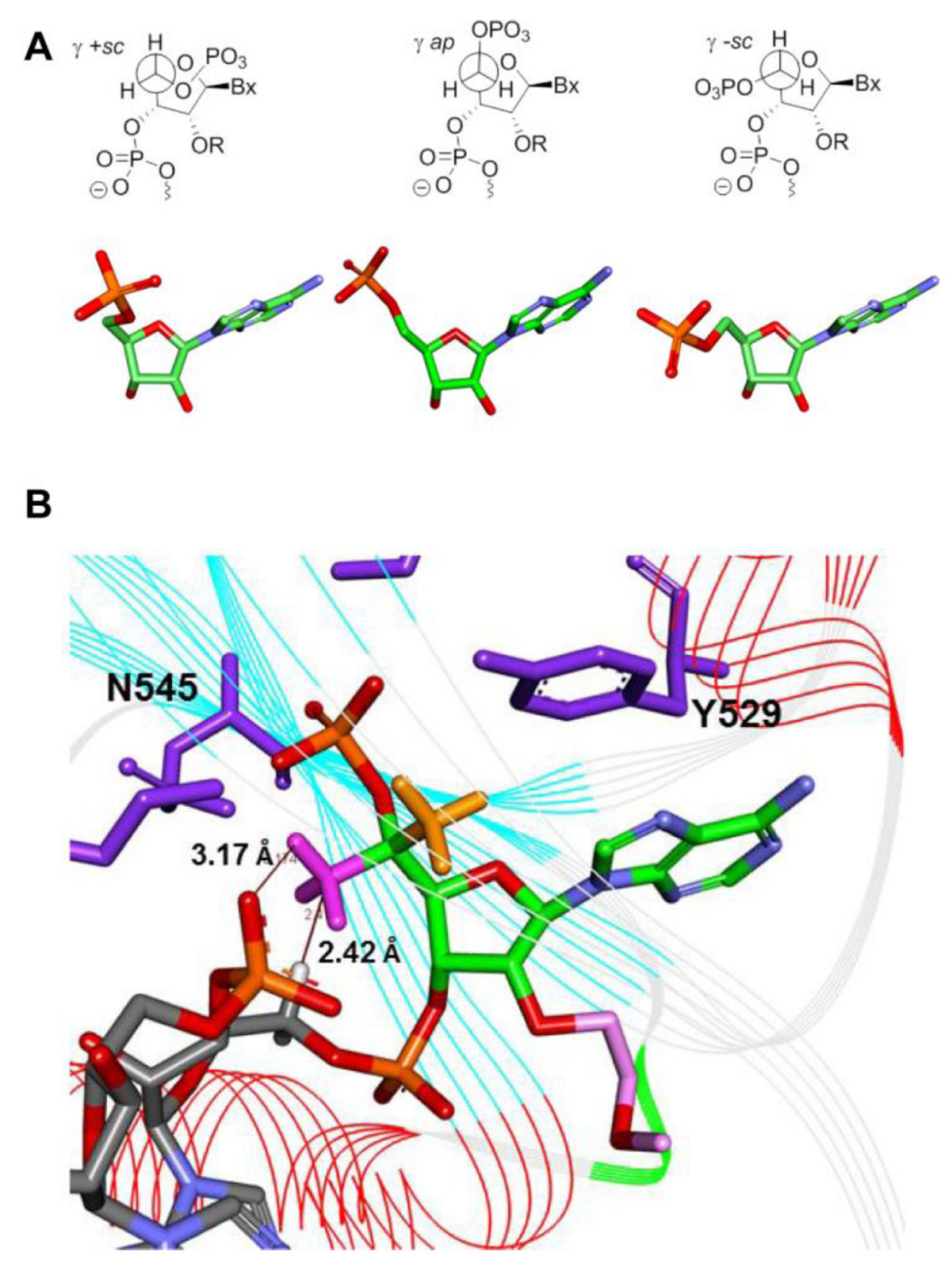
(**A**) Conformation of 5′-phosphate observed in guide strand of siRNA (12). (**B**) Overlaid structures of *R*-5′-methyl (golden) and *S*-5′-methyl (pink) structures and Ago-2 binding pocket (12).

Previous studies have shown that introducing an *R* configured 5′-Me group in locked nucleic acid neutralizes the high affinity recognition of complementary RNA by changing the torsional preference around γ from the *+sc* into the *ap* range (21). Interestingly, γ is also in the *ap* range for the 5′-phosphate in the Ago-2 crystal structure (Figure 5A) suggesting that the *R*-5′-Me group in **12** could pre-organize the conformational preference around γ to mimic this orientation and improve activity relative to the *S*-5′-Me analog **13**. Furthermore, a structural model of the *R*- and *S*-5′-Me groups in the Ago2 binding pocket shows that the bent trajectory of the neighboring phosphodiester linkage could cause the *S*-5′-methyl group to experience tight contacts with the 5′-methylene and one of the non-bridging oxygen atoms of the phosphodiester linkage of the adjacent nucleotide (Figure 5B). The absence of any stereochemical preference at the 5′-position of the guide strand of the duplex siRNA suggests a differential loading process for single- versus double-stranded nucleic acids into Ago-2 to form activated RISC.

### Plasma stability of ss-siRNA containing (*R*)-5′-methyl and (*S*)-5′-methyl-2′-*O*-MOE thymidine 5′-phosphate

**Figure 6.**
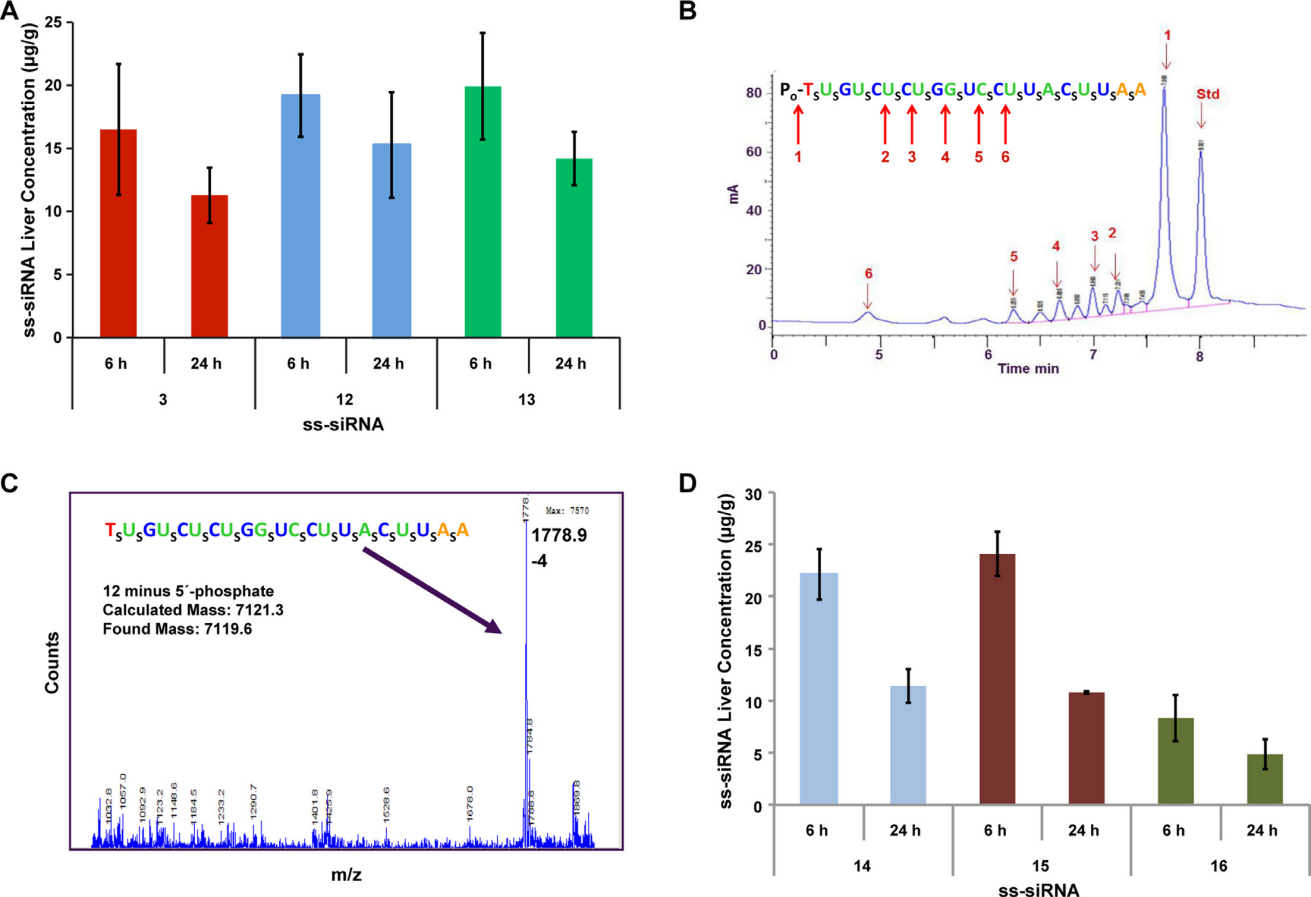
Identification of ss-siRNA metabolites extracted from mouse liver post-treatment using liquid chromatography-tandem mass spectrometry (LC-MS) analysis. (**A**) Levels of ss-siRNAs **12** and **13** minus 5′-phosphate in mouse liver at 6 and 24 h post treatment. (**B**) The liquid chromatography profiles of ss-siRNA **13** extracted from the liver after 24 h of dosing and shown is the relative abundance of the metabolites identified by mass spectrometry compared to the internal standard (Std.). Numbered arrows indicate the position of degradation sites in the ss-siRNA in relationship to the corresponding peaks from the liquid chromatography profile. (**C**) MS profile of the chromatographic peak corresponds to the peak 1 in (B). (**D**) Levels of ss-siRNAs **14–16** minus 5′-phosphate in the mouse liver at 6 and 24 h post treatment.

To further assess the effect of 5′-methyl substitution on metabolic stability of the 5′-phosphate in ss-siRNA, mice were injected with ss-siRNA **12** and **13** 25 mg kg^−1^ and sacrificed after 24 h. ss-siRNAs from the liver were extracted and analyzed by LC-MS to determine the total liver concentrations of the compounds (Figure 6A) and to identify any metabolites. Analysis of the ss-siRNAs extracted from the mouse liver showed the predominant metabolite observed for the ss-siRNA **12** and **13** consisted of the full-length compound absents the 5′-phosphate (Figure 6B and C). In fact, analysis of the ss-siRNA from studies with shorter treatment times indicated that the compound was completely dephosphorylated in as little as 6 h (data not shown). Importantly, these results indicated that the 5′-terminal 2′-*O*-MOE and alternating phosphorothioate (s) and 2′-OMe substitutions at the 5′-pole significantly enhanced the metabolic stability of the ss-siRNA *in vivo* (Figure 6B and C). Finally, these data suggest that 5′-methyl modification did not prevent 5′-de-phosphorylation of ss-siRNA in animals.

### Structure–activity relationship study to improve the metabolic stability of ss-siRNA containing 5′-substitued phosphate

**Figure 7.**
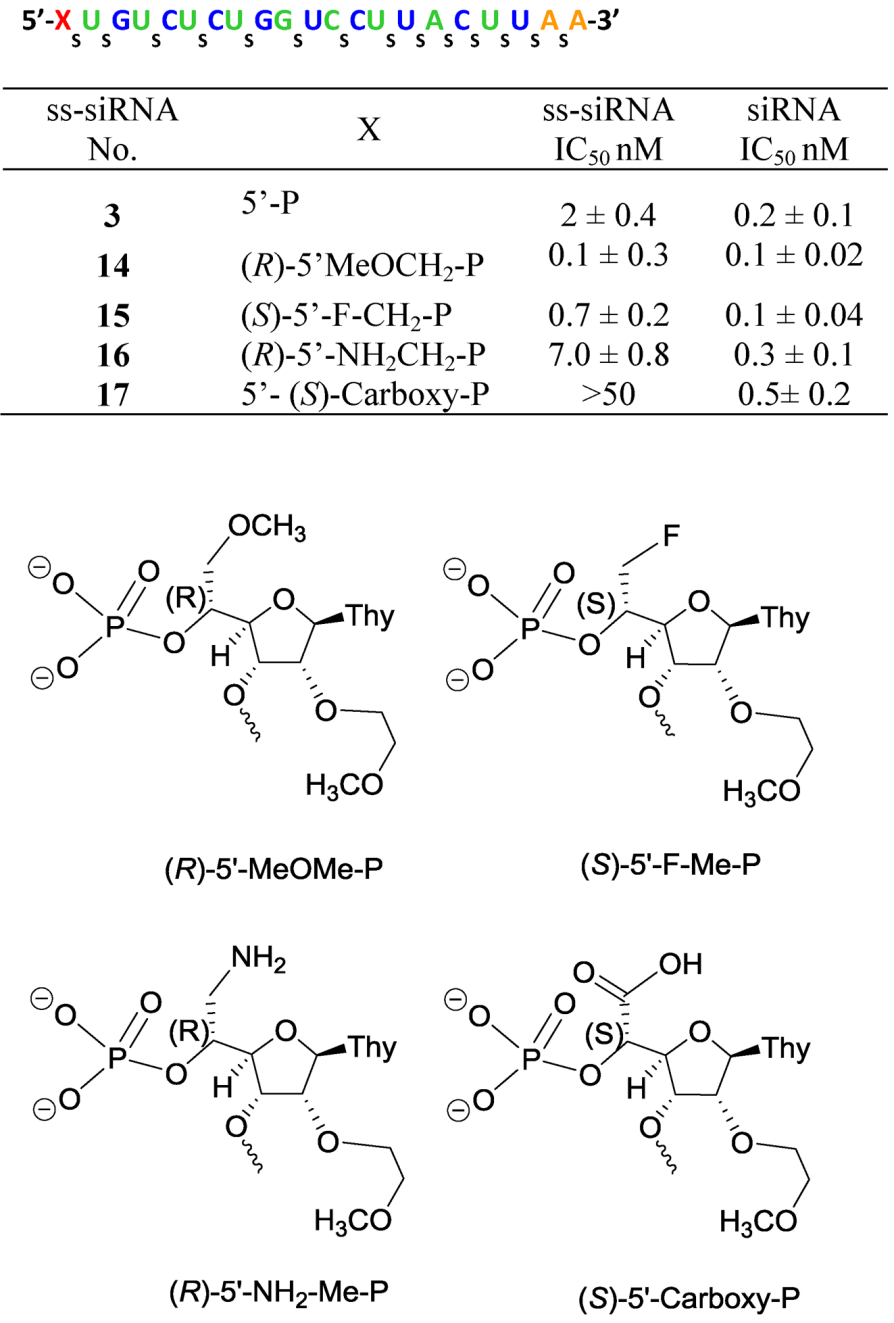
*In vitro* activity of 5′-modified PTEN ss-siRNA and siRNA in HeLa cells.

To further assess the effect of 5′-alkyl modifications on metabolic stability and activity of ss-siRNA, we synthesized 5′-methoxymethyl ss-siRNA (**14**; Figure 7) to increase steric bulk, 5′-fluormethyl ss-siRNA (**15**; Figure 7) to increase hydrophobicity, 5′-aminomethyl ss-siRNA (**16**; Figure 7) to introduce positive charge and 5′-carboxylate ss-siRNA (**17**; Figure 7) to introduce negative charge.

**Scheme 3.**
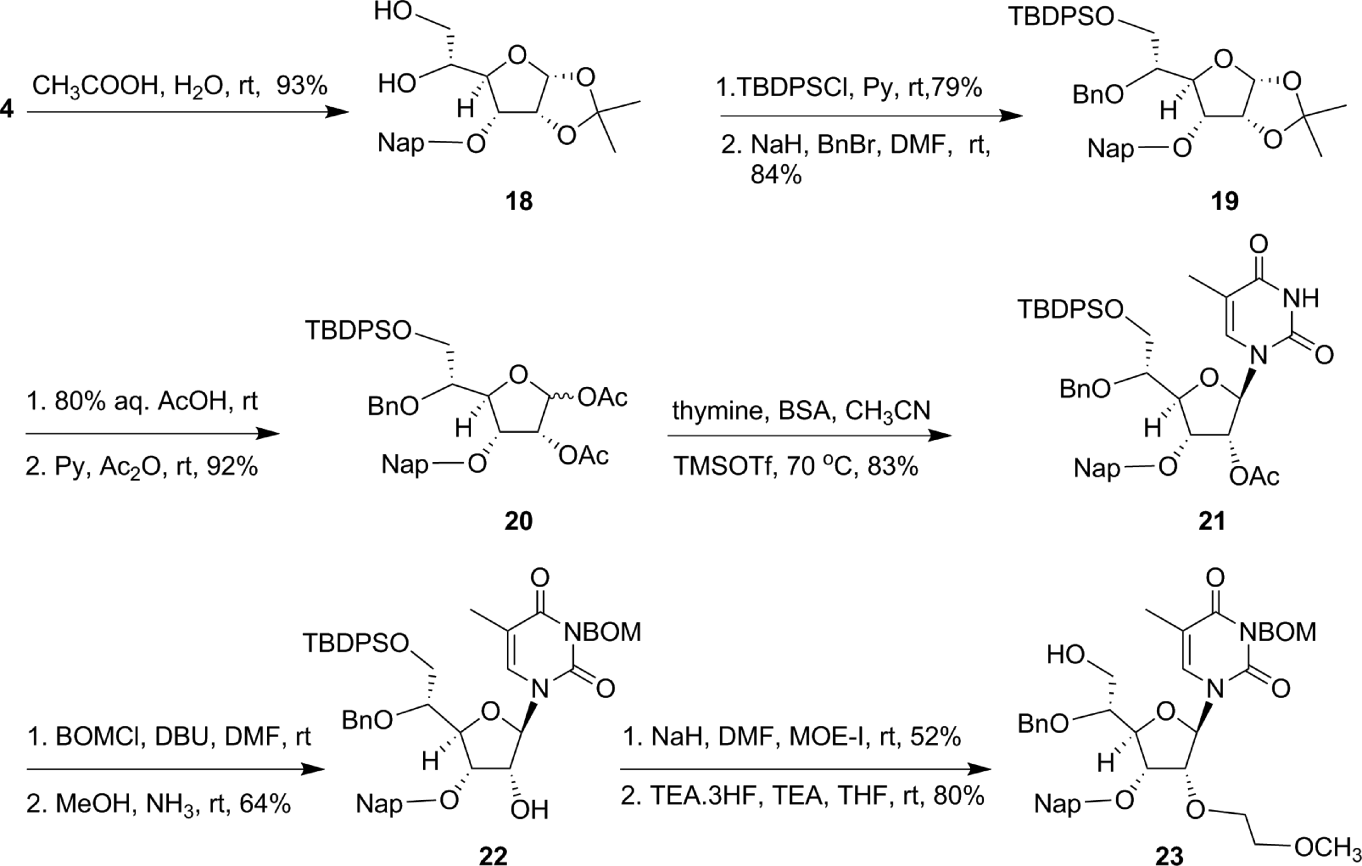
Synthesis of 5′-*O*-(benzyl)-(*S*)-5′-(hydroxymethyl)-2′-*O*-(2-methoxyethyl)-3′-*O*-(Nap)-thymidine (**23**); Bn: benzyl; TBDPS: *tert*-butyldiphenylsilyl.

**Scheme 4.**
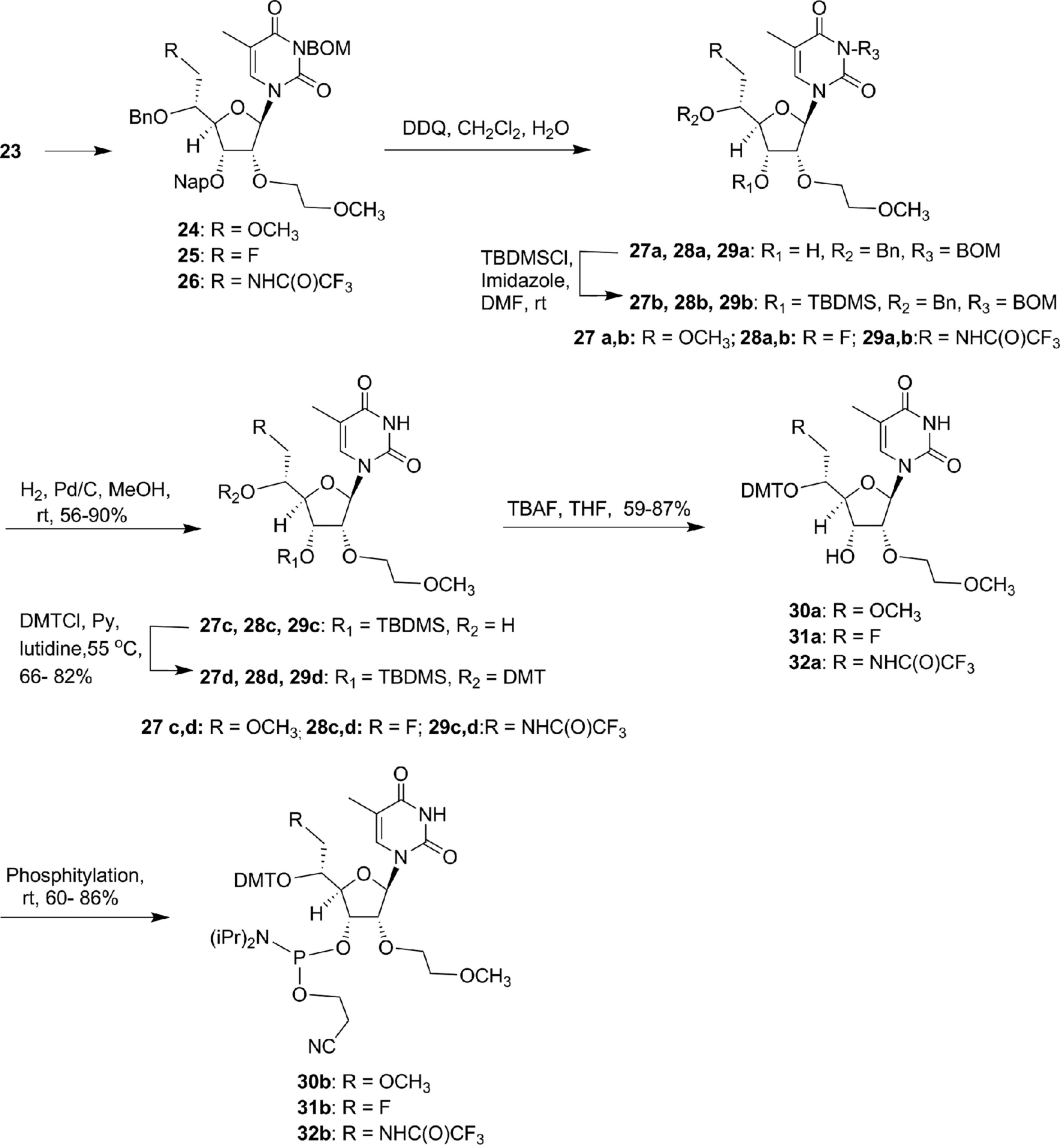
Synthesis of phosphoramidites **30b–32b**.

**Scheme 5.**
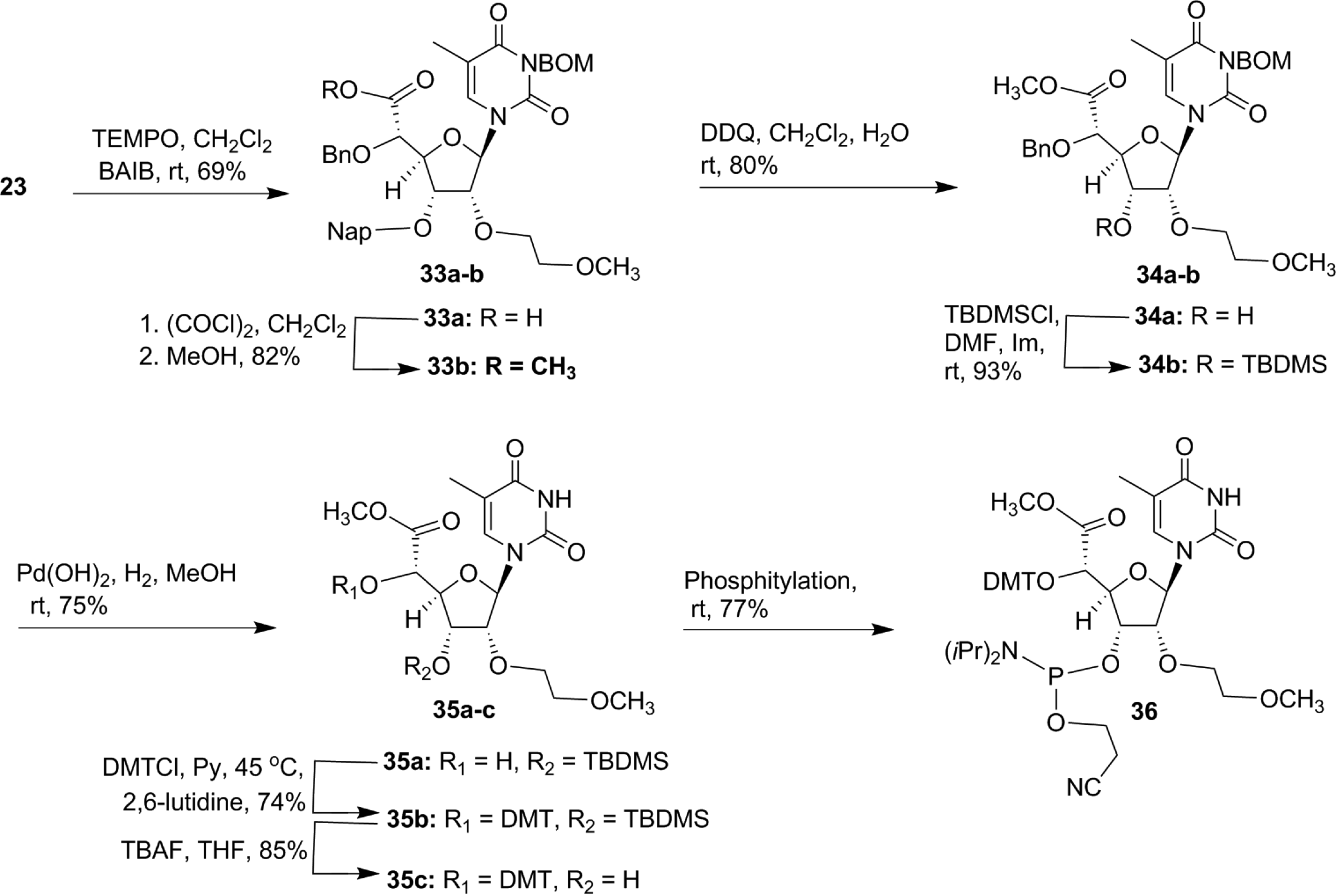
Synthesis of 5′-*O*-(4′,4′-dimethoxytrityl)-(*S*)-5′-(methoxycarbonyl)-2′-*O*-(2-methoxyethyl)-thymidine-3′-phosphoramidite **36**.

First we synthesized of 5′-methoxymethyl, 5′-fluormethyl and 5′-aminomethyl 2′-*O*-MOE-thymidine phosphoramidites **30b**, **31b** and **32b** (Scheme 4). An orthogonally protected 2′-*O*-(2-methoxyethyl)thymidine nucleoside **23** (Scheme 3) with 5′-hydroxymethyl substitution was identified as a versatile synthon to prepare all modified phosphoramidites **30b**, **31b** and **32b**. Synthesis of compound **23** was accomplished according to Scheme 3 (Supplementary data). Compound **23** was converted to 5′-methoxymethyl derivative **24** (Scheme 4; Supplementary data) with methyl iodide and NaH in DMF in good yield. Treatment of compound **23** with diethylaminosulfur trifluoride (DAST) in dichloromethane yielded 5′-fluoromethyl thymidine derivative **25** (Scheme 4; Supplementary data). The synthesis of 5′-aminomethyl thymidine analog **26** was also synthesized from compound **23** (Supplementary data). The thymidine analogs **24–26** were converted into their corresponding 3′-phosphoramidites **30b**, **31b** and **32b** according to Scheme 4 (Supplementary data). The 5′-methylcarboxylate-thymidine 3′-phosphoramidite **36** (Scheme 5) was used for the synthesis of 5′-carboxylate ss-siRNA **17** (Figure 7). The synthesis of phosphoramidite **36** was achieved as described in Scheme 5 (Supplementary data).

Syntheses of ss-siRNAs **14–17** (Figure 7) were achieved using the standard DNA synthesis procedure with appropriate modification based on the functional group present (22). In brief, after the synthesis of ss-siRNA **16** was completed, solid support was first treated with 50% triethylamine in acetonitrile containing 2-mercaptoethanol (1 M) for 45 min to remove the cyanoethyl group from the inter-nucleotide phosphodiesters and phosphorothioates. Then deprotection was completed by heating the solid support with aqueous ammonia containing 2-mercaptoethaol (1 M) at 55°C for 6 h. 2-mercaptoethanol was used to scavenge acyl and acrylonitrile groups generated during deprotection and to prevent the formation of acyl or cyanoethyl modified 5′-aminomethyl modified ss-siRNA. For the synthesis of ss-siRNA **17** the phosphoramidite **36** was used for incorporation of 5′-carboxy-2′-*O*-MOE thymidine residue at the 5′-end. A modified deprotection protocol was used to prevent the formation of carboxamide upon ammonia treatment. After the synthesis was completed, solid support bearing ss-siRNA **17** was treated with 50% piperidine in water and kept at room temperature for 12 h to convert 5′-methylcarboxylate to 5′-carboxylate. This was followed by treatment with 50% triethylamine in acetonitrile for 45 min to remove the cyanoethyl group from the inter-nucleotide phosphodiesters and phosphorothioates. The deprotection was completed by heating the solid support with aqueous ammonia at 55°C for 6 h. High-resolution MS MS analysis and isotopic distribution was used to confirm the presence of 5′-carboxylate functionality. The structural integrity of ss-siRNAs (**14–17**) was confirmed with LC MS analysis (Supplementary data).

We then tested these ss-siRNAs **14–17** in HeLa cells to assess their activities. HeLa cells were transfected with ss-siRNAs **14–17** using Lipofectamine 2000. Reduction of target mRNA was determined by qRT-PCR as previously described (9). Similar potencies were observed for 5′-methoxymethyl ss-siRNA **14** and fluoromethyl ss-siRNA **15** (Figure 7) and 5′-methyl ss-siRNA **12** (Figure 4). The amino methyl ss-siRNA **16** and 5′-carboxymethyl ss-siRNA **17** were less potent compared to the unmodified ss-siRNA **3** (Figures 7) or 5′-methyl ss-siRNA **12** (Figure 4). Interestingly, potency of the corresponding siRNAs was similar to the parent siRNA (Figure 7). These data suggest that the structure–activity relationship differs significantly between ss-siRNA and siRNA.

Next we investigated the effect of these modifications on the metabolic stability of ss-siRNA. Mice were dosed with 20 mg/kg of ss-siRNAs **14–16** in saline and sacrificed at 6 and 24 h after dosing. The ss-siRNA **17** was not included in this study because it was significantly less active in the cell culture experiments. Metabolites were extracted from the livers of the animals treated with ss-siRNAs **14–16** and analyzed by LC MS. The predominant metabolites observed for ss-siRNAs **14–16** were the full-length compound minus 5′-phosphate (Figure 6). The tissue levels of ss-siRNA **14–16** minus the 5′-phosphate (Figure 6D) were similar to parent ss-siRNA **3** (Figure 6A) with exception of the 5′-aminomethyl ss-siRNA **16** that was significantly less (8 μg/g for ss-siRNA **16** versus 17 μg/g at 6 h for ss-siRNA **3**). Although we do not have a clear explanation for the observed differences, these data suggest that small changes in chemical structure could influence the distribution of the ss-siRNA to the liver.

### Approach B: identification of methylenephosphonate analogs as metabolically stable phosphate mimic for ss-siRNA

**Figure 8.**
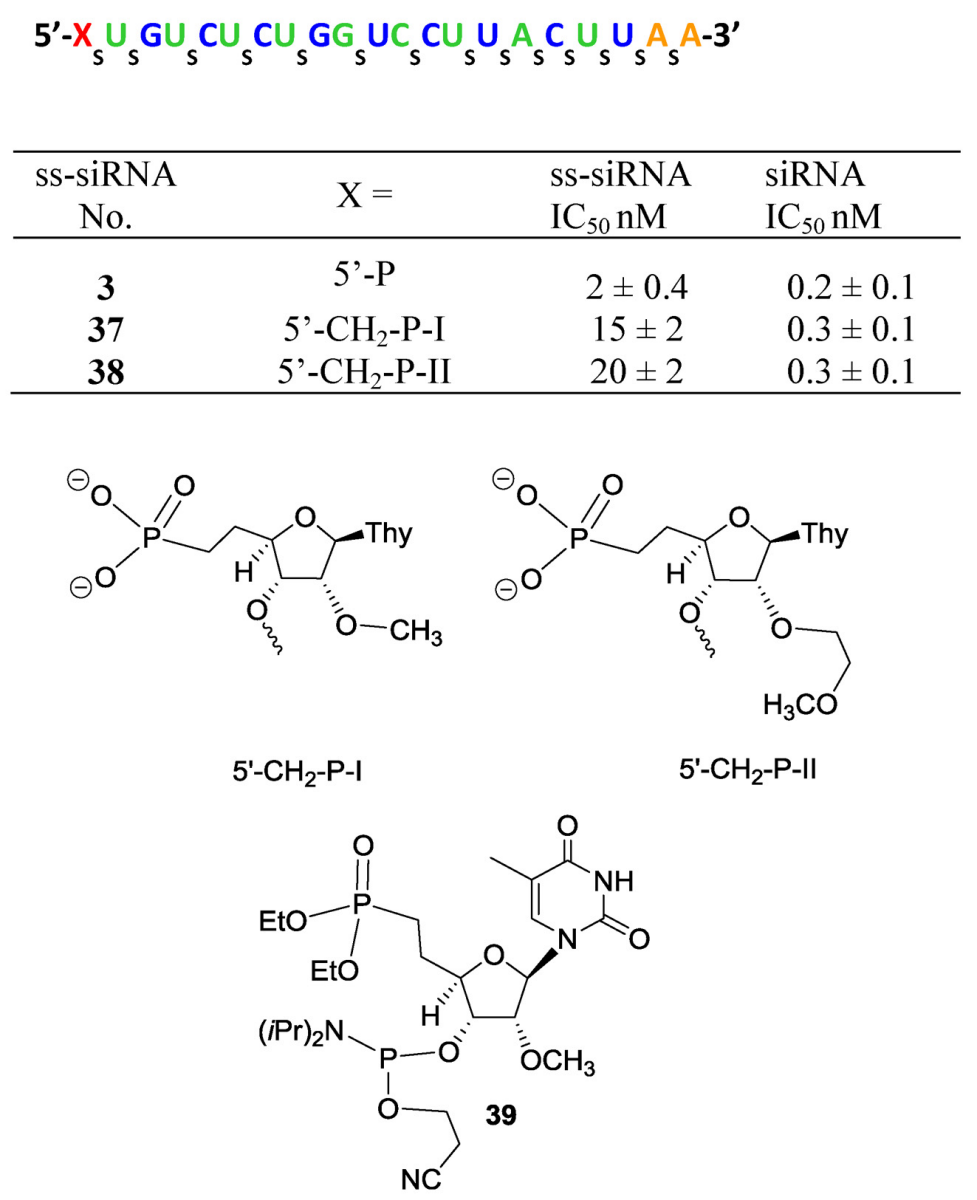
*In vitro* activity of 5′-modified PTEN ss-siRNA and siRNA.

**Scheme 6.**
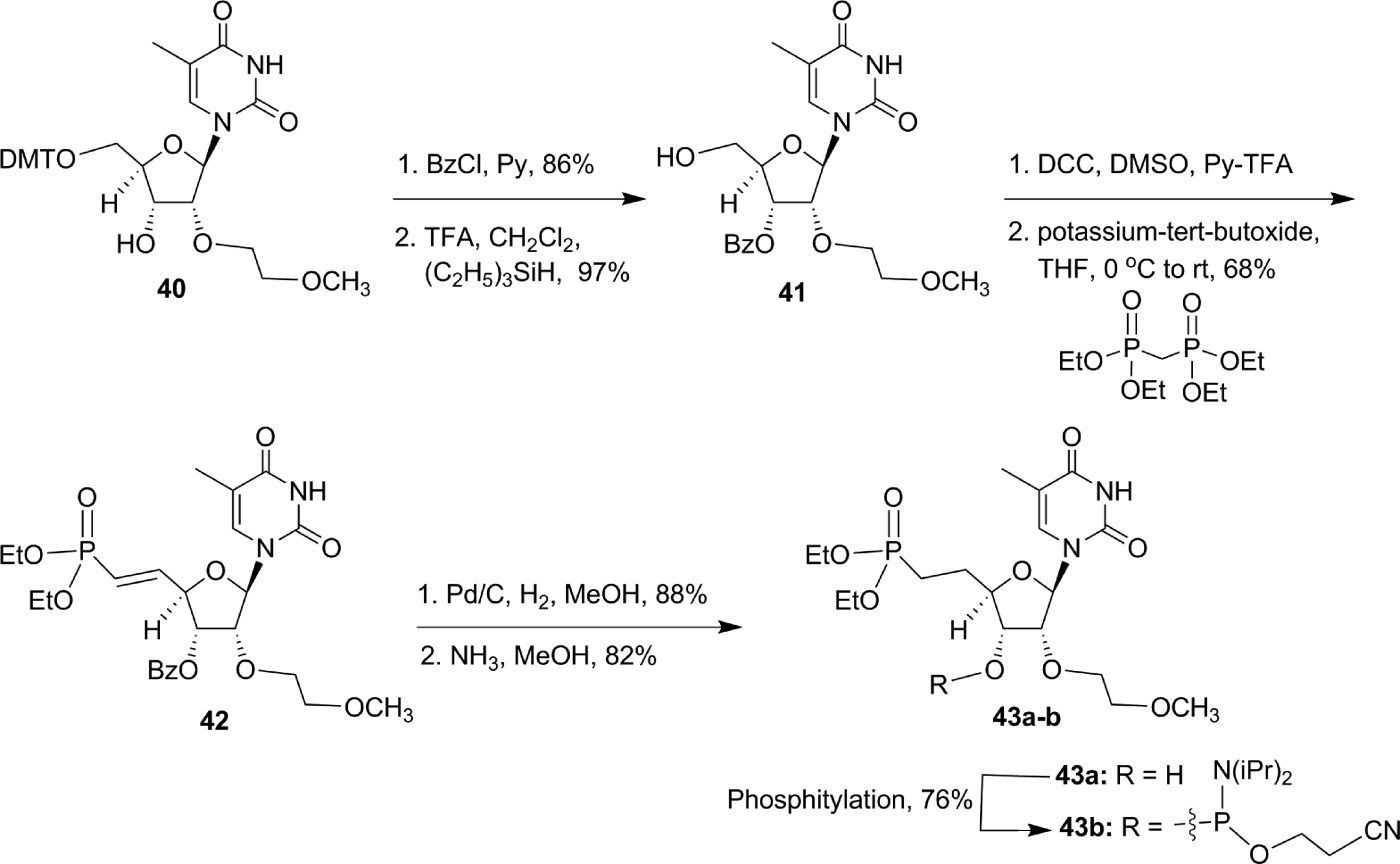
Synthesis of 5′-deoxy-5′-(diethylmethylenephosphonate)-2′-*O*-(2-methoxyethyl)-thymidine-3′-phosphoramidite **43b**.

Phosphonic acid (Figure 2) is a close analog of phosphate and it has a carbon–phosphorus bond instead of an oxygen–phosphorus bond of phosphate (23,24). The carbon–phosphorous bond in phosphonic acid, unlike phosphates, is not susceptible to the hydrolytic action of phosphatases under physiological conditions (23,24) and we envisaged that 5′-phosphonate on ss-siRNA would also be metabolically stable. However, phosphonic acid is expected to have decreased acidity relative to phosphate (24) due to the introduction of an electron–donating alkyl group in place of an oxygen (24). In addition one would expect a difference in the physical size and shape of phosphonic acid relative to phosphate (24). To test this hypothesis, we designed and synthesized ss-siRNAs **37** and **38** (Figure 8) where the nucleotide at position 1 was replaced with 2′-*O*-methyl-thymidine-5′-deoxy-5′-methylenephosphonate (5′-CH_2_-P-I) and 2′-*O*-(2-methoxyethyl)-thymidine-5′-deoxy-5′-methylenephosphonate (5′-CH_2_-P-II), respectively. 2′-*O*-methyl-5′-deoxy-5′-(diethylphosphonate)thymidine-3′-phosphoramidite **39** (Figure 8 and Supplementary data) was used to incorporate 5′-CH_2_-P-I at position 1 of ss-siRNA **37** (Figure 8). For incorporation of 5′-CH_2_P-II at the 5′-end of ss-siRNA **38** (Figure 8) the 2′-*O*-(2-methoxyethyl)-5′-deoxy-5′-(diethylphosphonate)thymidine-3′-phosphoramidite **43b** was used. The phosphoramidite **43b** was synthesized according to synthetic Scheme 6 (Supplementary data). Syntheses of ss-siRNAs **37–38** were achieved using standard DNA synthesis (22).

However, conventional deprotection condition (aqueous ammonia at 55°C) was not able to hydrolyze the ethyl ester of the 5′-deoxy-5′-methylenephosphonate used as a protecting group during ss-siRNA synthesis. In order to hydrolyze the ethyl ester from the 5′-phosphonic acid of ss-siRNAs **37** and **38** a post-synthetic method was developed. On treatment of solid support bearing ss-siRNAs **37–38** to a solution of iodotrimethylsilane in dichloromethane containing pyridine for 30 min at room temperature completely removed ethyl group from the 5′-deoxy-5′-methylenephophonic acid (25). The reaction was quenched with a solution of 50% triethylamine in acetonitrile containing 2-mercaptoethanol (1 M). The supernatant was decanted and the solid-support-bound ss-siRNAs were then treated with aqueous ammonia (28–30 wt%) containing 2-mercaptoethanol (1 M) to completely remove all the protecting groups. To our knowledge, this is the first synthesis of oligonucleotides containing 5′-deoxy-5′-methylenephosphonic acid using 5′-deoxy-5′-diethylmethylenephosphonate-nucleoside-3′-phosphoramidite. The mass and purity of ss-siRNAs (**37–38**) were confirmed by ion-pair LC MS analysis (Supplementary data).

ss-siRNAs **37–38** were tested in HeLa cells to assess the potency. ss-siRNAs **37–38** were 7–10-fold less active (Figure 8; IC_50_: ss-siRNA **37** 15 nM, ss-siRNA **38** 20 nM) in HeLa cells relative to parent ss-siRNA **3**. Conversely, the activities of corresponding siRNAs were similar to the parent siRNA (Figure 8). It is interesting to note that ss-siRNA **38** with a bulky 2′-*O*-MOE modification and ss-siRNA **37** with a 2′-*O*Me modification at position 1 showed similar potency. These data are consistent with the observation that the 2′-hydoxyl of nucleotide 1 of the guide strand does not appear to interact with human Ago-2 protein (16). We recently reported result of testing ss-siRNA **38** with a 5′-deoxy-5′-methylenephosphonic acid (5′-CH_2_-P) modification in mice (9). The ss-siRNA modestly inhibited the PTEN mRNA in the liver (9). LC MS analysis of ss-siRNA **38** extracted from the liver of animals showed the compound containing the 5′-CH_2_-P modification (9). These results demonstrated that ss-siRNA containing metabolically stable phosphate analog inhibits gene expression in animals.

### Identification of 5′-methylenephosphonic acid analogs with conformation and electronics similar to 5′-natural phosphate

The *in vitro* potency of ss-siRNAs **38** containing 5′-CH_2_P was significantly lower compared to ss-siRNA **3**. We hypothesized that this difference in potency could be a result of the conformational and stereoelectronic differences between methylene phosphonate and phosphate. The crystal structure of Ago-2 showed that side chains of amino acids Y529, K533, N545 and K566 interact with 5′-phosphate of the guide RNA (Figure 3) through formation of hydrogen bonds and salt bridges (12). It was possible that conformational and stereoelectronic differences between methylenephosphonate and phosphate may disrupt these interactions and could contribute to the observed differences in the activity of ss-siRNAs. To address the stereoelectronic limitations of 5′-CH_2_P modification we designed and synthesized several ss-siRNAs containing 5′-CH_2_P analogs with different electronic and conformational properties.

### 5′-CH_2_P-analogs to probe the effect of altering electronic properties

**Figure 9.**
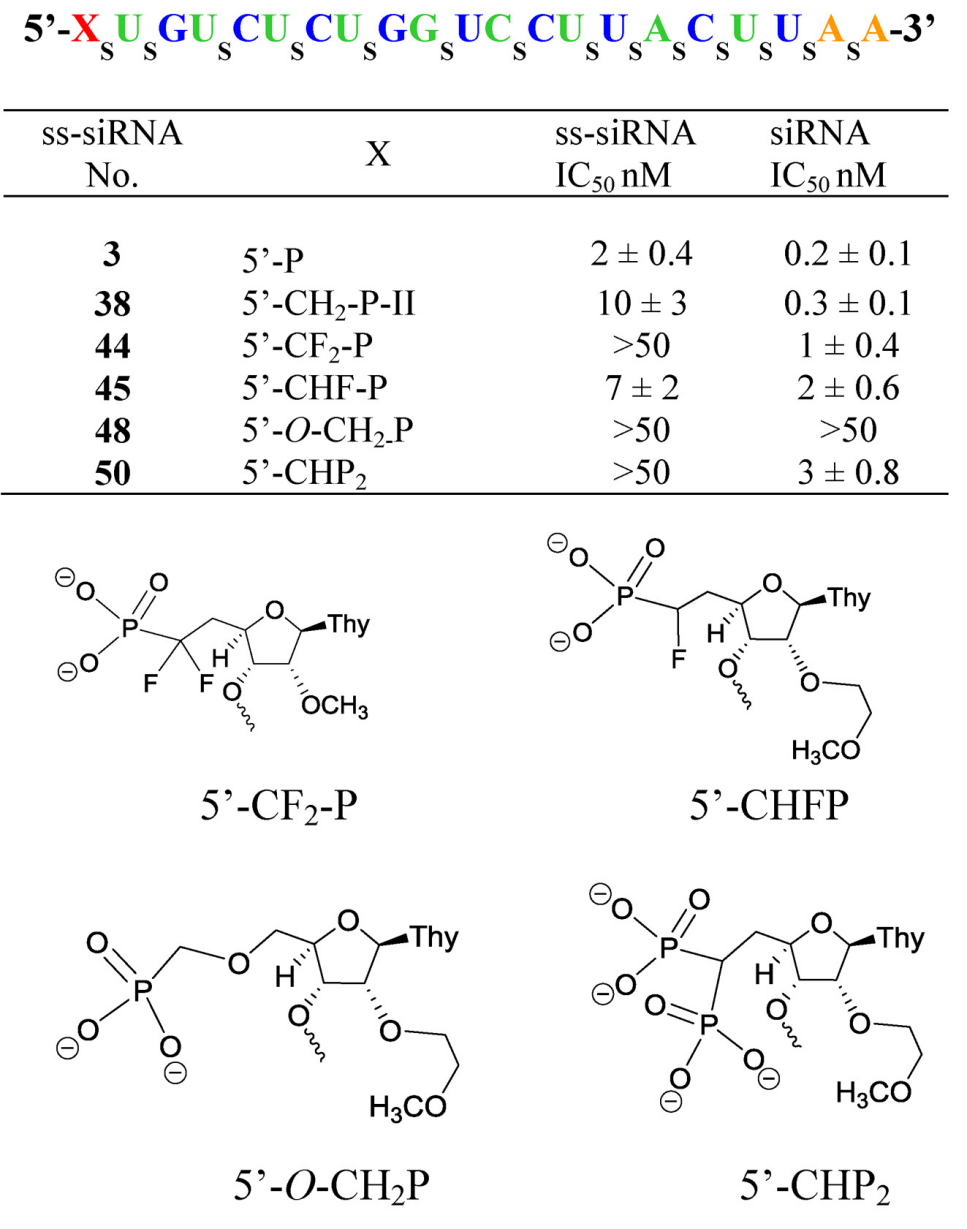
*In vitro* activity of 5′-modified PTEN ss-siRNA and siRNA.

**Figure 10.**
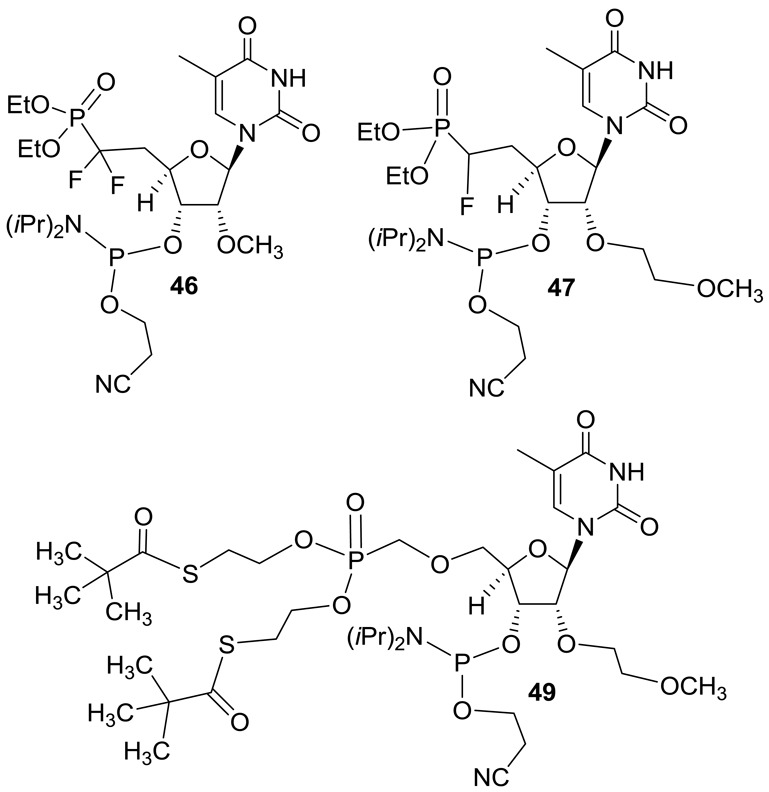
Structures of phosphoramidites **46–47** and **49**.

Previous work suggested that *α*-fluoro and *α*,*α*-difluoromethyl phosphonates can serve as isosteric and isopolar analogs of phosphate esters (26,27). ss-siRNA **44** (Figure 9; 5′-CF_2_-P) containing *α*,*α*-difluoromethylenephosphonate and ss-siRNA **45** (Figure 9, 5′-CHF-P) containing *α*-fluoromethylenephosphonate at 5′-end were synthesized to investigate the effect of altering electronics. To introduce*α*,*α*-difluoromethylenephosphonate at the 5′-end of ss-siRNA we synthesized 5′-deoxy-5′-*α*,*α*-difluoromethylenediethoxyphosphonate-2′-*O*-methylthymidine-3′-phosphoramidite **46** (Figure 10 and Supplementary data) using a modified version of the reported procedure (23). For the incorporation of *α*-fluoromethylenephosphonate at 5′-end of ss-siRNA we synthesized 5′-deoxy-5′-*α*-fluoromethylenediethoxyphosphonate-2′-*O*-(2-methoxyethyl) thymidine-3′-phosphoramidite **47** (Figure 10 and Supplementary data) (28). Syntheses of ss-siRNAs **44–45** were achieved using the same procedure used for the synthesis of ss-siRNA **38**. The ss-siRNAs **44–45** were characterized by ion-pair LC MS analysis (Supplementary data).

ss-siRNAs **44–45** were transfected to HeLa cells using Lipofectamine 2000. Reduction of target mRNA was determined by qRT-PCR as previously described. The ss-siRNA **44** containing 5′-CF_2_-P modification was less potent (Figure 9; IC_50_ > 50 nM) relative to parent ss-siRNA **3** as well as 5′-CH_2_P ss-siRNA **38** (Figure 9; IC_50_ 20 nM). Interestingly, 5′-CHF-P ss-siRNA **45** was more potent (Figure 9; IC_50_ 7 nM) relative to 5′-CH_2_P ss-siRNA 38 (Figure 9; IC_50_ 20 nM). These data suggest that shifting electronics of the 5′-CH_2_P closer to a phosphate by fluorination at α-position improved the potency of ss-siRNA. Albeit, replacing oxygen atom of the 5′-phophate with an isopolar CF_2_ group significantly reduced activity of the ss-siRNA but the impact on siRNA activity was only 5-fold.

### 5′-CH_2_P-analogs to investigate the effect of altering spatial disposition

The crystal structure of guide strand of siRNA bound to MID domain of human Ago-2 protein suggested that precise positioning of 5′-phosphate at the phosphate binding pocket of the enzyme is critical for slicer activity (12). In order to confirm, similar positioning of 5′-phosphate is required for ss-siRNA activity, we designed 5′-*O*-CH_2_P ss-siRNA **48** (Figure 9) where we introduce one oxygen atom between methylenephosphonate at 5′-end of ss-siRNA. We envisaged that extending the distance of methylenephosphonate from the 5′-end of ss-siRNA will perturb its positioning in the 5′-binding pocket of Ago-2 and this structural change would affect the slicer activity. The 2′-*O*-(2-methoxyethyl)-5′-*O*-(bis-(*S*-(pivaloyl-2-thioethyl)-methylenephophonate-thymidine-3′-phosphoramidite **49** (Figure 10 and Supplementary data) was synthesized to incorporate 5′-*O*-CH_2_P modification at the 5′-end of the ss-siRNA (29).

We used base labile *S*-(pivaloyl)-2-thioethyl (30) protecting group for 5′-*O*-CH_2_P protection during ss-siRNA synthesis and it was conveniently removed during aqueous ammonia treatment. In brief, after ss-siRNA **48** synthesis was completed (22), the solid support was suspended in aqueous ammonia containing 1-M 2-meraptoethaol and heated at 55°C for 6 h. The base labile group from the 5′-*O*-CH_2_P was completely removed from the ss-siRNA. To our knowledge, this is the first report of synthesis of 5′-*O*-CH_2_P-modified oligonucleotides using base labile protecting group for the 5′-*O*-methylenephophonate. The ss-siRNAs **48** was well characterized by ion-pair LC MS analysis (Supplementary data).

The activity of ss-siRNAs **48** was tested in HeLa cells where it was found to be less potent (Figure 9; IC_50_ > 50 nM) relative to parent ss-siRNA **3** as well as 5′-CH_2_P ss-siRNA **38** (Figure 8; IC_50_ 20 nM). These data clearly suggest that, like siRNA, precise positioning of 5′-phosphate at the binding pocket is critical and any small changes in positioning have significant impact on the activity of ss-siRNA.

### 5′-CH_2_P-analogs to probe the effect of altering charge density, steric crowding and spatial positioning

**Scheme 7.**
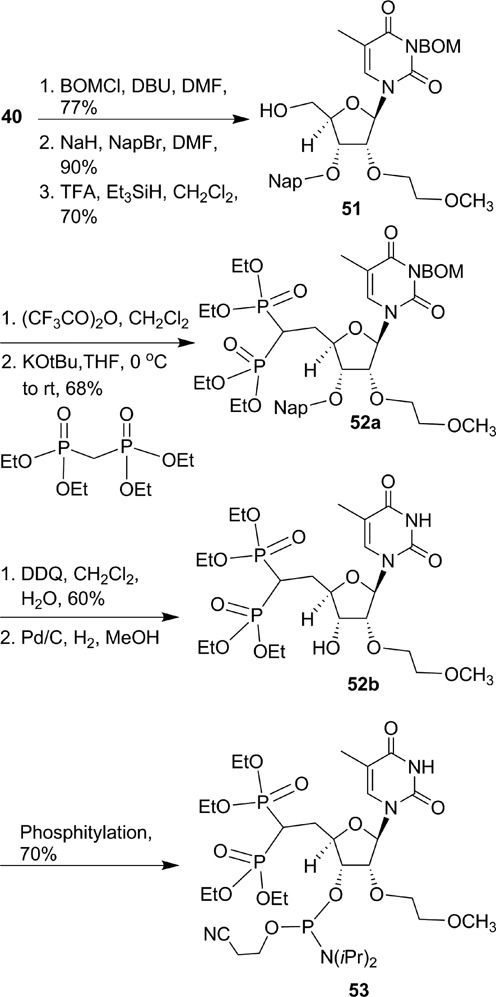
Synthesis of 5′-deoxy-5′-(tetraethyl methylenebis(phosphonate))-2′-*O*-(2-methoxyethyl)-thymidine-3′-phosphoramidite **53**.

The crystal structure of the full-length human Ago-2 bound to the guide strand of siRNA showed that the 5′-phosphate forms electrostatic and hydrogen-bond interactions with amino groups of lysines K533 and K566 (Figure 3) (12). We hypothesized that increased charge density around the 5′-CH_2_P moiety will enhance these interactions and binding of the ss-siRNA to Ago-2 resulting in improved potency. In order to test this hypothesis we designed and synthesized 5′-deoxy-5′-bisphosphonate (5′-CHP_2_) ss-siRNA **50** (Figure 9). We first synthesized 5′-deoxy-5′-(tetraethylbisphosphonate)methylene-2′-*O*-(2-methoxyethyl)-thymidine-3′-phosphoramidite **53** according to synthetic Scheme 7 (Supplementary data) (31). Synthesis of 5′-deoxy-5′-bisphophonate ss-siRNA **50** was accomplished using the synthesis procedure used for the synthesis of ss-siRNA **38**. The ss-siRNA was fully characterized by LC MS analysis (Supplementary data). ss-siRNA **50** containing 5′-CHP_2_ modification was less potent (Figure 9; IC_50_ > 50 nM) relative to either the parent ss-siRNA **3** or 5′-CH_2_P ss-siRNA **38** (Figure 9; IC_50_ 20 nM). The corresponding siRNA was also 10-fold less active (Figure 9). These data clearly demonstrate that altering charge density, steric crowding and positioning of 5′-phosphate significantly affects the activity of ss-siRNA.

### Identification of conformationally rigid vinylphosphonate as stereoelectronic substitute for 5′-phosphate of ss-siRNA with high metabolic stability

**Figure 11.**
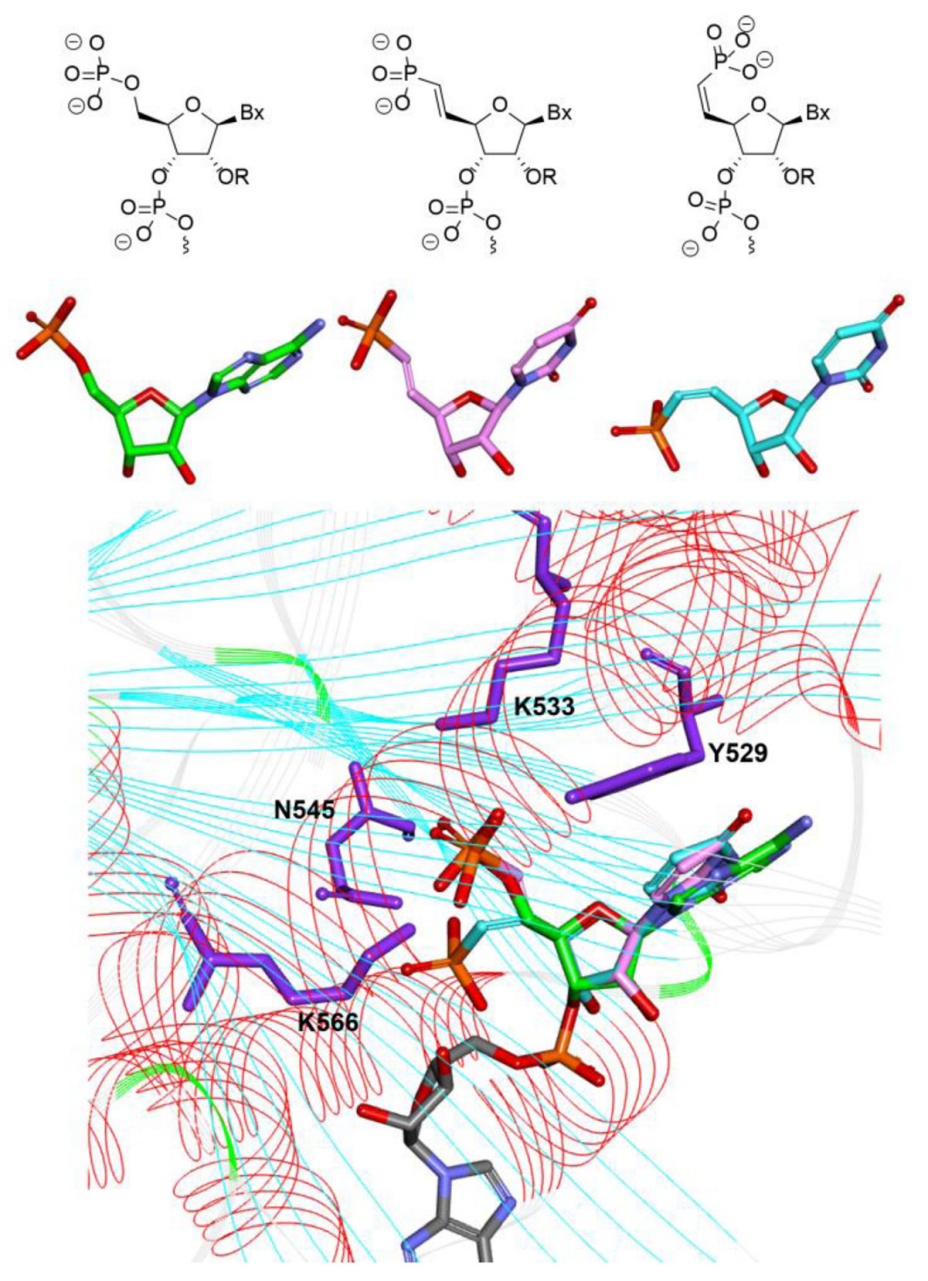
Structure of 5′-phosphate (green), (*E*)-5′-vinyl phosphonate (pink), (*Z*)-5′-vinylphosphonate (blue) and overlaid structures of 5′-phosphate (green), (*E*)-5′-Vinyl phosphonate.

Results from the SAR studies with different substituted methylenephosphonate analogs suggest that electronic and spatial positioning of the phosphate is critical for ss-siRNA activity. Rotation about the exocyclic C4′-C5′ bond plays a crucial role in positioning of 5′-phosphate in the correct alignment within the Ago2 binding pocket. The O_4′_-C_4′_-C5′-O5′-torsion angle structure of 5′-phosphate of the guide strand of ss-siRNA is in a *+sc* conformation (Figure 5A) and exhibits rigid conformation due to 5′-O-4′-O gauche effect. To mimic the stereoelectronic features of the 5′-phosphate moiety bound in the Ago-2 crystal structure, we next evaluated the *cis*- and *trans*-unsaturated analogs (Figure 11). Structural model of *cis* and *trans* vinyl phosphonate nucleoside showed that the *trans* vinylphosphonate can assume a conformation similar to that of the 5′-phosphate in the Ago-2 crystal structure (Figure 11). Furthermore, unsaturated moieties were previously shown to serve as structural and functional mimics of oxygen in nucleic acid structures (32,33). In addition, we also prepared the fluorinated vinyl phosphonate analog as this modification could also serve as an isopolar and isosteric phosphate ester mimic for ss-siRNA.

**Figure 12.**
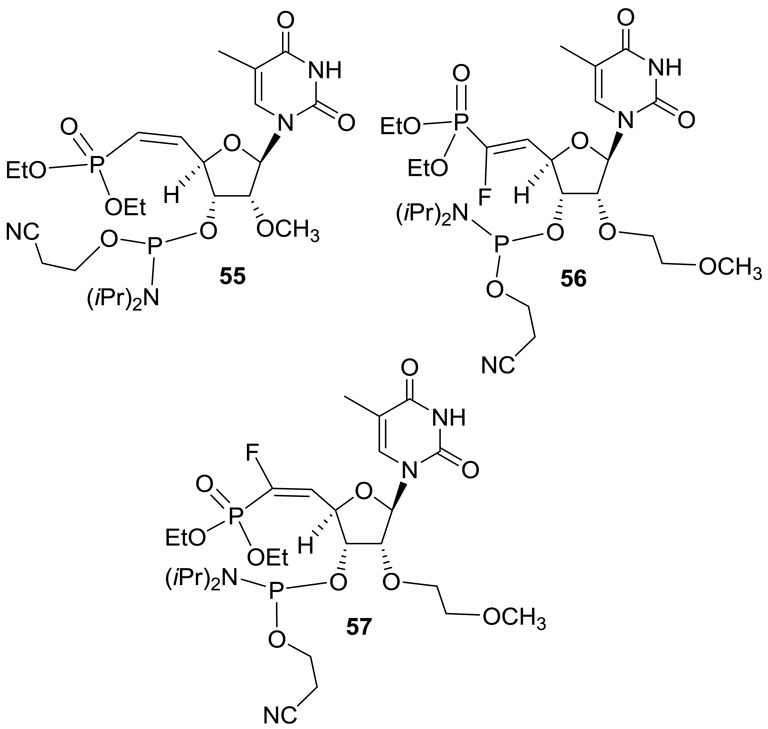
Structures of phosphoramidites **55–57**.

**Scheme 8.**
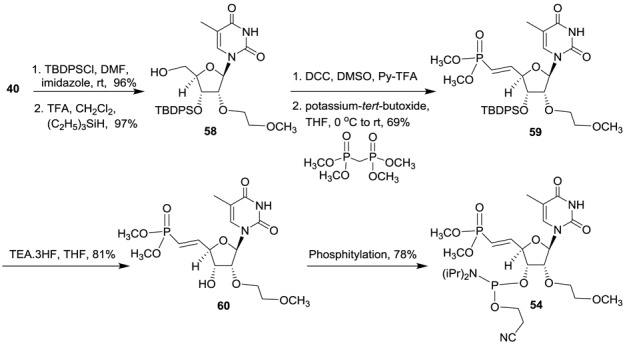
Synthesis of 5′-deoxy-(*E*)-5′-(dimethylvinylphosphonate)-2′-*O*-(2-methoxyethyl)-thymidine-3′-phosphoramidite **54**.

Synthesis of 5′-deoxy-(*E*)-5′-vinyl(dimethylphosphonate)-2′-*O*-(2-methoxyethyl)-thymidine-3′-phosphoramidite **54** was accomplished according to Scheme 8 (Supplementary data) (28). Similarly 5′-deoxy-5′-(*Z*)-vinyl(dimethylphosphonate)-2′-*O*-(2-methoxyethyl)-thymidine-3′-phosphoramidite **55** was also synthesized (Figure 12 and Supplementary data) (28). We also synthesized 5′-deoxy-(*E*)-5′-fluorovinyl(dimethylphosphonate)-2′-*O*-(2-methoxyethyl)-thymidine-3′-phosphoramidite **56** (Figure 12 and Supplementary data) (34,35) and 5′-deoxy-(*Z*)-5′-fluorovinyl(dimethylphosphonate)-2′-*O*-(2-methyl)-thymidine-3′-phosphoramidite **57** (Figure 12 and Supplementary data) using reported methods (36,37).

**Figure 13.**
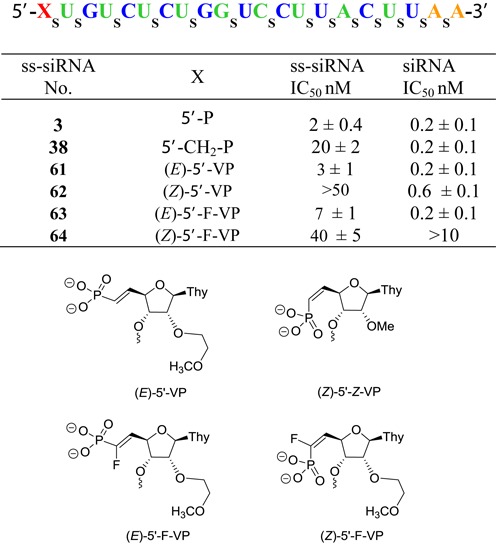
*In vitro* activity of 5′-modified PTEN ss-siRNA and siRNA.

The ss-siRNAs **61–64** (Figure 13) were synthesized using the synthetic procedure developed for the synthesis of ss-siRNA **38**. It is noteworthy that synthesis of ss-siRNA **61** using ethyl protecting group for the (*E*)-5′-vinylphosphonate gave poor yield after deprotection using TMSI/pyridine. However, significantly greater yields were achieved using phosphoramidite **54** (Scheme 8) with methyl protected (*E*)-5′-vinylphosphonate. ss-siRNAs were well characterized by ion-pair HPLC coupled mass spectroscopy (Supporting Information).

ss-siRNAs and corresponding siRNAs **61–64** (Figure 13) were tested in HeLa cells to evaluate the effect of the 5′-vinylphosphate analogs on potency. The ss-siRNA **61** containing the 5′-trans-vinylphosphonate ((*E*)-5′-VP) modification exhibited a potency (IC_50_ 3 nM; Figure 13) similar to parent ss-siRNA **3** (IC_50_ 2 nM; Figure 13), whereas the potency of the ss-siRNA containing the 5′-*cis*-vinylphosphonate ((*Z*)-5′-VP) **62** (IC_50_ > 50 nM; Figure 13) was significantly lower. Similar effect of stereoisomer on potency was observed with fluorinated 5′-vinylphosphonate ss-siRNAs. The ss-siRNA **63** containing 5′-*trans*-fluorovinylphosphonate ((*E*)-5′-F-VP) modification showed higher potency (IC_50_ 7 nM; Figure 13) relative to 5′-*cis*-vinylphosphonate ((*Z*)-5′-VP) **64** (IC_50_ 40 nM; Figure 13). It is surprising that ss-siRNA **63** was 7-fold less potent than ss-siRNA **61** given that fluorination of the alpha carbon of the 5′-VP modification (38,39) was expected to be isoelectronic compared to the phosphate. These data demonstrate that the factors other than electronics are contributing to the activity of (*E*)-5′-VP ss-siRNA. Specifically the steric resemblance of the (*E*)-5′-VP to phosphate esters positioned within the phosphate binding pocket of Ago2 (Figure 3).

### (*E*)-5′-VP-ss-siRNA is active in animal and activity is general for other targets

**Figure 14.**
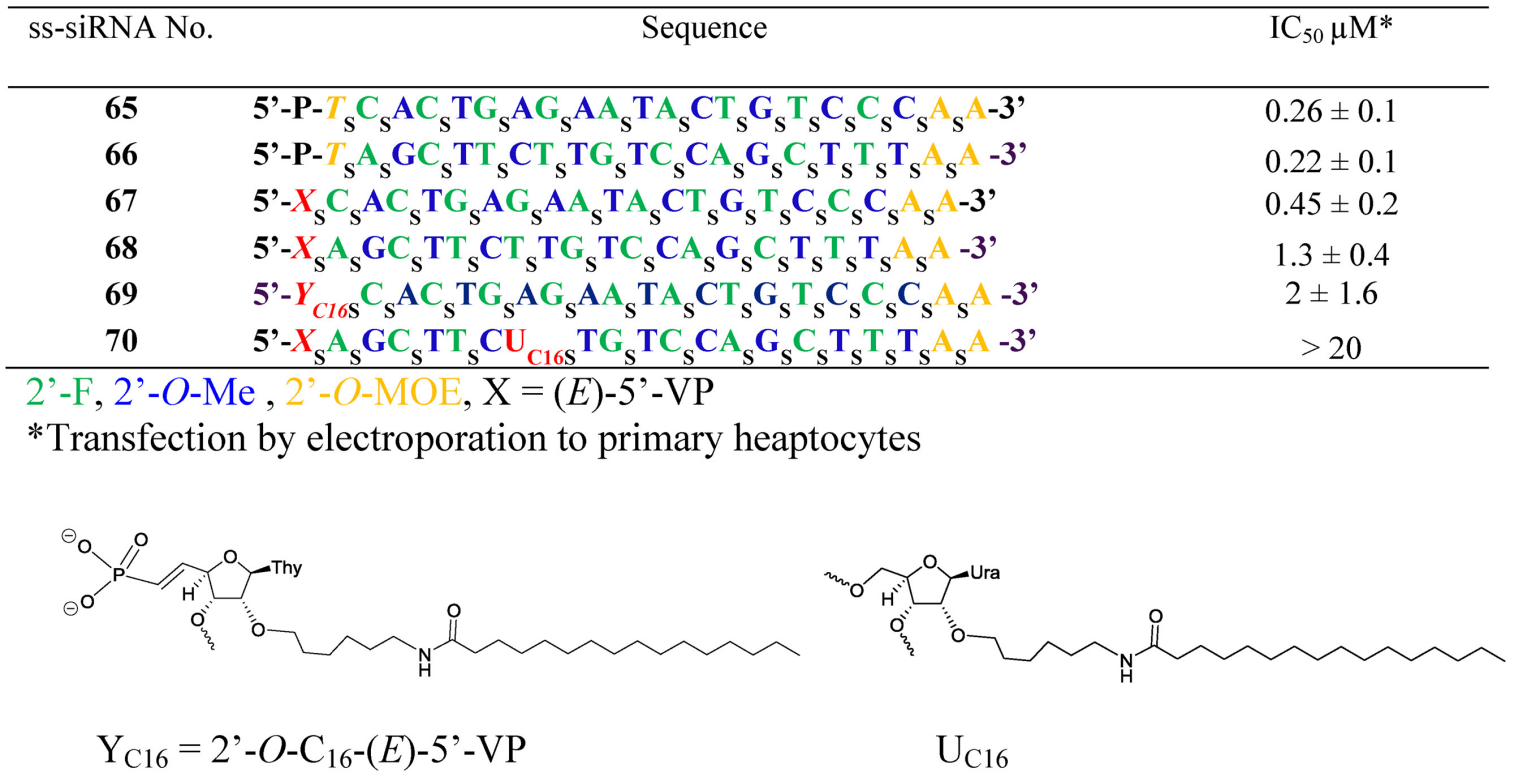
*In vitro* activity of (*E*)-5′-VP modified ss-siRNA targeting ApoC III mRNA.

We recently reported the *in vitro* and *in vivo* activity of (*E*)-5′-VP ss-siRNAs targeting PTEN, Factor VII and ApoC III mRNAs (9). The potency of ApoC III ss-siRNA reported was very modest. In order to identify more potent ss-siRNAs we designed 320 new ss-siRNAs containing 5′-phosphate targeting human ApoC III mRNA (18). ss-siRNAs were transfected to primary hepatocytes isolated from transgenic mice by electroporation. The ApoC III mRNA reduction was determined by qRT-PCR and IC_50_ curves and values were generated (data not shown). The ss-siRNAs **65** (Figure 14; IC_50_ 0.26 μM) and **66** (Figure 14; IC_50_ 0.22 μM) were identified as lead compounds for 5′-VP modification and corresponding (*E*)**-**5′-VP ss-siRNAs **67** and **68** (Figure 14) were synthesized using the similar procedure used for the synthesis of ss-siRNA **61**. The potency of ss-siRNA **67** (Figure 14; IC_50_ 0.45 μM) and **68** (Figure 14; IC_50_ 1.3 μM) was determined in hepatocytes. Interestingly **(*E*)-**5′-VP ss-siRNA **67** was 2-fold less potent than parent 5′-P ss-siRNA **65**. The potency difference was higher (6-fold) for (*E*)-5′-VP ss-siRNA **68** relative to parent 5′-P ss-siRNA **66** (Figure 14).

**Figure 15.**
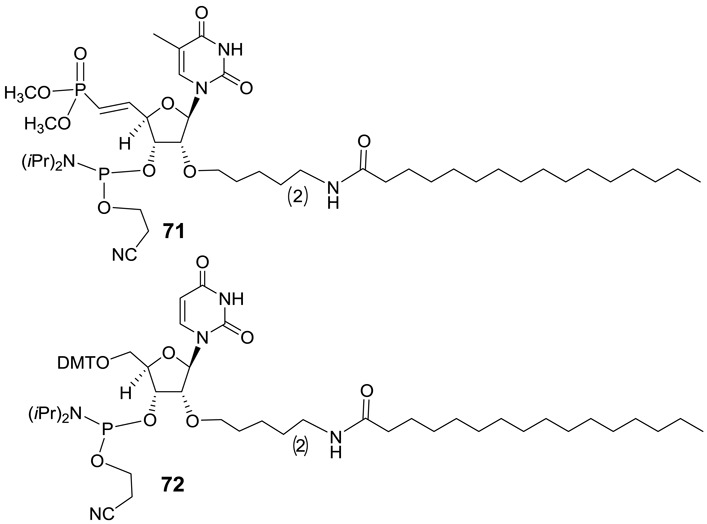
Structures of phosphoramidites **71–72**.

Previous studies have shown that ss-siRNA containing C_16_ modification exhibited greater *in vivo* activity compared to the unconjugated ss-siRNA (9). We synthesized C_16_ analog of ss-siRNAs **67** (Figure 14; **(*E*)-**5′-VP-C16 ss-siRNA **69**) and **68** (Figure 14; **(*E*)-**5′-VP-C16 ss-siRNA **70**) in order to improve their *in vivo* activity. It is worthwhile to note that ss-siRNA synthesis of ss-siRNA **69** needs a 3′-phosphoramidite containing 2′-*O*-C_16_-modified thymidine-dimethyl vinylphosphonate **71** (Figure 15 and Supplementary data). The synthesis of 3′-phosphoramidite **71** was accomplished (Supplementary data). Similarly for the synthesis of ss-siRNA **70** needed 2′-*O*-C_16_-uridine-3′-phosphoramidite **72** (Figure 15 and Supplementary data). The 3′-phosphoramidite **72** was also synthesized as shown in Supplementary data. The ss-siRNAs **69** and **70** were synthesized using the general procedure described for **(*E*)-**5′-VP ss-siRNA.

First, we determined the *in vitro* potency of ss-siRNAs **69** and **70** in ApoC III transgenic mouse primary hepatocytes by transfecting with electroporation. Interestingly, potency of **(*E*)-**5′-VP-C_16_ ss-siRNAs **69** (Figure 14; IC_50_ 2 μM) was 4-fold lower than corresponding the **(*E*)-**5′-VP ss-siRNA **67** (Figure 14; IC_50_ 0.45 μM). Additionally, the potency of **(*E*)-**5′-VP-C_16_ ss-siRNA **70** (Figure 14; IC_50_ > 20 μM) was more than 15-fold lower compared to the corresponding **(*E*)-**5′-VP ss-siRNA **68** (Figure 14; IC_50_ 1.3 μM). These data suggest that the position of a bulky substitution influenced the intrinsic potency of ss-siRNA and position 1 appears to be more amenable for bulky 2′ modification than internal position of ss-siRNA.

**Figure 16.**
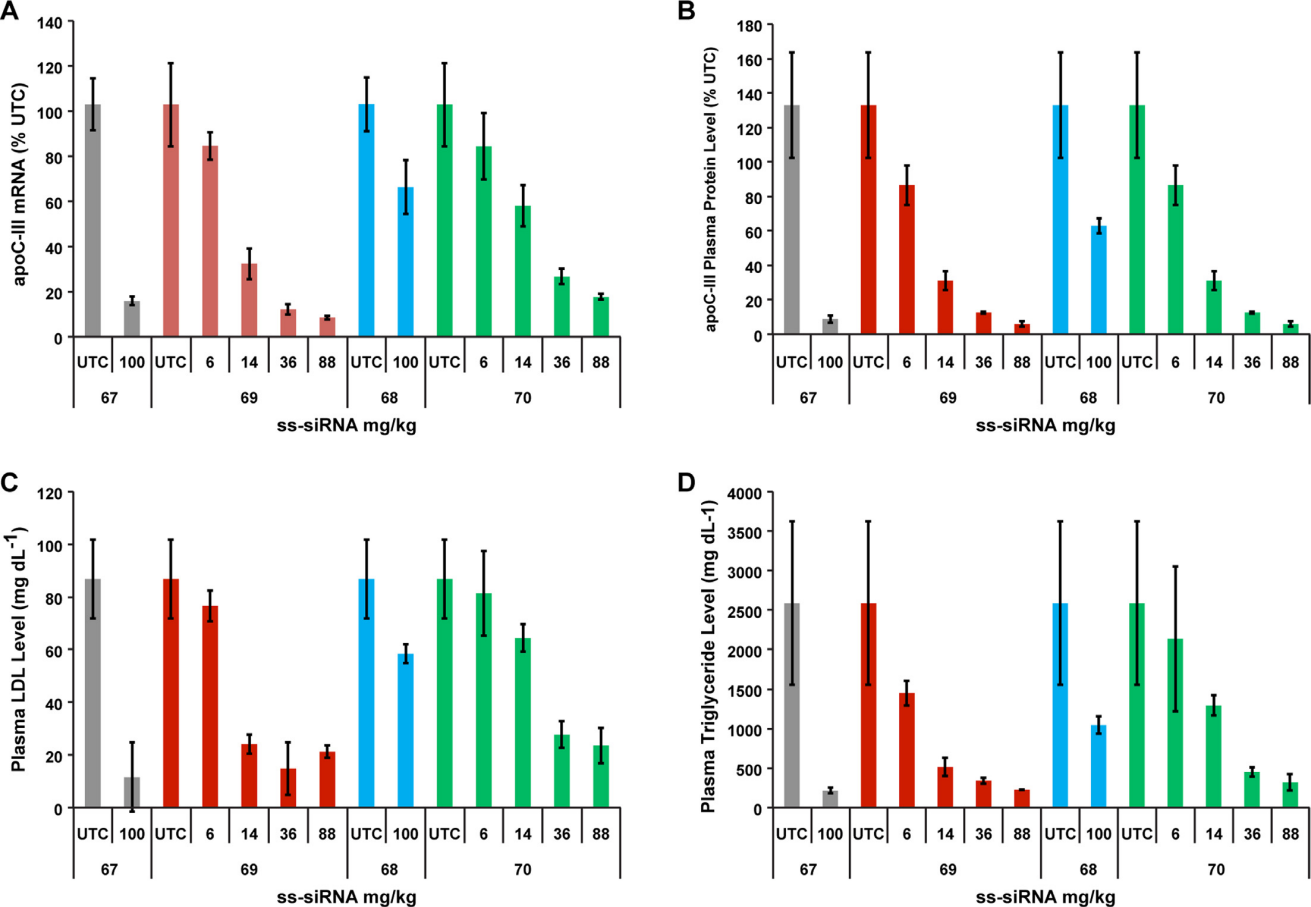
*In vivo* activities of modified human ApoC III ss-siRNAs following multiple dose administration. (**A**) Dose-dependent reduction of human ApoC III mRNA from the liver of transgenic mice (*n* = 4) treated with ss-siRNA **67–70** by subcutaneous administration. The ss-siRNAs **69** and **70** were dosed 3, 7, 18 and 44 mg kg^−1^ two times a week for 3 weeks and the doses reported are for per week. ss-siRNAs **67** and **68** were dosed 100 mg kg^−1^ fractionated (25 mg kg^−1^ twice a day for 1 day, wait 2 days then dose 25 mg kg^−1^ twice a day for 1 day) for 3 weeks and doses reported are for per week. (**B**) Dose-dependent reduction of ApoC III protein from the plasma of transgenic mice (*n* = 4) treated with ss-siRNA **67–70**. (**C**) Dose-dependent reduction of LDL levels from the plasma of transgenic mice (*n* = 4) treated with ss-siRNA **66–70**. (**D**) Dose-dependent reduction of triglyceride levels from the plasma of transgenic mice (*n* = 4) treated with ss-siRNA **66–70**. Data are represented as mean ± SD.

Next, we determined the *in vivo* potency of the ss-siRNA **67–70**. The C_16_ ss-siRNAs **69** and **70** were dosed subcutaneous to human ApoC III transgenic mice (*n* = 4) at 3, 7, 14 and 44 mg/kg twice a week for 3 weeks. The ss-siRNAs **67** and **68** were dosed 100 mg/kg fractionated (25 mg/kg twice a day for 1 day, then wait for 2 days and dose 25 mg/kg twice a day for 1 day) for 3 weeks. The C16-ss-siRNAs **69** (ED_50_ 10 mg/kg per week) and **70** (ED_50_ 20 mg/kg per week) showed remarkable potency in reducing ApoC III mRNA (Figure 16A). Consistent with the *in vitro* potency, the ss-siRNA **69** was more active in reducing ApoC III mRNA than ss-siRNA **70**. The increase in potency observed for the C_16_ ss-siRNAs (**69** and **70**) is more remarkable given that the *in vitro* potency of the ss-siRNAs **69** and **70** was significantly lower (5–15-fold) compared to ss-siRNAs **67** and **68** (Figure 14). The ss-siRNAs **69** (ED_50_ 10 mg/kg per week) and **70** (ED_50_ 10 mg/kg per week) efficiently reduced the plasma ApoC III protein (Figure 16B). Furthermore, the ss-siRNAs **69** (ED_50_ 8 mg/kg per week) and **70** (ED_50_ 15 mg/kg per week) reduced plasma triglycerides and low density lipoprotein (LDL) level in a dose-dependent manner (Figure 16C and D). Finally, throughout the course of the *in vivo* studies the animals were monitored for signs of ss-siRNA-associated toxicities. No adverse effects such as elevated transaminase, bilirubin or increased organ weights were observed.

## DISCUSSIONS

The siRNA therapeutics generally uses duplex RNA for gene silencing using RNAi pathway (1,2). However, it was shown that human Dicer and Ago-2, the enzymes involved in the RNAi pathway, bind short single-stranded RNAs with affinities comparable to siRNAs. This observation suggests that single-stranded RNAs are capable of activating the RNAi pathway (8). We recently demonstrated ss-siRNA activity in mice (9,10). The critical determinants for ss-siRNA activity *in vivo* were a configuration resistant to exonuclease and endonuclease degradation and a metabolically stable 5′-phosphate (9). The 5′-phosphate is important for Ago-2 cleavage activity and binding (12–15). In this study we investigate the effect of chemical modifications on activity, metabolic stability of ss-siRNA and established stereo-electronic requirements for 5′-phosphate of ss-siRNA (12–15).

Previously methyl substitution at the 5′-position of the furanose sugar has been used to change the torsional preference around γ from the *+sc* into the *ap* range in locked nucleic acid (21). The γ of 5′-phosphate of guide strand in the Ago-2 crystal structure (Figure 5A) is also in the *ap* range and we theorized that *R*-5′-Me substitution could pre-organize the conformational preference around γ to mimic this orientation and improve activity relative to the *S*-5′-Me substitution of ss-siRNA. Furthermore, a structural model of the *R*- and *S*-5′-Me groups in the Ago2 binding pocket shows that the bent trajectory of the neighboring phosphodiester linkage could cause the *S*-5′-methyl group to experience tight contacts with the 5′-methylene and one of the non-bridging oxygen atoms of the phosphodiester linkage of the adjacent nucleotide (Figure 5B). ss-siRNA with an *R* -5′-methyl substitution **12** is more active than ss-siRNA **13** with *S*-5′–methyl substitution **13** (Figure 4) consistent with our prediction. Interestingly corresponding siRNAs showed similar potency. These data suggest that, even though ss-siRNA and siRNA use RISC pathway for gene silencing, a differential loading process exists. Our plasma stability study using ss-siRNAs **12** and **13** suggests that 5′-methyl substitution was not capable of stabilizing 5′-phosphate of ss-siRNAs in mice. Nevertheless, this is the first report of activity 5′-(*R*) and 5′-(*S*) methyl-substituted ss-siRNAs and siRNAs.

We also demonstrate that increasing steric bulk or hydrophobicity at 5′-end did not reduce the ss-siRNA or siRNA activity (Figure 7) in cell culture. Interestingly, the introduction of cationic or anionic substitution reduces the potency of ss-siRNA but not siRNA. The reduction of potency was significant for 5′-carboxy ss-siRNA **17** (IC_50_ > 50 nM; Figure 7) relative to 5′-aminomethyl ss-siRNA **16** (IC_50_ 7 nM; Figure 7). Literature suggests that the precise positioning of 5′-phosphate at the phosphate binding pocket of the Ago-2 enzyme is critical for slicer activity (12). In 5′-carboxy ss-siRNA **17**, it is possible to envisage that the charge–charge repulsion between 5′-phosphate and carboxy group may distort the positioning of phosphate in Ago-2 binding pocket and could explain the loss of potency. The 5′-phosphate of the guide strand forms hydrogen bonds and salt bridges with basic amino acids of Ago-2 protein (12) and it is conceivable that 5′-amino group of ss-siRNA **16** may be interfering this interaction and altering the gene silencing activity. Importantly, our results suggest that either steric bulk or cationic or anionic substitution at the 5′-end of ss-siRNA could improve the metabolic stability of 5′-phosphate in mice (Figure 6). These findings suggest that the phosphatase activity on ss-siRNA is very aggressive and 5′-phosphate is rapidly dephosphorylated. Our results provide explanation for the observed lower activity of ss-siRNA relative to siRNA (40).

Phosphonic acid (Figure 2) is not susceptible to the hydrolytic action of phosphatases under physiological conditions (23,24). Our results suggest that ss-siRNA containing 5′-methylenephosphonate substitution was stable in mice. Nevertheless, 5′-methylenephosphonate substitution reduces the potency of ss-siRNA but not siRNA (Figure 8) relative to its corresponding 5′-phosphate analogs. The observed difference in potency could be a result of the conformational and stereo-electronic differences between methylenephosphonate and phosphate. It is known that 5′-phosphate makes critical contacts with several amino acids in the Ago-2 binding pocket (Figure 3) and it is conceivable that due to the difference in stereo-electronics 5′-methylenephophonate ss-siRNA may disrupt these interactions and could contribute to the observed differences in the activity of ss-siRNAs. We addressed the stereo-electronic limitations of 5′-methylenephosphonate modification and designed and synthesized several ss-siRNAs containing 5′-methylenephophonate analogs with different electronic and conformational properties.

Fluorinated methylenephosphonates have suggested to be an isosteric and isopolar analog of phosphate esters (26,27). To our surprise this approach did not provide any enhanced activity to ss-siRNA relative to 5′-methylenephophonate ss-siRNA. Even though mono fluorination provided slight improvement in potency (Figure 9) di-fluorination made it significantly less active. We do not have a clear explanation why ss-siRNA containing 5′-difluoromethylenephosphonate is less active. From these data one could postulate that more than electronic factors special positioning of 5′-phosphate may be critical for RISC loading and subsequent slicer activity of ss-siRNA. Our study with ss-siRNA containing two distinct 5′-methylenephosphonate analogs 5′-*O*-CH_2_-P and 5′-CHP_2_ (Figure 9) further confirms that spatial orientation of 5′-phosphate is critical for ss-siRNA activity. 5′-*O*-CH_2_P analog has an extended oxygen atom and is expected to have different spatial positioning relative to 5′-methylenephosphonate. Our study shows that ss-siRNA containing 5′-*O*-CH_2_P **48** was less active than parent ss-siRNA with 5′-phosphate **3** as well as 5′-methylenephosphonate ss-siRNA **38** (Figure 9). These data clearly suggest that precise positioning of 5′-phosphate is critical for ss-siRNA activity and any perturbation could have significant effect on gene silencing. Similar results were obtained when we altered the charge density and positioning as observed with ss-siRNA containing 5′-bis-methylenephosphonate **50** (Figure 9). Our structural model study of *cis* and *trans* vinylphosphonate nucleoside showed that the *trans* vinylphosphonate can assume a conformation similar to that of the 5′-phosphate in the Ago-2 crystal structure (Figure 11). Our study demonstrates, for the first time, that ss-siRNAs containing *trans* vinylphosphonate and its fluorinated analogs were more potent than corresponding *cis* vinylphosphonates (Figure 13).

Our study clearly establishes the stereo-electronic requirements for 5′-phosphate of ss-siRNA to elicit RNAi. To our knowledge this is the first report where precise dissection of these factors has been examined for ss-siRNA. We identified *trans*-5′-vinylphosphonate as a metabolically stable surrogate phosphate analog for ss-siRNA-mediated gene slicing applications. In addition, we developed a convenient synthetic method to synthesize *trans*-5′-vinylphosphonate containing ss-siRNAs. The ss-siRNA therapeutics has several advantages over double-stranded siRNAs. This approach eliminates the risk that the passenger strand or its metabolites might cause and reduce the cost of manufacture. ss-siRNAs achieve potent *in vivo* inhibition of gene expression using simple saline dosing solutions.

## CONCLUSIONS

In this report we describe the careful structure-based chemical design to identify ss-siRNAs that function as potent inhibitors of gene expression in animals. With the help of known crystal structure of the Ago-2-bound guide strand we designed and synthesized several ss-siRNAs containing 5′-phosphate analogs. Our study demonstrates that phosphatase activity on ss-siRNA was very robust and most successful chemical approach to identify ss-siRNA function in animals was to utilize methylenephosphonate chemistry. We also demonstrate that electronic and spatial orientation of the 5′-phosphate was critical for achieving the best activity of ss-siRNA. Our study identified *trans*-5′-vinylphosphonate as a surrogate phosphate analog for ss-siRNA to activate RNAi in animals. Chemically modified ss-siRNA targeting human apoC III mRNA demonstrated good potency in inhibiting apoC III mRNA and protein in transgenic mice. Moreover, apoC III ss-siRNAs were able to reduce the triglyceride and LDL cholesterol in transgenic mice demonstrating the pharmacological effect of ss-siRNA. ss-siRNAs offer advantages over double-stranded siRNAs such as eliminating the risk that the passenger strand or its metabolites might cause undesirable off-target effects and reduce the cost of manufacture. ss-siRNAs achieve potent *in vivo* inhibition of gene expression using simple saline dosing solutions. This study demonstrates that ss-siRNA provides an alternative strategy for gene silencing and further design optimization will enhance their value as novel therapeutic agents.

## SUPPLEMENTARY DATA

Supplementary Data are available at NAR Online.

SUPPLEMENTARY DATA
