# Identification of metabolically stable 5΄-phosphate analogs that support single-stranded siRNA activity

**DOI:** 10.1093/nar/gkx381

**Published:** 2017-05-01

**Authors:** Thazha P. Prakash, Walt F. Lima, Heather M. Murray, Wenyu Li, Garth A. Kinberger, Alfred E. Chappell, Hans Gaus, Punit P. Seth, Balkrishen Bhat, Stanley T. Crooke, Eric E. Swayze

**Affiliations:** Isis Pharmaceuticals Inc., 2855 Gazelle Ct, Carlsbad, CA 92010, USA

*Nucleic Acids Res* (2015) 43 (6): 2993–3011. doi:10.1093/nar/gkv162

The Authors wish to apologise for an error in Figure 14 of the above article where they have used T instead of U in the sequences. The T at the 5΄-end of the sequence are correct. A new figure is provided below. This correction does not affect the results and conclusion of the article. These errors have also been corrected in the original article.

**Figure 14.**
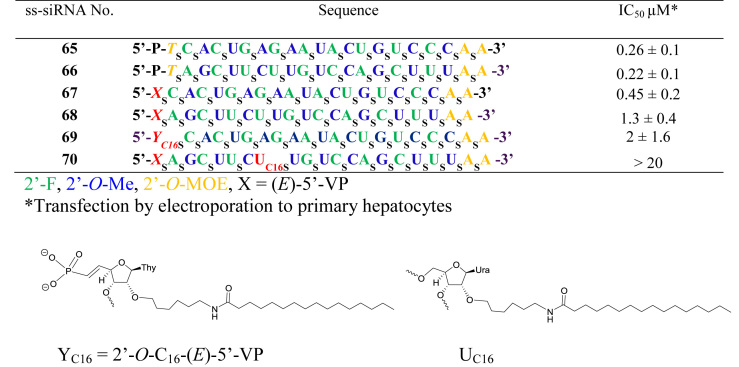
*In vitro* activity of (*E*)-5΄-VP modified ss-siRNA targeting ApoC III mRNA.

