# Supplementary material for: Identification of metabolically stable 5′-phosphate analogs that support single-stranded siRNA activity
Source: Nucleic Acids Res. 2015 Mar 9;43(6):2993–3011. doi: 10.1093/nar/gkv162 (PMC4381071; doi:10.1093/nar/gkv162)
Supplement: SUPPLEMENTARY DATA [file supp_43_6_2993__index.html]

Identification of metabolically stable 5′-phosphate analogs that support single-stranded siRNA activity — Identification of metabolically stable 5′-phosphate analogs that support single-stranded siRNA activity — SUPPLEMENTARY DATA 

# Identification of metabolically stable 5′-phosphate analogs that support single-stranded siRNA activity

## SUPPLEMENTARY DATA

**Files in this Data Supplement:**

- SUPPLEMENTARY DATA
