# Supplementary material for: Identification of metabolically stable 5′-phosphate analogs that support single-stranded siRNA activity
Source: Nucleic Acids Res. 2015 Mar 9;43(6):2993–3011. doi: 10.1093/nar/gkv162 (PMC4381071; doi:10.1093/nar/gkv162)
Supplement: SUPPLEMENTARY DATA [file supp_gkv162_nar-00077-y-2015-File026.pdf]

Supplementary Data

Identification of Metabolically Stable 5'-Phosphate Analogs That Support Single Stranded siRNA Activity

Thazha P. Prakash*, Walt F. Lima, Heather M Murray, Garth A. Kinberger, Wenyu Li, Alfred E. Chappell, Hans Gaus, Punit P. Seth, Balkrishen Bhat, Stanley T. Crooke and Eric E. Swayze

Isis Pharmaceuticals Inc., 2855 Gazelle Ct,, Carlsbad, CA, 92010

* Corresponding author: tprakash@isisph.com

Table of Contents

I	Table S1 analytical data for ss-siRNAs	S3
II	Experimental for synthesis of phosphoramidite **9**	S4-S11
III	Experimental for synthesis of phosphoramidite **11**	S12-S14
IV	Experimental for synthesis of phosphoramidite **30b**	S15-S27
V	Experimental for synthesis of phosphoramidite **31b**	S15-S27
VI	Experimental for synthesis of phosphoramidite **32b**	S15-S28
VII	Experimental for synthesis of phosphoramidite **36**	S28-S34
VIII	Experimental for synthesis of phosphoramidite **39**	S34-S41
IX	Experimental for synthesis of phosphoramidite **43**	S42-S44
X	Experimental for synthesis of phosphoramidite **46**	S44-S49
XI	Experimental for synthesis of phosphoramidite **47**	S50-S51
XII	Experimental for synthesis of phosphoramidite **49**	S52-S58
XIII	Experimental for synthesis of phosphoramidite **53**	S59-S61
XIV	Experimental for synthesis of phosphoramidite **54**	S61-S67
XV	Experimental for synthesis of phosphoramidite **55**	S64-S67
XVI	Experimental for synthesis of phosphoramidite **56**	S68-S69
XVII	Experimental for synthesis of phosphoramidite **57**	S69-S70
XVIII	Experimental for synthesis of phosphoramidite **71**	S71-S77
XIX	Experimental for synthesis of phosphoramidite **72**	S78-S79

Table S1. Analytical data for ss-siRNAs

ss-siRNA No	Chemistry	Calcd Mass	Observed Mass	%UV Purity
**1**	RNA, 5’-P			
**2**	F, **OMe, MOE**, 5’-P	6985.7	6985.0	90
**3**	F, **OMe, MOE**, 5’-P	7186.3	7185.3	98
**12**	F, **OMe, MOE**, 5’- (*R*)-Me-P	7200.0	7199.3	97
**13**	F, **OMe, MOE,** 5’- (*S*)-Me-P	7200.0	7199.6	92
**14**	F, **OMe, MOE**, 5’- (*R*)-MeOCH$_2$-P	7230.3	7229.7	96
**15**	F, **OMe, MOE**, 5’- (*S*)-F- CH$_2$-P	7218.3	7217.6	96
**16**	F, **OMe, MOE**, 5’- (*R*)-NH$_2$ CH$_2$-P	7215.3	7214.5	96
**17**	F, **OMe, MOE**, 5’- (*S*)-Carboxy-P	7230.3	7229.2	94
**37**	F, **OMe, MOE**, 5’-CH$_2$-P-I	7140.2	7139.5	88
**38**	F, **OMe, MOE**, 5’-CH$_2$-P-II	7184.3	7183.6	88
**44**	F, **OMe, MOE**, 5’-CF$_2$-P	7176.3	7175.2	91
**45**	F, **OMe, MOE**, 5’-CHF-P	7202.5	7201.2	90
**48**	F, **OMe, MOE**, 5’-*O*-CH$_2$.P	7200.3	7199.7	95
**50**	F, **OMe, MOE**, 5’-CHP$_2$	7264.3	7263.7	96
**61**	F, **OMe, MOE**, (*E*)-5’-VP	7182.3	7181.4	99
**62**	F, **OMe, MOE**, (*Z*)-5’-VP	7136.3	7135.2	98
**63**	F, **OMe, MOE**, (*E*)-5’-F-VP	7200.6	7199.4	98
**64**	F, **OMe, MOE**, (*Z*)-5’-F-VP	7200.6	7199.2	98
**65**	F, **OMe, MOE**, (*E*)-5’-VP	7277.5	7276.5	98
**66**	F, **OMe, MOE**, (*E*)-5’-VP	7209.3	7208.1	96
**67**	F, **OMe, MOE**, (*E*)-5’-VP	7273.5	7272.0	99
**68**	F, **OMe, MOE**, (*E*)-5’-VP	7205.3	7203.8	99
**69**	F, **OMe, MOE**, (*E*)-5’-VP	7553.0	7551.5	97
**70**	F, **OMe, MOE**, (*E*)-5’-VP	7540.9	7539.4	99

General Experimental. Unless otherwise specified, all reactions were carried out in oven dried glassware under an inert atmosphere of argon gas. Anhydrous solvents and reagents were purchased from commercial vendors and used without any further purification. Yields refer to chromatographically isolated yields. Reactions were monitored by LC MS analysis or thin-layer chromatography (TLC) plates and visualized using UV lamp at 254 nm and developed by a solution of *p*-anisaldehyde (6 mL), H_2SO_4 (8.3 mL), CH_3COOH (2.5 mL) in C_2H_5OH (227 mL) followed by charring. Flash chromatography was performed using silica gel 60 (35-75 μm, EM Science). ^1H and ^{13}C chemical shifts were referenced relative to the signal from residual protons of a lock solvent were referenced and ^{31}P NMR spectra using external standard 85% H_3PO_4. 5'-*O*-DMT-2'-*O*-MOE-5-methyluridine **40** was synthesized using the reported procedure.[1] 5'-*O*-DMT-2'-*O*-(6-aminohexyl)-5-methyluridine **S39** and 5'-*O*-DMT-2'-*O*-(6-aminohexyl)-uridine **S39** were also synthesized using reported procedure.[2]

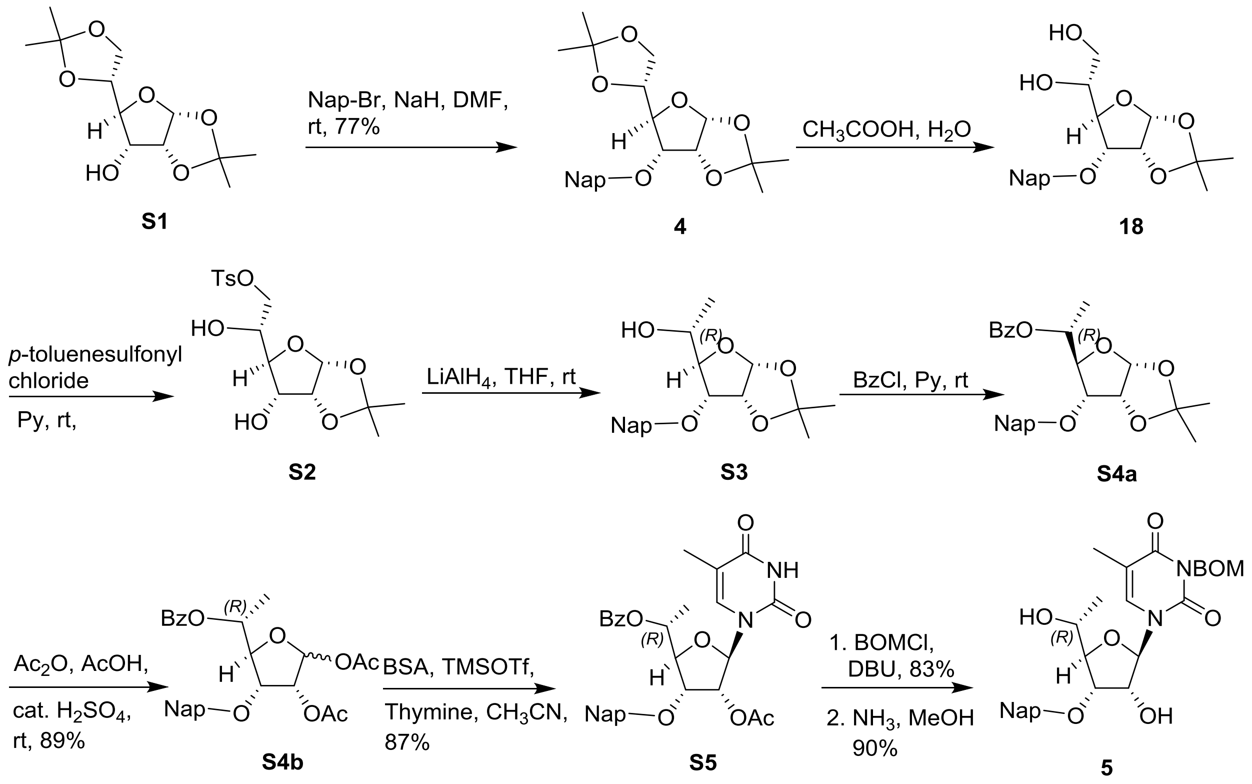

Scheme S1. Synthesis of compound **5**; Nap = 2-(methyl) naphthalene; BOM = benzyloxymethyl

Compound 4. To a suspension of NaH (12.31 g, 307.68 mmol, 60% dispersion in oil) in DMF (300 mL) a solution of 1,2:5,6-Di-*O*-isopropylidene-α-D-allofuranose **S1** (50 g, 192.30 mmol) in DMF (300 mL) was added drop wise. After stirring for 30 min at room temperature 2-(bromo-methyl) naphthalene (Nap-Br, 46.70 g, 211.54 mmol) was added. Stirring continued for additional 2 h. The reaction mixture was poured in to ice water (1000 mL) and extracted with EtOAc (2 x 500 mL). The residue was purified by silica gel column chromatography and eluted with 20-50% EtOAc in hexane to yield 4 (59.36 g, 77%). ^{1}H NMR (300MHz, DMSO-d$_6$): δ 7.97 - 7.84 (m, 4H), 7.58 - 7.42 (m, 3H), 5.74 (d, *J*=3.6 Hz, 1H), 4.86 - 4.76 (m, 2H), 4.73 - 4.60 (m, 1H), 4.24 (dt, *J*=3.2, 7.0 Hz, 1H), 4.01 (dd, *J*=3.2, 8.9 Hz, 1H), 3.95 - 3.68 (m, 3H), 1.47 (s, 3H),

1.31 (s, 3H), 1.26 (d, *J*=1.7 Hz, 5H); LR MS (ESI) calcd for $C_{23}H_{28}O_6Na$ $[M + Na]^+$ m/z = 423.5, found 423.2.

Compound S2.[3] Compound **18** (78.0 g, 216.0 mmol) was dissolved in anhydrous pyridine (500 mL) and cooled in an ice bath. To this p-toluenesulfonyl chloride (49.51 g, 259 mmol) was added and kept in a refrigerator (~ 4 ^{0}C) for 18 h. Reaction was quenched by adding aqueous saturated NH_4Cl solution (10 mL) and stirred for 30 min. Solvent was removed under reduced pressure and residue was dissolved in EtOAc (500 mL) and washed with aqueous saturated $NaHCO_3$ (3 x 500 mL) and brine (500 mL). The organic layer separated and dried (Na_2SO_4), filtered and evaporated under reduced. The residue was purified by silica gel column chromatography and eluted with 20-60% EtOAc in hexane to yield **S2** (71.2 g, 63.7%) . LR MS (ESI) calcd for $C_{27}H_{30}O_8Na$ $[M + Na]^+$ m/z = 537.6, found 537.2.

Compound S3.[3] Compound **S2** (64.0 g, 120.0 mmol) was dissolved in anhydrous THF (650 mL) and cooled in an ice bath under argon atmosphere. To this $LiAlH_4$ (5.42 g, 143 mmol) was added in small portions. Allowed the reaction to come to room temperature and stirring continued for 18 h. The reaction mixture was cooled in an ice bath and 15% aqueous NaOH solution (16.35 mL) and water (32.7 mL) were added drop wise. The reaction mixture was stirred for additional 30 min while cooling in an ice bath. The reaction mixture was filtered through a pad of celite and washed the celite pad with 50% methanol in acetonitrile (1500 mL). Combined filtrate and washing and concentrated under reduced pressure. Residue obtained was purified by silica gel column chromatography and eluted with 80-100 % dichloromethane in EtOAc to yield **S3** (34.17 g, 79.8%) . LR MS (ESI) calcd for $C_{20}H_{24}O_5Na$ $[M + Na]^+$ m/z = 367.4, found 367.1.

Compound S4a.[3]To a solution of compound **S3** (33.0 g, 96.0 mmol) in anhydrous pyridine (480 mL) benzoyl chloride (12.22 mL, 105.0 mmol) was added. The reaction mixture was stirred at room temperature for 4 h under argon atmosphere. Solvent was removed under reduced pressure and residue was dissolved in EtOAc (500 mL) and washed with water (500 mL) and brine (500 mL). The organic layer separated and dried (Na_2SO_4), filtered and evaporated under reduced. The residue obtained was purified buy silica gel column chromatography and eluted with 40-100 % dichloromethane in EtOAc to yield **S4a** (26.77 g, 63.2%) .   LR MS (ESI) calcd for $C_{27}H_{28}O_6Na$ $[M + Na]^+$ *m/z* = 471.5, found 471.1.

Compound S4b.[3] Dried Compound **S4a** (21.1 g, 47.04 mmol) was dissolved in a mixture of glacial acetic acid (104 mL) and acetic anhydride (17.2 mL). To this solution was added 14 drops of concentrated H_2SO_4. After 1.5 h, the resulting light brown solution was diluted in EtOAc (600 mL), washed with sat. $NaHCO_3$ (5 x 600 mL), dried over anhydrous Na_2SO_4, filtered, evaporated and dried under high vacuum to yield compound **S4b** (22.7 g, 99%) as a pale oil. ^1H NMR (300MHz, $CDCl_3$): δ 8.07 - 7.92 (m, 2H), 7.89 - 7.30 (m, 10H), 6.15 (s, 1H), 5.50 - 5.36 (m, 2H), 4.97 - 4.71 (m, 2H), 4.60 (d, *J*=10.9 Hz, 1H), 4.55 - 4.38 (m, 1H), 4.26 (dd, *J*=4.5, 7.7 Hz, 1H), 2.15 (s, 3H), 2.05 (s, 3H), 1.75 (s, 3H); HR MS (ESI) calcd for $C_{28}H_{28}O_8Na$ $[M + Na]^+$ *m/z* = 515.1654, found 515.1648.

Compound S5. A mixture of compound **S4b** (23.3 g, 46.70 mmol) and thymine (10.01 g, 79.40 mmol) was suspended in anhydrous CH_3CN (233 mL). To this mixture was added *N*,*O*-bis-trimethylsilylacetamide (41.06 mL, 167.94 mmol), followed by heating at 55 ºC for 1 h. The mixture was cooled to 0 ºC, then trimethylsilyl trifluoromethanesulfonate (19.07 mL, 105.54 mmol) was added dropwise over 15 min. The mixture was subsequently heated at 55 ºC. After 3 h the mixture was cooled to 0 ºC and quenched with the dropwise addition of saturated aqueous

$NaHCO_3$ (20 mL). The mixture was poured into EtOAc, washed with brine (4 x 0.8 mL), dried over anhydrous Na_2SO_4, filtered, evaporated and dried under high vacuum. The residue was purified by silica gel column chromatography and eluted with 20% to 50% EtOAc in hexanes to yield Compound **S5** (22.27 g, 85%) as white foam. ^{1}H NMR (300MHz, $CDCl_3$): δ 8.50 (br. s., 1H), 7.99 (dd, *J*=1.2, 8.2 Hz, 2H), 7.91 - 7.70 (m, 5H), 7.65 - 7.33 (m, 6H), 6.12 (d, *J*=5.5 Hz, 1H), 5.61 - 5.43 (m, 1H), 5.43 - 5.28 (m, 1H), 4.93 - 4.64 (m, 2H), 4.62 - 4.42 (m, 1H), 4.37 - 4.23 (m, 1H), 2.31 - 2.12 (m, 3H), 2.05 (s, 2H), 1.37 (d, *J*=3.2 Hz, 3H); ^{13}C NMR (75MHz, $CDCl_3$): δ 170.2, 165.4, 163.3, 150.2, 134.8, 134.3, 133.5, 133.2, 133.1, 129.6, 129.5, 128.7, 128.4, 127.9, 127.7, 127.0, 126.3, 126.2, 125.7, 111.7, 87.0, 84.3, 75.2, 73.8, 73.2, 70.2, 20.7, 16.2, 11.7; HR MS (ESI) calcd for $C_{31}H_{31}N_2O_8$ $[M + H]^+$ *m/z* = 559.2052, found 559.2054.

Compound 5. Compound **S3** (11.71 g, 20.98 mmol) was dissolved in anhydrous DMF (115 mL). To this was added 1,8-diazabicycl-[5-4-0] undec-7-ene (DBU, 9.30 mL, 62.41 mmol). The reaction mixture was cooled in an ice bath. To this was added benzyl chloromethyl ether (4.36 mL, 31.47 mmol), and stirred at 0 ºC for 1 h. The mixture was diluted with EtOAc (200 mL), washed with saturated aqueous $NaHCO_3$ (200 mL) and brine (200 mL) then dried (Na_2SO_4), filtered and evaporated. The residue obtained was dissolved in methanol (89 mL) and K_2CO_3 (8.76 g, 63.40 mmol) was added. The reaction mixture was stirred at room temperature for 1 h. The mixture was poured into EtOAc (200 mL), washed with water (200 mL) and brine (200 mL), dried over anhydrous Na_2SO_4, filtered and evaporated. The residue was purified by silica gel column chromatography and eluted with 5% methanol in CH_2Cl_2 to yield Compound **5** (8.93 g, 80%) as a white foam. ^{1}H NMR (300MHz, DMSO-d_6): δ 8.00 - 7.77 (m, 4H), 7.65 - 7.44 (m, 4H), 7.40 - 7.17 (m, 5H), 5.95 (d, *J*=7.0 Hz, 1H), 5.55 (d, *J*=6.0 Hz,1H), 5.46 - 5.26 (m, 2H), 5.18 (d, *J*=4.9 Hz, 1H), 4.96 - 4.72 (m, 2H), 4.66 - 4.56 (m, 2H), 4.35 - 4.19 (m, 1H), 4.11 -

3.96 (m, 1H), 3.95 - 3.75 (m, 2H), 1.84 (s, 3H), 1.07 (d, *J*=9.0 Hz 3H); LR MS (ESI) calcd for $C_{30}H_{33}N_2O_7$ $[M + H]^+$ *m/z* = 533.2256, found 533.2259.

Compound 6. Compound **5** (4.30 g, 8.07 mmol) was dried over P_2O_5 under reduced pressure and dissolved in anhydrous DMF (24 mL). The mixture was cooled to -20 ^oC. To this was added NaH (0.48 g, 12.11 mmol, 60% dispersion in mineral oil) with stirring for 30 minutes followed by addition of 1-methoxy-2-iodoethane (2.25 g, 12.11 mmol). The reaction mixture was warmed up to 0 ^oC. After stirring for 1.5 h at 0 ^oC the reaction mixture was cooled to -20 ^oC and additional NaH (0.48 g, 12.11 mmol, 60% dispersion in mineral oil) was added. Stirring was continued at -20 ^oC for 30 minutes and 1-methoxy-2-iodoethane (2.25 g, 12.11 mmol) was added. The reaction mixture was warmed to 0 ^oC and with stirring for an additional 1.5 h. The reaction was quenched with methanol (5 mL), diluted with EtOAc (100 mL), washed with water (100 mL) and brine (100 mL), dried over Na_2SO_4, filtered and evaporated under reduced pressure. The residue was purified by silica gel column chromatography and eluted with 5% methanol in CH_2Cl_2 to yield compound **6** (2.95 g, 62%). ^1H NMR (300MHz, DMSO-d_6): δ 8.07 - 7.82 (m, 4H), 7.60 - 7.44 (m, 4H), 7.40 - 7.16 (m, 5H), 5.98 (d, *J*=5.3 Hz, 1H), 5.35 (s, 2H), 5.28 (d, *J*=4.9 Hz, 1H), 4.78 (s, 2H), 4.59 (s, 2H), 4.28 - 4.12 (m, 2H), 4.02 - 3.87 (m, 2H), 3.68 (dd, *J*=3.3, 5.9 Hz, 2H), 3.51 - 3.38 (m, 2H), 3.19 (s, 3H), 1.84 (s, 3H), 1.12 (d, *J*=6.0 Hz, 3H); ^{13}C NMR (75MHz, $CDCl_3$): δ 163.3, 151.0, 137.9, 135.0, 133.2, 133.0, 128.5, 128.2, 127.8, 127.7, 127.6, 126.7, 126.2, 126.0, 125.8, 125.6, 110.1, 93.0, 87.2, 79.9, 74.3, 72.3, 72.2, 72.1, 72.0, 70.5, 70.1, 67.2, 59.0, 18.6, 13.0; HR MS (ESI) calcd for $C_{33}H_{39}N_2O_8$ $[M + H]^+$ *m/z* = 591.2670, found 591.2672.

Compound 7. Compound **6** (2.2 g, 3.73 mmol) was dissolved in anhydrous pyridine (7 mL) and cooled in an ice bath. To this benzoyl chloride (0.88 mL, 7.61 mmol) was added and once the

addition was over, reaction mixture was allowed to come to room temperature. The reaction mixture was stirred at room temperature for 4 h under an argon atmosphere and subsequently cooled the reaction mixture in an ice bath and quenched by adding saturated aqueous $NaHCO_3$ (5 mL). Diluted the reaction mixture with EtOAc (50 mL) and washed with saturated aqueous $NaHCO_3$ (2 x 50 mL), brine (50 mL), dried over Na_2SO_4, filtered and concentrated. The residue obtained was dissolved in CH_2Cl_2 (40 mL) and added 2,4-dichloro-5,6-dicyano-1,4-benzoquinone (DDQ, 1.93 g, 8.5 mmol) and H_2O (0.15 mL, 8.5 mmol) and stirred at room temperature. After 18 h, diluted the reaction mixture with EtOAc (60 mL), washed with saturated aqueous $NaHCO_3$ (2 x 80 mL), brine (50 mL), dried over Na_2SO_4, filtered and evaporated under reduced pressure. The residue was dissolved in MeOH (30 mL) and palladium hydroxide (1.1 g, 20 wt% Pd on carbon dry base) and stirred under H_2 atmosphere for 6 h. To this acetic acid (0.56 mL) was added and stirred for 5 min. The reaction mixture was filtered through a pad of celite 545, and washed the celite with copious amount of MeOH. The combined filtrate and washing were concentrated under reduced pressure and the residue was purified by silica gel column chromatography and eluted with 5% methanol in CH_2Cl_2 to yield compound **7** (1.43 g, 88%). ^{1}H NMR (300MHz, DMSO-d$_6$): δ 11.37 (s, 1H), 8.15 - 7.94 (m, 2H), 7.70 (d, *J*=7.5 Hz, 1H), 7.64 - 7.48 (m, 2H), 7.06 (s, 1H), 5.86 (d, *J*=6.2 Hz, 1H), 5.39 - 5.17 (m, 2H), 4.42 (d, *J*=4.1 Hz, 1H), 4.13 - 3.89 (m, 2H), 3.70 (td, *J*=4.7, 13.8 Hz, 2H), 3.54 - 3.40 (m, 2H), 3.31 (s, 3H), 1.36 (d, *J*=6.4 Hz, 3H), 1.29 (s, 3H); ^{13}C NMR (75MHz, $CDCl_3$): δ 166.5, 163.3, 150.1, 134.8, 133.5, 129.9, 129.5, 128.8, 111.1, 87.4, 85.4, 82.5, 71.8, 70.3, 68.5, 59.0, 16.1, 11.6; HR MS (ESI) calcd for $[M + H]^+$ *m/z* = $C_{21}H_{27}N_2O_8$ 435.1746, found 435.1748.

Compound 8. A mixture of compound **7** (1.33 g, 3.06 mmol) and imidazole (2.09, 30.70 mmol) was dissolved in anhydrous DMF (11.4 mL). To this solution *tert*-butyldimethylsilyl chloride

(TBDMSCl, 2.31 g, 15.33 mmol) was added with stirring at room temperature for 16 h under an atmosphere of argon. The reaction mixture was diluted with EtOAc (75 mL) and washed with saturated aqueous $NaHCO_3$ (2 x 60 mL) and brine (50 mL), dried over Na_2SO_4, filtered and concentrated. The residue obtained was dissolved in methanolic ammonia (20 mL, 7M) and stirred for 24 h at 55 ^oC. The solvent was removed under reduced pressure and the residue was purified by silica gel column chromatography and eluted with 50% EtOAc in hexanes to yield compound **8** (1.21 g, 89%). ^1H NMR (300MHz, DMSO-d_6): δ 11.35 (s, 1H), 7.75 (d, *J*=1.1 Hz, 1H), 5.86 (d, *J*=7.2 Hz, 1H), 5.19 (d, *J*=4.7 Hz, 1H), 4.37 (dd, *J*=1.7, 4.7 Hz, 1H), 4.05 (dd, *J*=4.8, 7.1 Hz, 1H), 3.86 - 3.72 (m, 1H), 3.86 - 3.71 (m, 1H), 3.64 (dd, *J*=1.8, 4.1 Hz, 1H), 3.58 - 3.50 (m, 2H), 3.42 - 3.34 (m, 2H), 3.17 (s, 3H), 1.79 (d, *J*=0.9 Hz, 3H), 1.11 (d, *J*=6.6 Hz, 3H), 0.88 (s, 9H), 0.15 - 0.02 (m, 6H); ^{13}C NMR (75MHz, $CDCl_3$): δ 163.6, 150.4, 140.0, 110.8, 93.2, 90.9, 79.5, 71.9, 69.9, 69.2, 67.4, 58.9, 25.7, 18.7, 18.1, 12.3, -4.5, -4.9; LR MS (ESI) calcd for $C_{20}H_{36}N_2NaO_7Si$ $[M + H]^+$ *m/z* = 445,2346, found 445,2350.

Compound 9. Compound **8** (0.42 g, 0.96 mmol) was mixed with 4,4'-dimethoxytrityl chloride (0.82 g, 2.41 mmol) and dried over P_2O_5 under reduced pressure. The mixture was dissolved in anhydrous pyridine (3 mL) and 2,6-lutidine (0.27 mL, 2.4 mmol) stirred at 45 ^oC for 18 h under an atmosphere of argon. The reaction mixture was cooled to room temperature and diluted with EtOAc (40 mL) and washed with saturated aqueous $NaHCO_3$ (60 mL) and brine (40 mL), dried over Na_2SO_4, filtered and concentrated. The residue obtained was purified by silica gel column chromatography and eluted first with 50% EtOAc in hexanes and then with 5% methanol in CH_2Cl_2. The product obtained was dissolved in a mixture of triethylamine trihydrofluoride (1.38 mL, 8.44 mmol) and triethylamine (0.58 mL, 4.22 mmol) in THF (8.4 mL). After 72 h the mixture was diluted with EtOAc (60 mL), washed with water (40 mL), saturated aqueous

$NaHCO_3$ (40 mL) and brine (40 mL) then dried over Na_2SO_4, filtered and evaporated. The residue obtained was purified by silica gel column chromatography and eluted with 70% EtOAc in hexanes to yield 5'-DMT-(*R*)-5'-methyl-2'-*O*-(2-methoxyethyl)thymidine (0.44 g, 73%). ^{1}H NMR (300MHz, DMSO-d_6): δ 11.38 (s, 1H), 7.52 - 7.42 (m, 2H), 7.41 - 7.16 (m, 7H), 7.08 (d, *J*=0.9 Hz, 1H), 6.89 (dd, *J*=2.9, 8.9 Hz, 4H), 5.79 (d, *J*=7.0 Hz, 1H), 5.06 (d, *J*=5.8 Hz, 1H), 4.42 (d, *J*=3.4 Hz, 1H), 3.97 (t, *J*=6.2 Hz, 1H), 3.79 - 3.72 (m, 7H), 3.72 - 3.57 (m, 3H), 3.51 - 3.43 (m, 2H), 3.37 (dd, *J*=3.0, 6.4 Hz, 1H), 3.23 (s, 3H), 1.37 (s, 3H), 0.78 (d, *J*=6.4 Hz, 3H); ^{13}C NMR (75MHz, $CDCl_3$): δ 163.5, 158.6, 150.3, 146.3, 136.8, 136.4, 135.6, 130.3, 130.2, 128.1, 127.8,126.9, 113.1, 111.0, 87.7, 86.7, 86.2, 82.0, 71.9, 70.2, 69.9, 68.4, 59.0, 55.2, 17.9, 11.7; LR MS (ESI) calcd for $C_{35}H_{39}N_2O_9$ $[M - H]^-$ *m*/*z* = 631.7, found 631.2. 5'-DMT-(*R*)-5'-methyl-2'-*O*-(2-methoxyethyl)thymidine thus obtained (0.35 g, 0.55 mmol) was dried over P_2O_5 under reduced pressure then dissolved in anhydrous DMF (1.8 mL). To this 1-H-tetrazole (0.033 g, 0.48 mmol), *N*-methylimidazole (0.012 mL, 0.15 mmol) and 2-cyanoethyl-*N*,*N*,*N*',*N*'-tetraisopropylphosphordiamidite (0.27 mL, 0.86 mmol) were added. After 3 h, EtOAc (40 mL) was added and the mixture was washed with saturated $NaHCO_3$ (30 mL) and brine (40 mL), dried over anhydrous Na_2SO_4, filtered and evaporated *in vacuo* to give an oil. The oily residue was purified by silica gel column chromatography by eluting with EtOAc/hexane (1:1) to yield compound **9** (0.38 g, 83%) as a white foam. ^{31}P NMR (121 MHz, $CDCl_3$): δ 150.2, 149; HR MS (ESI) calcd for $C_{44}H_{56}N_4O_{10}P$ $[M + H]^+$ *m*/*z* = 831.4893, found 831.4895.

Compound 10. Compound **8** (0.57 g, 1.28 mmol) and *p*-nitrobenzoic acid (*p*-NO_2-BzOH, 1.02 g, 5.03 mmol) was dried together under reduced pressure over P_2O_5. The mixture was dissolved in anhydrous THF (12.9 mL). To this triphenylphosphine (Ph_3P, 1.33 g, 5.07 mmol) was added. To this diisopropylazodicarboxylate (DIAD, 0.97 mL, 5.07 mmol) was added drop wise. The

reaction mixture was stirred at room temperature for 2 h under argon atmosphere. After that, the reaction mixture was concentrated under reduced pressure and residue dissolved in EtOAc (100 mL), washed with aqueous $NaHCO_3$ (100 mL) and brine (100 mL), dried over Na_2SO_4, filtered and evaporated under reduced pressure. The residue was purified by silica gel column chromatography and eluted with 50% EtOAc in hexane to yield (*S*)-5'-methyl-5'-*O*-p-nitrobenzoyl-3'-O-(*tert*-butyldimethylsilyl)-2'-*O*-(2-methoxyethyl)thymidine (0.68 g, 89%). HR MS (ESI) calcd for $C_{27}H_{40}N_3O_{10}Si$ $[M + H]^+$ m/z = 594.2454, found 594.2451. Dissolved in methanolic ammonia (7 M, 10 mL) in a pressure bottler and heated at 55 ^oC for 12 h. The solvent was removed under reduced pressure and the residue obtained was purified by silica gel column chromatography and eluted with 50% EtOAc in hexane to yield compound **10** (0.42 g, 86%). ^1H NMR (300MHz, DMSO-d_6): δ 11.32 (s, 1H), 7.92 (d, *J*=1.1 Hz, 1H), 5.94 - 5.75 (m, 1H), 5.16 (d, *J*=4.3 Hz, 1H), 4.26 (t, *J*=4.4 Hz, 1H), 3.97 (t, *J*=5.0 Hz, 1H), 3.86 - 3.73 (m, 1H), 3.68 (dd, *J*=2.1, 4.0 Hz, 1H), 3.47 - 3.35 (m, 2H), 3.19 (s, 3H), 1.77 (d, *J*=0.8 Hz, 3H), 1.16 (d, *J*=6.6 Hz, 3H), 0.88 (s, 9H), 0.09 (s, 6H); ^{13}C NMR (75MHz, $CDCl_3$): δ 163.9, 150.3, 138.9, 110.6, 92.2, 88.8, 80.5, 72.0, 71.3, 69.9, 66.7, 58.9, 25.7, 20.3, 18.1, 12.35, -4.7, -4.9; LR MS (ESI) calcd for $C_{20}H_{37}N_2O_7Si$ $[M + H]^+$ m/z = 445.6, found 445.2.

Compound 11. Compound **10** (0.34 g, 0.77 mmol) was mixed with 4,4'-dimethoxytrityl chloride (0.65 g, 1.93 mmol) and dried over P_2O_5 under reduced pressure. The mixture was dissolved in anhydrous pyridine (2.4 mL) and 2,6-lutidine (0.22 mL,1.93) was added and stirred at 45 ^oC for 18 h under an atmosphere of argon. The reaction mixture was cooled to room temperature and diluted with EtOAc (40 mL) and washed with saturated aqueous $NaHCO_3$ (60 mL) and brine (40 mL), dried over Na_2SO_4, filtered and concentrated. The residue obtained was purified by silica gel column chromatography and eluted first with 50% EtOAc in hexanes and

then with 5% methanol in CH_2Cl_2 to yield (*S*)-5'-methyl-5'-*O*-4,4'-dimethoxytrityl-3'-O-(*tert*-butyldimethylsilyl)-2'-*O*-(2-methoxyethyl)thymidine (0.52 g, 90%). ^{1}H NMR (300MHz, DMSO-d_6): δ 11.41 (s, 1H), 7.57 (s, 1H), 7.44 (d, *J*=7.9 Hz, 2H), 7.38 - 7.13 (m, 7H), 6.88 (dd, *J*=6.2, 8.5 Hz, 4H), 5.80 (d, *J*=5.7 Hz, 1H), 4.94 (d, *J*=6.2 Hz, 1H), 4.26 - 4.08 (m, 1H), 4.01 (t, *J*=5.6 Hz, 1H), 3.78 - 3.69 (m, 7H), 3.69 - 3.57 (m, 2H), 3.57 - 3.47 (m, 1H), 3.47 - 3.39 (m, 2H), 3.21 (d, *J*=0.9 Hz, 3H), 1.65 (s, 3H), 0.72 (d, *J*=6.2 Hz, 3H); ^{13}C NMR (75MHz, $CDCl_3$): δ 163.7, 158.7, 150.2, 146.1, 136.6, 136.3, 135.6, 130.5, 130.4, 128.3, 127.7, 126.9, 113.0, 110.9, 88.1, 87.2, 87.0, 82.8, 71.7, 70.2, 69.5, 69.1, 58.9, 55.2, 18.5, 12.3; HR MS (ESI) calcd for $C_{41}H_{53}N_2O_9Si$ [M - H]$^-$ *m/z* = 745.3456, found 745.3458. The product obtained was dissolved in a mixture of triethylamine trihydrofluoride (0.54 mL, 3.34 mmol) and triethylamine (0.23 mL, 1.67 mmol) in THF (7.6 mL). After 18 h the mixture was diluted with EtOAc (60 mL), washed with water (40 mL), saturated aqueous $NaHCO_3$ (40 mL) and brine (40 mL) then dried over Na_2SO_4, filtered and evaporated. The residue obtained was purified by silica gel column chromatography and eluted with 70% EtOAc in hexanes to yield (*S*)-5'-methyl-5'-*O*-4,4'-dimethoxytrityl-2'-*O*-(2-methoxyethyl)thymidine (0.39 g, 92%). ES MS *m/z* 631.2 [M - H]$^-$. 5'-DMT-(*S*)-5'-methyl-2'-O-(2-methoxyethyl)thymidine thus obtained (0.37 g, 0.58 mmol) was dried over P_2O_5 under reduced pressure then dissolved in anhydrous DMF (2.5 mL). To this 1-H-tetrazole (0.036 g, 0.51 mmol), *N*-methylimidazole (14 µL, 0.18 mmol) and 2-cyanoethyl-*N*,*N*,*N*',*N*'-tetraisopropylphosphordiamidite (0.55 mL, 1.74 mmol) were added. After 3 h, EtOAc (50 mL) was added and the mixture was washed with saturated $NaHCO_3$ (40 mL) and brine (50 mL), dried over anhydrous Na_2SO_4, filtered and evaporated *in vacuo* to give an oil. The oily residue was purified by silica gel column chromatography by eluting with EtOAc/hexane (1:1) to yield compound **11** (0.41 g, 86%) as a white foam. ^{31}P NMR (121MHz,

$CDCl_3$): δ 150.19, 150.04; LR MS (ESI) calcd for $C_{44}H_{56}N_4O_{10}P$ $[M - H]^-$ *m/z* = 831.5, found 831.4.

Compound 18. Compound **4** (200.0 g, 500 mmole) was added in small portions to a solution of acetic acid (2200 mL) and water (740 mL). The reaction mixture was stirred at room temperature for 16 h after which, TLC analysis (30% EtOAc/hexanes) indicated complete consumption of compound **4**. The reaction mixture was then concentrated under reduced pressure until most of the acetic acid was removed. The remaining solution was poured into a stirred mixture of EtOAc (1000 mL) and water (1000 mL). Solid KOH was then added to the above mixture until the aqueous layer was strongly basic (pH>12). The organic layer was then separated, washed with saturated sodium bicarbonate solution and brine then dried (Na_2SO_4), filtered and concentrated under reduced pressure to provide **18** (172 g, 95%) as a yellow foam, which was used without any further purification. ^{1}H NMR (300MHz, DMSO-d_6): δ 7.97 - 7.81 (m, 4H), 7.59 - 7.43 (m, 3H), 5.72 (d, *J*=3.8 Hz, 1H), 4.89 - 4.61 (m, 4H), 4.52 (t, *J*=5.6 Hz, 1H), 4.07 - 3.92 (m, 2H), 3.71 (d, *J*=5.5 Hz, 1H), 3.53 - 3.33 (m, 2H), 1.47 (s, 3H), 1.30 (s, 3H); ^{13}C NMR (75MHz, $CDCl_3$) δ 134.3, 133.1, 128.4, 127.9, 127.7, 127.2, 125.9, 113.2, 104.1, 79.0, 77.3, 72.3, 71.0, 63.0, 26.7, 26.5; HR MS (ESI) calcd for $C_{20}H_{23}O_6$ $[M - H]^-$ *m/z* = 359.1464, found 359.1461.

Compound 19. Compound **18** (50.0 g, 138 mmoles) was co-evaporated with pyridine (2 x 150 mL). Residue dissolved in anhydrous pyridine 517 mL) and *tert*-butylchlorodiphenylsilane (TBDPS-Cl, 41.72 g, 151.75 mmol) was added. The reaction was stirred at room temperature for 16 h under argon atmosphere after which, TLC analysis (30% EtOAc/hexanes) indicated complete consumption of compound **18**. The reaction mixture was then concentrated under reduced pressure until most of the solvent was removed. The residue was dissolved in EtOAc

(600 mL) and washed with saturated $NaHCO_3$ (600 mL) and brine (500 mL), dried over anhydrous Na_2SO_4, filtered and evaporated under reduced pressure. The oily residue was purified by silica gel column chromatography and eluted with 10-30% EtOAc in hexane to yield 6-*tert*-butyldimethylsilyl derivative of compound **18** (69.79 g, 84%). *tert*-Butyldimethylsilyl derivative of compound **18** (16.7 g, 27.9 mmol) was dried over P_2O_5 under reduced pressure over night and dissolved in anhydrous DMF (110 mL). Cooled the reaction mixture in an ice bath and sodium hydride (60% dispersion is mineral oil, 1.2 g, 27.9 mmol) was added under argon atmosphere. The reaction mixture was stirred at room temperature for 30 min. To this benzyl bromide (4 mL, 23.48 mmol) was added and the reaction mixture was allowed to come to room temperature. After 8 h quenched the reaction by adding methanol (10 mL) and then diluted with EtOAc (200 mL). The organic phase was washed with aqueous saturated sodium bicarbonate solution (200 mL), brine (200 mL), dried (Na_2SO_4), filtered and concentrated under reduced pressure. The residue obtained was purified by silica gel column chromatography and eluted with 30% EtOAc in hexane to yield **19** (15.64 g, 81%). ^{1}H NMR (300MHz, DMSO-d_6): δ 7.95 - 7.83 (m, 1H), 7.80 - 7.55 (m, 8H), 7.54 - 7.24 (m, 14H), 5.74 (d, *J*=3.8 Hz, 1H), 4.78 (s, 2H), 4.64 (s, 2H), 4.59 - 4.50 (m, 1H), 4.24 - 4.16 (m, 1H), 4.05 - 3.96 (m, 1H), 3.92 - 3.83 (m, 1H), 3.83 - 3.70 (m, 2H), 1.46 (s, 3H), 1.31 (s, 3H), 0.95 (s, 9H); ^{13}C NMR (75MHz, $CDCl_3$): δ 139.0, 135.6, 135.1, 133.5, 133.4, 133.1, 133.0, 129.7, 129.6, 128.2, 128.1, 127.9, 127.7, 127.6, 127.6, 127.4, 127.2, 126.8, 126.7, 126.0, 125.9, 112.9, 104.1, 79.4, 79.1, 77.9, 77.1, 73.9, 72.1, 63.8, 26.9, 26.8, 26.8, 26.7, 19.1; HR MS (ESI) calcd for $C_{43}H_{48}O_6SiNa$ $[M + Na]^+$ *m/z* = 711.3073, found 711.3073.

Compound 20. Compound **19** (19.28 g, 27.98 mmol) was dissolved in a mixture of glacial acetic acid (58 mL) and acetic anhydride (11.6 mL). To this catalytic amount of concentrated

sulfuric acid (0.2 mL) was added. After stirring for 3 h at room temperature solvent was removed under reduced pressure and the residual oil was diluted with EtOAc (300 mL). The organic layer was washed with water (300 mL) and saturated aqueous sodium bicarbonate solution (until pH<10), brine, dried (Na_2SO_4), filtered and concentrated. Purification by silica gel column chromatography (20-30% EtOAc in hexane) provided anomeric mixture of diacetates **20** (20.12 g, 98%). HR MS (ESI) calcd for $C_{44}H_{48}O_8SiNa$ $[M + Na]^+$ m/z = 755.2964, found 755.2964.

Compound 21. To a stirred solution of compound **20** (19.74 g, 26.96 mmol) and thymine (7.66 g, 60.70 mmol) in anhydrous acetonitrile (164 mL) *N*,*O*-bis(trimethylsilyl)acetamide (49.60 mL) was added. After heating at 67 ^oC for 2 h under argon atmosphere, the reaction mixture was cooled in an ice bath. To this trimethylsilyltrifluoromethanesulfonate (11.20 mL, 61.92 mmol) was added drop wise. The reaction mixture was heated at 65 ^oC for 2 h under argon atmosphere. The reaction was quenched with an ice cold aqueous saturated $NaHCO_3$ solution (400 mL). The reaction mixture was extracted with EtOAc (2 x 200 mL) and the organic phase was washed with brine (400 mL), dried (Na_2SO_4), filtered and concentrated under reduced pressure. The residue thus obtained was purified by silica gel column chromatography and eluted with 50% EtO Ac in hexane to yield **21** (14.74 g, 67%). ^1H NMR (300MHz, DMSO-d_6): δ 11.43 (s, 1H), 8.02 - 7.69 (m, 3H), 7.67 - 7.26 (m, 20H), 6.00 (d, *J*=5.7 Hz, 1H), 5.45 (t, *J*=5.7 Hz, 1H), 4.81 - 4.70 (m, 1H), 4.69 - 4.57 (m, 3H), 4.56 - 4.46 (m, 1H), 4.26 (t, *J*=4.3 Hz, 1H), 3.96 (d, *J*=4.5 Hz, 1H), 3.90 - 3.75 (m, 2H), 2.08 (s, 3H), 1.52 (d, *J*=0.8 Hz, 3H), 0.97 (s, 9H); ^{13}C NMR (75MHz, $CDCl_3$): δ 170.1, 163.3, 150.3, 137.8, 135.6, 135.5, 135.1, 134.6, 133.1, 133.0, 132.9,132.8, 129.9, 128.5, 128.2,127.9, 127.8, 126.6, 126.2, 125.6, 111.6, 85.9, 82.9, 79.6, 74.9, 74.1, 72.6, 62.6, 26.8, 20.7, 19.1, 11.8; LR MS (ESI) calcd for $C_{47}H_{50}N_2O_8SiNa$ $[M + Na]^+$ m/z = 821.3, found 821.2.

Compound 22. Compound **22** (10.02 g, 78%) was synthesized form compound **21** (12.02 g, 14.66 mmol), DMF (80 mL), DBU (6.52 mL, 43.60 mmol), benzyl chloromethyl ether (2.88 mL, 31.47 mmol), methanolic ammonia (124 mL) and 1,4-dioxane (60 mL) using the same procedure used for the synthesis of compound **5**. ^{1}H NMR (300MHz, DMSO-d$_6$): δ 8.50 - 8.11 (m, 7H), 8.11 - 7.77 (m, 21H), 6.49 (d, *J*=5.1 Hz, 1H), 5.94 (d, *J*=1.8 Hz, 2H), 5.47 - 5.22 (m, 4H), 5.21 - 5.03 (m, 3H), 4.98 - 4.71 (m, 3H), 4.51 - 4.29 (m, 3H), 4.16 (s, 1H), 2.17 - 1.94 (m, 3H), 1.70 - 1.51 (m, 9H); ^{13}C NMR (75MHz, CDCl$_3$): δ 163.2, 151.6, 138.0, 137.7, 135.56, 135.5, 134.1, 133.1, 132.8, 130.0, 128.5, 128.3, 128.2, 127.9, 127.8, 127.7, 127.6, 127.5, 126.8, 126.6, 126.3, 126.2, 125.5, 110.7, 88.8, 82.3, 79.8, 75.9, 73.5, 72.5, 72.1, 72.0, 70.6, 62.4, 26.9, 19.1, 12.6; HR MS (ESI) calcd for $C_{53}H_{57}N_2O_8Si$ $[M + H]^+$ *m/z* = 877.3810, found 877.3810.

Compound 23. Compound **22** (17.58 g, 20.07 mmol) was dried over P_2O_5 under reduced pressure and dissolved in anhydrous DMF (100 mL). The mixture was cooled to -10 ^{o}C. To this NaH (2.41 g, 60.21 mmol, 60% dispersion in mineral oil) was added and stirred for 30 min. To this 1-methoxy-2-iodoethane (13.07 g, 70.25 mmol) was added. The reaction mixture was warmed up to 0 ^{o}C. After stirring for 1.5 h at 0 ^{o}C the reaction mixture was cooled to -20 ^{o}C and acetic acid (3.61 mL) was added, removed from the cold bath, diluted with EtOAc (400 mL), washed with water (500 mL) and brine (500 mL), dried over Na_2SO_4, filtered and evaporated under reduced pressure. The residue was purified by silica gel column chromatography and eluted with 20-50% EtOAc in hexane to yield 2'-*O*-(2'-methoxyethyl) derivative of compound **23** (14.95 g, 80%). It was dissolved in THF (100 mL) and 1 M *tertra*-butylammonium fluoride in THF (41.00 mL, 27.32 mmL) was added and stirred at room temperature for 1 h. The reaction mixture was diluted with EtOAc (400 mL) and the organic phase was washed with water (400 mL), brine (400 mL), dried (Na_2SO_4), filtered and concentrated under reduced pressure. The

residue thus obtained was purified by silica gel column chromatography and eluted with 50-70% EtO Ac in hexane to yield **23** (12.32 g, 97%). ^{1}H NMR (300MHz, DMSO-d$_6$): δ 8.02 - 7.77 (m, 3H), 7.59 - 7.47 (m, 3H), 7.43 (s, 1H), 7.39 - 7.12 (m, 11H), 5.99 (d, *J*=5.7 Hz, 1H), 5.47 - 5.25 (m, 2H), 5.02 - 4.73 (m, 4H), 4.68 - 4.47 (m, 3H), 4.42 - 4.21 (m, 3H), 3.84 - 3.60 (m, 5H), 3.53 - 3.37 (m, 2H), 3.25 - 3.05 (m, 3H), 1.59 - 1.26 (m, 3H); ^{13}C NMR (75MHz, CDCl$_3$): δ 163.3, 150.7, 138.0, 137.7, 134.6, 134.1, 133.2, 128.6, 128.4, 128.3, 128.0, 127.9, 127.7, 127.6, 127.6, 127.4, 127.3, 126.2, 126.1, 110.1, 89.6, 82.8, 80.9, 74.0, 72.3, 72.22, 72.2, 71.7, 70.5, 69.7, 60.6, 59.0, 12.5; HR MS (ESI) calcd for $C_{40}H_{45}N_2O_9$ $[M + H]^+$ *m*/*z* = 697.3081, found 697.3084.

Compound 24. To a solution of compound **23** (3.1 g, 4.45 mmol) in DMF (20 ml) was added 60% NaH (534 mg, 13.36 mmol)wa added at 0 ^{o}C and the reaction mixture was stirred at 0 ^{o}C for 1 h under argon atmosphere. To this methyl iodide (0.83 ml, 13.36 mmol) was added and stirring continued at 0 ^{o}C for additional 30 min. The reaction mixture was diluted with ice-water (100 mL) and extracted with EtOAc. The organic layer dried (Na_2SO_4), filtrated and concentrated under reduced pressure. The residue obtained was purified by silica gel column chromatograph and eluted with 50% EtOAc in hexane to afford compound **24** (2.96 g, 93%). ^{1}H NMR (300MHz, DMSO-d$_6$): δ 7.99 - 7.81 (m, 4H), 7.61 - 7.46 (m, 3H), 7.43 (s, 1H), 7.37 - 7.16 (m, 10H), 5.96 (d, *J*=5.3 Hz, 1H), 5.33 (s, 2H), 4.90 - 4.70 (m, 3H), 4.66 - 4.48 (m, 3H), 4.39 - 4.25 (m, 2H), 4.21 (t, *J*=3.8 Hz, 1H), 3.89 (d, *J*=4.3 Hz, 1H), 3.73 - 3.62 (m, 2H), 3.55 (t, *J*=4.8 Hz, 2H), 3.49 - 3.38 (m, 2H), 3.24 (s, 3H), 3.18 (s, 3H), 1.48 (s, 3H); ^{13}C NMR (75MHz, DMSO-d$_6$): δ 162.5, 150.8, 138.4, 138.0, 135.7, 135.0, 132.7, 132.5, 128.2, 128.2, 127.8, 127.7, 127.6, 127.4, 127.3, 127.3, 126.8, 126.2, 126.1, 126.0, 125.9, 109.1, 86.9, 82.1, 79.6, 77.1, 75.0, 71.4, 71.1, 71.0, 70.3, 69.1, 58.5, 58.1, 54.9, 12.2; LR MS (ESI) calcd for $C_{41}H_{47}N_2O_9$ $[M + H]^+$ *m*/*z* = 710.8, found 711.3.

Compound 25. To a solution of compound **23** (2.5 g, 3.59 mmol) in CH_2Cl_2 (20 ml) diethylaminosulfur trifluoride (DAST, 1.32 mL, 7.16 mmol) was added and stirred at room temperature for 1 h. The reaction mixture was diluted with aqueous saturated $NaHCO_3$ solution (100 mL) and extracted with EtOAc (2 x 100 mL). The organic phase dried (Na_2SO_4), filtered and concentrated. The residue was purified by silica gel column chromatography and eluted with 50% EtOAc in hexane to yield **25** (1.2 g, 47.8%). ^{1}H NMR (300MHz, DMSO-d_6): δ 5.95 (d, *J*=5.5 Hz, 1H), 5.34 (s, 2H), 4.85 - 4.72 (m, 4H), 4.72 - 4.54 (m, 3H), 4.41 - 4.26 (m, 1H), 4.26 - 4.12 (m, 1H), 3.76 - 3.62 (m, 2H), 3.45 (d, *J*=4.7 Hz, 2H), 3.18 (s, 3H), 1.58 (s, 3H); LR MS (ESI) calcd for $C_{40}H_{43}FN_2O_8$ $[M + H]^+$ *m/z* = 699.8, found 699.4.

Compound 26. To a solution of compound **S8** (2.1 g, 3.02 mmol) in anhydrous pyridine(15 ml) trifluoroacetic anhydride (0.76 g, 3.62 mmol) was added and stirred at room temperature for 1 h. The reaction mixture was diluted with water (100 mL) and extracted with EtOAc (2 x 100 mL) and the EtOAc layer was dried (Na_2SO_4), filtered and concentrate under reduced pressure. The residue obtained was purified by silica gel column chromatography and eluted with 30-60% EtOAc in hexane to yield **26** (2.12 g, 88.7%). ^{1}H NMR (300MHz, $CDCl_3$): δ 7.84 - 7.75 (m, 4H), 7.49 (d, *J*=9.3 Hz, 3H), 7.36 - 7.24 (m, 11H), 5.82 (d, *J*=1.3 Hz, 1H), 5.56 - 5.42 (m, 2H), 4.89 (d, *J*=10.9 Hz, 1H), 4.78 - 4.53 (m, 6H), 4.35 - 4.16 (m, 3H), 4.01 (td, *J*=3.1, 5.7 Hz, 1H), 3.96 - 3.73 (m, 3H), 3.57 (t, *J*=4.4 Hz, 2H), 3.41 (td, *J*=4.1, 14.4 Hz, 1H), 3.32 (s, 3H), 1.54 (s, 3H); ^{13}C NMR (75MHz, $CDCl_3$): δ 163.2, 157.6, 150.7, 138.0, 137.1, 135.2, 134.2, 133.2, 128.7, 128.4, 128.3, 128.17, 127.9, 127.7, 127.6, 127.0, 126.4, 126.3, 125.8, 117.7, 110.4, 91.3, 82.6, 80.3, 74.6, 74.4, 72.3, 72.3, 72.2, 70.5, 69.9, 58.9, 38.8, 12.6; LR MS (ESI) calcd for $C_{42}H_{45}F_3N_3O_9$ $[M + H]^+$ *m/z* = 792.8, found 792.2.

Compound 27a. To the solution of compound **24** (2.4 g, 3.38 mmol) in CH_2Cl_2 (36 ml) was added H_2O (4.5 ml), followed by DDQ (959mg, 4.23 mmol). After stirring for 1.5 at room temperature the reaction was quenched by adding 10% $NaHSO_3$. The reaction mixture was extracted with CH_2Cl_2 (3 x 100 mL). The organic phase dried (Na_2SO_4), filtered and concentrated under reduced pressure. The residue obtained was purified by silica gel column chromatography and eluted with 70% EtOAc in hexane to yield compound **27a** (1.64g, 85%). ^{1}H NMR (300MHz, DMSO-d_6): δ 7.41 (d, *J*=1.1 Hz, 1H), 7.39 - 7.24 (m, 10H), 5.90 (d, *J*=6.0 Hz, 1H), 5.33 (s, 2H), 5.13 (d, *J*=5.7 Hz, 1H), 4.81 (d, *J*=12.1 Hz, 1H), 4.68 - 4.46 (m, 3H), 4.31 (d, *J*=4.0 Hz, 1H), 4.05 (t, *J*=5.7 Hz, 1H), 3.99 - 3.93 (m, 1H), 3.85 (d, *J*=4.3 Hz, 1H), 3.75 - 3.51 (m, 4H), 3.49 - 3.38 (m, 2H), 3.28 (s, 3H), 3.18 (s, 3H), 1.50 (d, *J*=0.9 Hz, 3H); ^{13}C NMR (75MHz, $CDCl_3$): δ 163.4, 150.9, 138.1, 138.0, 134.2, 128.5, 128.2, 127.8, 127.6, 127.6, 127.0, 110.1, 87.9, 84.0, 82.9, 77.2, 72.4, 72.2, 71.8, 71.7, 70.5, 70.1, 67.9, 59.2, 58.9, 12.5; LR MS (ESI) calcd for $[M + H]^+$ m/z = $C_{30}H_{39}N_2O_9$ 571.3, found 572.0.

Compound 27b To a solution of compound **27a** (1.5 g, 2.63 mmol) in DMF (20 ml) was added imidazole (1.79 g, 26.3 mmol), followed by TBDMSCl (2.98 g, 13.16 mmol). After stirring for for 18 h at room temperature the reaction mixture was diluted with water (200 mL) and extracted with EtOAc (2 x 100 mL). The organic phase dried (Na_2SO_4), filtered and concentrated under reduced pressure. The residue obtained was purified by silica gel column chromatography and eluted with 70% EtOAc in hexane to yield compound **27b** (1.7g, 94.4%). ^{1}H NMR (300MHz, DMSO-d_6): δ 7.44 (s, 1H), 7.40 - 7.22 (m, 11H), 5.90 (d, *J*=6.6 Hz, 1H), 5.33 (d, *J*=2.4 Hz, 2H), 4.92 - 4.72 (m, 1H), 4.68 - 4.40 (m, 4H), 4.23 - 4.06 (m, 1H), 4.03 - 3.91 (m, 1H), 3.85 (d, *J*=4.5 Hz, 1H), 3.68 - 3.48 (m, 4H), 3.44 - 3.34 (m, 2H), 3.28 (s, 3H), 3.14 (s, 3H), 0.94 - 0.83 (m, 9H), 0.08 (s, 6H); ^{13}C NMR (75MHz, CDCl3): δ 163.4, 151.1, 138.2, 138.0, 134.8, 128.5, 128.4,

127.7, 127.6, 126.9, 126.7, 110.1, 87.8, 84.8, 81.9, 77.8, 77.2, 72.4, 72.3, 72.1, 70.5, 69.7, 69.5, 59.1, 58.9, 25.7, 18.1, 12.5, -4.6, -5.0; LR MS (ESI) calcd for $[M + H]^+$ *m*/*z* = $C_{36}H_{54}N_2O_9Si$ 685.9, found 684.4.

Compound 27c. Compound **27b** (1.7 g, 2.48 mmol) was dissolved in MeOH (50 ml) and palladium hydroxide (0.5 g, 20 wt% Pd on carbon dry base) was added. The reaction mixture was flushed with H_2 gas and stirred at room temperature under H_2 atmosphere for 2 h. The reaction mixture was filtrated through a pad of celite and washed the celite pad thoroughly with EtOAc. Combined filtrate and the washings and concentrated under reduced pressure. The residue was then dissolved in MeOH (20 ml). Et_3N (1 ml) and stirred for 3 h at room temperature. The reaction mixture was concentrated under reduced pressure and purified by silica gel column chromatography and eluted with EtOAc to yield **27c** (0.92 g, 78%). ^1H NMR (300MHz, DMSO-d_6): δ 11.35 (s, 1H), 7.75 (s, 1H), 5.86 (d, *J*=6.8 Hz, 1H), 5.54 (d, *J*=5.1 Hz, 1H), 4.40 (d, *J*=2.8 Hz, 1H), 4.06 (dd, *J*=4.7, 6.8 Hz, 1H), 3.94 - 3.74 (m, 2H), 3.61 - 3.51 (m, 2H), 3.42 - 3.30 (m, 5H), 3.26 (s, 3H), 3.21 - 3.14 (m, 3H), 1.79 (s, 3H), 0.88 (s, 9H), 0.09 (s, 6H); LR MS (ESI) calcd for $C_{21}H_{39}N_2O_8Si$ $[M + H]^+$ *m*/*z* = 475.6, found 475.2.

Compound 27d. Compound **27c** (0.85 g, 1.79 mmol) was dissolved in anhydrous pyridine (6 ml) and was added 2, 6-lutidine (0.58 g, 5.38 mmol), followed by DMTCl (1.82 g, 5.38 mmol). After heating at 40 ^oC for 36 h the reaction mixture was concentrated under reduced pressure to get oil. The residue was partitioned between EtOAc (100 mL) and H_2O (100 mL). The organic phase was separated, dried (Na_2SO_4) and concentrated under reduced pressure The residue obtained was purified by silica gel column chromatography and eluted with 70% EtOAc in hexane to yield **27d** (1.2 g, 86.3%). ^1H NMR (300MHz, $CDCl_3$): δ 7.91 (s, 1H), 7.64 - 7.13 (m, 9H), 6.94 (s, 1H), 6.84 (m, 4H), 5.86 (d, *J*=7.2 Hz, 1H), 4.67 - 4.42 (m, 1H), 4.25 - 4.09 (m, 1H),

3.94 - 3.73 (m, 7H), 3.65 - 3.54 (m, 2H), 3.53 - 3.40 (m, 3H), 3.40 - 3.26 (m, 4H), 3.12 (s, 3H), 3.01 (d, *J*=10.4 Hz, 1H), 1.27 (s, 3H), 0.91 (s, 9H), 0.11 (d, *J*=2.1 Hz, 6H); LR MS (ESI) calcd for $C_{42}H_{55}N_2O_{10}Si$ $[M - H]^-$ *m/z* = 776.0, found 775.7.

Compound 28a. Compound **28a** (1.32g, 86.3%) was synthesized from **25** (1.75 g, 2.51 mmol), CH_2Cl_2 (24 ml) H_2O (3.0 ml) and DDQ (854 mg, 3.76 mmol) using the procedure used for the synthesis of compound **27a**. ^{1}H NMR (300MHz, $CDCl_3$): δ 7.46 - 7.33 (m, 11H), 5.93 (d, *J*=2.8 Hz, 1H), 5.56 - 5.41 (m, 3H), 5.02 - 4.86 (m, 1H), 4.81 - 4.53 (m, 7H), 4.37 (d, *J*=5.8 Hz, 1H), 4.23 - 3.99 (m, 3H), 3.90 (dd, *J*=3.0, 5.3 Hz, 1H), 3.80 - 3.67 (m, 1H), 3.64 - 3.45 (m, 2H), 3.38 (s, 2H), 1.48 (s, 3H); HR MS (ESI) calcd for $C_{29}H_{36}FN_2O_8$ $[M + H]^+$ *m/z* = 559.2401, found 559.2400.

Compound 28b. Compound **28b** (1.5g, 94.3%) was synthesized from compound **28a** (1.32 g, 2.36 mmol), DMF (15 ml), imidazole (1.61 g, 23.6 mmol), TBDMSCl (1.78 g, 11.82 mmol) using the same procedure used for the synthesis of compound **27b**. ^{1}H NMR (300MHz, DMSO-d_6): δ 7.51 (s, 1H), 7.44 - 7.30 (m, 10H), 5.87 (d, *J*=6.8 Hz, 1H), 5.34 (s, 2H), 4.81 (d, *J*=11.7 Hz, 1H), 4.74 - 4.37 (m, 5H), 4.31 - 4.13 (m, 1H), 3.93 (br. s., 2H), 3.65 - 3.49 (m, 2H), 3.44 - 3.34 (m, 2H), 3.14 (d, *J*=1.3 Hz, 3H), 1.65 (s, 3H), 0.95 - 0.72 (m, 9H), 0.17 - -0.08 (m, 6H); ^{13}C NMR (75MHz, $CDCl_3$): δ 163.4, 151.0, 138.0, 137.7, 135.0, 128.6, 128.3, 128.0, 127.7, 127.6, 127.3, 110.2, 89.1, 84.2, 83.4, 83.3, 81.9, 81.6, 72.9, 72.2, 72.0, 70.6, 69.7, 58.9, 25.7, 18.0, 12.6, -4.57, -5.01; LR MS (ESI) calcd for $C_{35}H_{49}FN_2O_8Si$ $[M + H]^+$ *m/z* = 673.8, found 673.3.

Compound **28c**. Compound **28c** (0.86 g, 83.2%) from compound **28b** (1.5 g, 2.23 mmol), MeOH (20 ml), H_2 gas and $Pd(OH)_2$ catalyst (0.5 g, 20 wt% Pd on carbon dry base) using the procedure used for the synthesis of compound **27c**. ^{1}H NMR (300MHz, DMSO-d_6): δ 11.37 (s, 1H), 7.70

(s, 1H), 5.88 - 5.72 (m, 2H), 4.43 (br. s., 3H), 4.14 (d, *J*=5.1 Hz, 1H), 3.96 - 3.73 (m, 1H), 3.63 - 3.50 (m, 2H), 3.46 - 3.36 (m, 2H), 3.25 - 3.06 (m, 4H), 1.80 (s, 3H), 0.88 (s, 11H), 0.14 - 0.02 (m, 7H); ^{13}C NMR (75MHz, $CDCl_3$): δ 163.6, 150.4, 139.2, 110.9, 92.5, 86.1, 84.8, 82.59, 80.2, 72.0, 70.6, 70.3, 69.9, 69.8, 58.9, 25.7, 18.1, 12.3, -4.68, -5.04; LR MS (ESI) calcd for $C_{20}H_{36}FN_2O_7Si$ $[M + H]^+$ *m/z* = 463.5, found 463.2.

Compound **28d.** Compound **28d** (0.85 g, 62%) was synthesized from compound **28c** (0.83 g, 1.80 mmol), anhydrous pyridine (6 ml), 2, 6-lutidine (0.58 g, 5.39 mmol), and DMTCl (1.82 g, 5.38 mmol) using the procedure for the synthesis of compound **27d**. ^{1}H NMR (300MHz, $CDCl_3$): δ 7.94 (s, 1H), 7.58 - 7.15 (m, 9H), 6.84 (d, *J*=8.8 Hz, 4H), 5.66 (d, *J*=6.9 Hz, 1H), 4.51 - 3.99 (m, 4H), 3.84 (dd, *J*=4.9, 7.0 Hz, 1H), 3.79 (s, 6H), 3.70 - 3.53 (m, 1H), 3.53 - 3.45 (m, 2H), 3.44 (s, 2H), 3.28 (s, 3H), 1.67 (s, 3H), 0.90 (s, 9H), 0.08 (d, *J*=5.2 Hz, 6H); ^{13}C NMR (75MHz, $CDCl_3$): δ 163.5, 158.8, 149.9, 145.6, 137.9, 135.9, 135.8, 130.4, 128.1,127.9, 127.1, 113.3, 110.7, 89.5, 87.4, 84.9, 79.5, 71.9, 71.7, 71.4, 70.7, 69.8, 58.9, 55.2, 25.7, 18.1, 12.0, -4.6, -5.0; LR MS (ESI) calcd for $C_{41}H_{52}FN_2O_9Si$ $[M - H]^-$ *m/z* = 763.9, found 763.6.

Compound 29a. Compound **29a** (1.10g, 66.7%) was synthesized from **26** (2.0 g, 2.53 mmol), CH_2Cl_2 (24 ml), H_2O (3 ml), and DDQ (0.63 g, 2.68 mmol) using similar procedure used for the synthesis of compound **27a**. ^{1}H NMR (300MHz, $CDCl_3$): δ 7.48 - 7.30 (m, 11H), 5.84 (m, 1H), 5.47 (br. s., 2H), 4.88 (d, *J*=11.1 Hz, 1H), 4.68 (m, 2H), 4.64 - 4.48 (m, 1H), 4.36 (m, 1H), 4.28 - 4.07 (m, 3H), 4.07 - 3.95 (m, 1H), 3.95 - 3.72 (m, 1H), 3.60 (d, *J*=4.6 Hz, 1H), 3.40 (s, 3H), 1.42 (s, 3H); ^{13}C NMR (75MHz, $CDCl_3$): δ 163.2, 158.1, 150.7, 138.1, 137.1, 134.0, 128.7, 128.3, 128.2, 127.6, 127.6, 110.3, 89.4, 85.0, 83.0, 74.0, 72.3, 71.8, 71.5, 70.7, 70.6, 66.9, 58.9, 37.8, 12.5; LR MS (ESI) calcd for $C_{31}H_{37}F_3N_3O_9$ $[M + H]^+$ *m/z* = 652.6, found 652.2.

Compound 29b. Compound **29b** (1.23 g, 93%) was synthesized from compound **29a** (2.0 g, 2.53 mmol), DMF (15 ml), imidazole (1.15 g, 16.9 mmol) and TBDMSCl (1.27 g, 8.44 mmol) using similar procedure used for the synthesis of compound **27b**. ^{1}H NMR (300MHz, $CDCl_3$): δ 7.45 - 7.23 (m, 11H), 7.12 (d, *J*=8.8 Hz, 1H), 5.57 (d, *J*=5.6 Hz, 1H), 5.53 - 5.41 (m, 2H), 4.77 - 4.59 (m, 4H), 4.36 (d, *J*=5.4 Hz, 1H), 4.18 (m, 1H), 4.05 (d, *J*=4.4 Hz, 1H), 3.86 (d, *J*=4.5 Hz, 1H), 3.67 (m, 3H), 3.51 (m, 2H), 3.38 - 3.22 (m, 3H), 1.81 (s, 3H), 1.08 - 0.74 (m, 9H), 0.18 - 0.05 (m, 6H); ^{13}C NMR (75MHz, $CDCl_3$): δ 163.2, 157.5, 157.0, 150.9, 137.9, 137.2, 137.0, 128.7, 128.3, 128.2, 128.0, 127.7, 127.6, 117.7, 110.5, 93.2, 84.4, 80.2, 75.3, 72.5, 72.3, 72.0, 70.6, 70.5, 70.0, 58.9, 39.2, 25.7, 18.1, 12.8, -4.6, -4.9; LR MS (ESI) calcd for $C_{37}H_{51}F_3N_3O_9Si$ $[M + H]^+$ *m*/*z* = 766.9, found 765.2.

Compound 29c. Compound **29c** (530 mg, 59.5%) was synthesized from compound **29b** (1.23 g, 1.61 mmol), MeOH (20 ml), H_2 gas $Pd(OH)_2$ catalyst (0.2 g, 20 wt% Pd on carbon dry base) using the procedure used for the synthesis of compound **27c**. ^{1}H NMR (300MHz, $CDCl_3$): δ 7.62 (br. s., 1H), 7.16 (s, 1H), 5.33 (d, *J*=6.6 Hz, 1H), 4.69 - 4.55 (m, 1H), 4.52 - 4.40 (m, 1H), 4.34 (br. s., 1H), 4.19 - 3.89 (m, 2H), 4.20 - 3.89 (m, 2H), 3.78 - 3.58 (m, 3H), 3.57 - 3.44 (m, 2H), 3.30 (s, 3H), 1.92 (m, 3H), 0.92 (s, 9H), 0.13 (s, 6H); ^{13}C NMR (75MHz, $CDCl_3$): δ 163.7, 158.4, 157.9, 151.0, 140.6, 117.8, 114.0, 111.3, 95.0, 87.9, 79.1, 72.1, 70.4, 70.1, 58.9, 42.5, 25.7, 18.1, 12.0, -4.6, -4.9; ^{19}F NMR (282MHz, $CDCl_3$): δ -75.65; LR MS (ESI) calcd for $C_{22}H_{37}F_3N_3O_8Si$ $[M + H]^+$ *m*/*z* = 555.6, found 555.2.

Compound 29d. Compound **29d** (0.52 g, 65.7%) was synthesized from compound **29c** (0.53 g, 0.96 mmol), anhydrous pyridine (3 ml), 2, 6-lutidine (0.33 ml, 2.87 mmol) and DMTCl (0.81 g, 2.39 mmol) using the procedure used for the synthesis of compound **27d**. ^{1}H NMR (300MHz,

$CDCl_3$): δ 7.40 - 7.01 (m, 10H), 6.84 (s,1H), 6.80 – 6.67 (m, 5H), 5.40 (d, *J*=5.2 Hz, 1H), 3.95 (t, *J*=3.9 Hz, 2H), 3.74 - 3.58 (m, 11H), 3.49 - 3.27 (m, 2H), 3.26 - 3.03 (m, 4H), 1.54 (s, 3H), 0.73 (s, 9H), -0.12 (s, 3H), -0.13 (s, 3H) ; ^{13}C NMR (75MHz, $CDCl_3$): δ 163.4, 159.0, 156.8, 156.3, 156.3, 150.1, 145.3, 138.4, 135.7, 130.4, 130.4, 128.1, 128.0, 127.2, 113.5, 113.4, 111.3, 92.3, 87.6, 86.2, 79.6, 72.0, 71.0, 70.3, 70.2, 58.9, 55.2, 40.7, 25.7, 18.1, 12.0, -4.5, -5.0; ^{19}F NMR (282MHz, $CDCl_3$): δ -76.15; LR MS (ESI) calcd for $C_{43}H_{53}F_3N_3O_{10}Si$ $[M - H]^-$ *m/z* = 857.0, found 856.3.

Compound 30a: To the solution of compound **27d** (0.67 g, 0.86 mmol) in THF (5 ml) was added 1.0 M TBAF in THF (1.29 ml, 1.29 mmol). The reaction was stirred at room temperature for 16 h. The reaction was quenched by adding water (100 mL) and extracted with EtOAc (3 x 50 mL). Organic layer dried (Na_2SO_4), filtered and concentrated under reduced pressure. The residue purified by silica gel column chromatography and eluted with EtOAc to yield **30a** (0.40 g, 70.8%). ^{1}H NMR (300MHz, $CDCl_3$): δ 8.50 (s, 1H), 7.62 - 7.12 (m, 9H), 6.97 (s, 1H), 6.82 (dd, *J*=2.1, 8.9 Hz, 4H), 5.91 (d, *J*=5.2 Hz, 1H), 4.59 (q, *J*=4.6 Hz, 1H), 4.09 (dd, *J*=2.9, 4.4 Hz, 1H), 4.00 - 3.85 (m, 2H), 3.78 (d, *J*=2.0 Hz, 6H), 3.75 - 3.65 (m, 1H), 3.64 - 3.46 (m, 4H), 3.44 - 3.34 (m, 4H), 3.14 (s, 3H), 3.07 (dd, *J*=2.9, 10.2 Hz, 1H), 1.46 (s, 3H); ^{13}C NMR (75MHz, $CDCl_3$): δ 163.4, 158.8, 158.8, 150.2, 146.1, 136.6, 136.3, 135.8, 130.3, 128.1, 127.9, 126.9, 113.3, 113.2, 110.9, 87.3, 86.7, 85.1, 81.8, 72.0, 72.0, 59.0, 58.7, 55.2, 11.8; LR MS (ESI) calcd for $C_{36}H_{43}N_2O_{10}$ $[M - H]^-$ *m/z* = 663.7, found 663.2.

Compound 30b. Compound **30a** (0.38 g, 0.58 mmol) was dissolved in anhydrous DMF (2 ml) and 1H-tetrazole (0.036 g, 0.52 mmol), *N*-methylimidazole (0.017 g, 0.21 mmol) and 2-cynoethyl-*N*,*N*,*N*',*N*'-tetraisopropylphosphordiamidite (0.17 g, 0.86 mmol) were added. After

strings for 3 h at room temperature under argon atmosphere reaction mixture was diluted with EtOAc (50 mL). The reaction mixture was washed with aq. saturated. $NaHCO_3$ solution (50 mL), dried over Na_2SO_4 and concentrated under reduced pressure. The residue thus obtained was purified by silica gel column chromatography and eluted with 70% EtOAc in hexane to yield **30b** (0.37 mg, 74.7%). ^{31}P NMR (121MHz, $CDCl_3$): δ 149.79, 148.75; HR MS (ESI) calcd for $[M - H]^-$ *m/z* = $C_{45}H_{58}N_4O_{11}P$ 861.3828, found 861.3829.

Compound **31a.** Compound **31a** (0.55 g, 71.5%).was synthesized from compound **28d** (0.9 g, 1.18 mmol), THF (5 ml), 1.0 M TBAF in THF (2.36 ml, 2.36 mmol) using the procedure used for the synthesis of compound **30a**. ^{1}H NMR (300MHz, $CDCl_3$): δ 7.62 - 7.17 (m, 10H), 6.94 - 6.77 (m, 4H), 5.82 (d, *J*=5.7 Hz, 1H), 4.56 - 4.39 (m, 2H), 4.37 - 4.19 (m, 1H), 4.18 - 4.04 (m, 3H), 3.93 - 3.85 (m, 2H), 3.79 (d, *J*=1.1 Hz, 6H), 3.66 - 3.51 (m, 5H), 3.38 (s, 3H), 1.56 (s, 3H); ^{13}C NMR (75MHz, $CDCl_3$): δ 163.5, 158.8, 158.8, 150.1, 145.7, 136.2, 136.0, 135.9, 130.3, 130.2, 128.0, 127.1, 113.3, 111.1, 87.5, 87.4, 84.1, 81.1, 71.8, 71.6, 70.1, 68.8, 59.0, 55.2, 11.9; LR MS (ESI) calcd for $C_{35}H_{38}FN_2O_9$ $[M - H]^-$ *m/z* = 649.7, found 649.7.

Compound **31b.** Compound **31b** (0.52 g, 79.5%) was synthesized form compound **31a** (0.50 g, 0.77 mmol), DMF (2 ml), 1H-tetrazole (0.048 g, 0.69 mmol), *N*-methylimidazole (0.023 g, 0.28 mmol) and 2-cynoethyl-*N*,*N*,*N*',*N*'-tetraisopropylphosphordiamidite (0.35 g, 1.15 mmol) using the procedure used for the synthesis of compound **30b**. ^{31}P NMR (121MHz, $CDCl_3$): δ 150.22, 149.27; HR MS (ESI) calcd for $C_{44}H_{55}FN_4O_{10}P$ $[M - H]^-$ *m/z* = 849.4817, found 849.4819.

Compound 32a. Compound **32a** (0.26 g, 59.2%) was synthesized from compound **29d** (0.50 g, 0.59 mmol), THF (5 ml), 1.0 M TBAF in THF (0.89 ml, 0.89 mmol) using procedure used for the synthesis of compound **30a**^{1}H NMR (300MHz, CHLOROFORM-d): δ 7.52 - 7.27 (m, 10H),

6.95 (s, 1H), 6.83 (d, *J*=8.7 Hz, 5H), 5.56 (d, *J*=4.1 Hz, 1H), 3.99 - 3.75 (m, 13H), 3.68 - 3.43 (m, 3H), 3.43 - 3.30 (m, 4H), 1.68 (s, 3H); ^{13}C NMR (75MHz, $CDCl_3$): δ 163.2, 158.9, 156.4, 156.1, 159.8, 150.1, 145.2, 138.1, 135.6, 130.2, 130.1, 128.1, 127.9, 127.2, 113.5, 113.4, 111.3, 92.2, 87.4, 86.1, 79.5, 72.3, 71.7, 70.3, 70.2, 58.7, 55.1, 40.6, 11.9; ^{19}F NMR (282MHz, $CDCl_3$): δ -76.03; LR MS (ESI) calcd for $C_{37}H_{39}F_3N_3O_{10}$ $[M - H]^-$ *m/z* = 742.7, found 742.3.

Compound 32b. Compound **32b** (0.19 g, 60.6%) was synthesized from compound **32a** (0.24 g, 0.33 mmol), DMF (2 ml), 1H-tetrazole (0.021 g, 0.30 mmol), *N*-methylimidazole (0.01 g, 0.12 mmol) and 2-cynoethyl-*N*,*N*,*N*',*N*'-tetraisopropylphosphordiamidite (0.15 mg, 0.49 mmol) using the procedure used for the synthesis of compound **30b**. ^{31}P NMR (121MHz, $CDCl_3$): δ 149.76, 148.91; LR MS (ESI) calcd for $[M - H]^-$ *m/z* = $C_{46}H_{56}F_3N_5O_{11}P$ 943.0, found 942.7.

Compound S6. To a solution of compound **23** (2.0 g, 2.87 mmol) in anhydrous pyridine (10 ml) was added methanesulfonyl chloride (823 mg, 7.18 mmol) and stirred at room temperature for 4 h under argon atmosphere. The solvent was removed under reduced pressure an dthe residue dissolved in EtOAc (100 mL). The resulting solution was washed with water and dreies (Na_2SO_4), filtered and concentrated. The residue was purified by silica gel column chromatography and eluted with 30-50% EtOAc in hexane to yield compound **S6** (1.67 g, 75.2%). ^{1}H NMR (300MHz, DMSO-d_6): δ 8.05 - 7.77 (m, 5H), 7.61 - 7.43 (m, 4H), 7.40 - 7.17 (m, 12H), 5.94 (d, *J*=6.0 Hz, 1H), 5.34 (s, 2H), 4.88 - 4.72 (m, 3H), 4.69 - 4.49 (m, 4H), 4.44 - 4.26 (m, 3H), 4.24 - 4.14 (m, 1H), 4.12 - 3.94 (m, 1H), 3.75 - 3.58 (m, 2H), 3.51 - 3.37 (m, 2H), 3.18 (s, 3H), 3.17 (s, 3H), 1.62 (s, 3H); LR MS (ESI) calcd for $C_{41}H_{47}N_2O_{11}S$ $[M + H]^+$ *m/z* = 775.9, found 775.2.

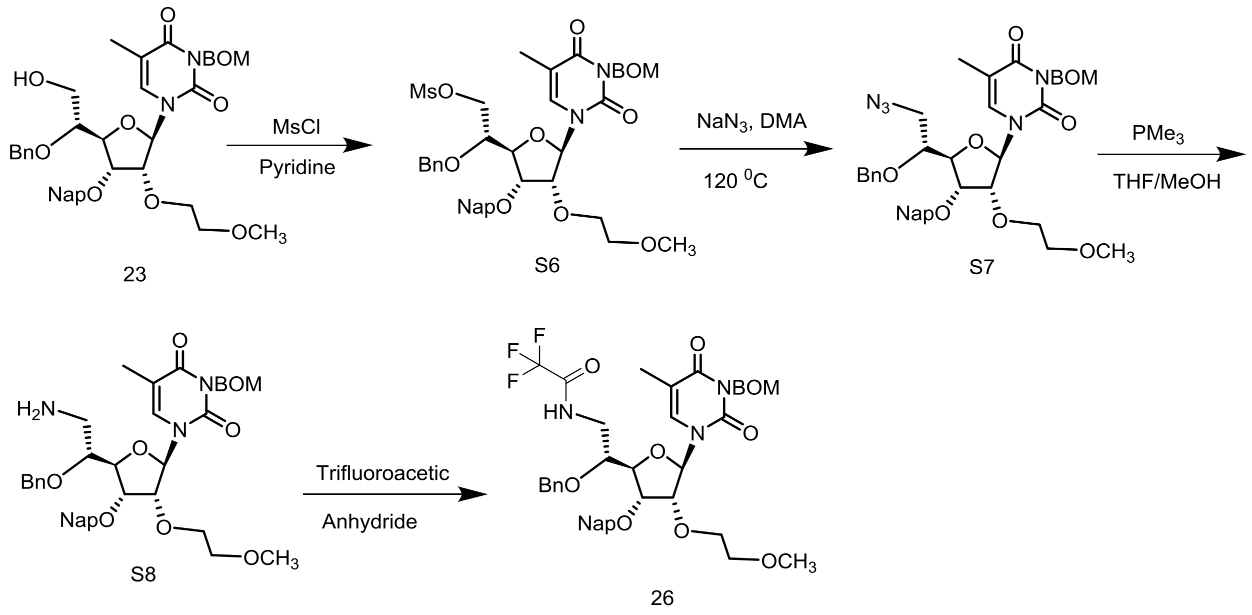

Scheme S2. Synthesis of compound **26**; Nap = 2-(methyl) naphthalene; BOM =benzyloxymethyl

Compound S7. Compound **S6** (2.31 g, 2.98 mmol) was dissolved in in *N*,*N*-dimethylacetamide (10 ml). To this NaN_3 (594 mg, 9.14 mmol) was added and the reaction mixture was heated at 120 ^{0}C for 3 h. The reaction mixture was cooled and diluted with water (200 mL) and the resulting solution was extracted with EtOAc (2 x 100 mL). The EtOAc layer was dried (Na_2SO_4) filtrated and concentrated. The residue obtained was purified by silica gel column chromatography and eluted with 50% EtOAc in Hexane to yield compound **S7** (2.26 g, 98%). ^{1}H NMR (300MHz, $CDCl_3$): δ 7.95 - 7.69 (m, 4H), 7.61 - 7.42 (m, 3H), 7.40 - 7.19 (m, 10H), 7.10 (s, 1H), 6.03 - 5.76 (m, 1H), 5.56 - 5.38 (m, 2H), 4.98 - 4.51 (m, 6H), 4.34 - 4.15 (m, 3H), 4.02 - 3.89 (m, 1H), 3.86 - 3.67 (m, 2H), 3.63 - 3.42 (m, 4H), 3.37 - 3.26 (m, 3H), 1.49 (s, 3H); ^{13}C NMR (75MHz, $CDCl_3$): δ 163.3, 150.7, 138.02, 137.4, 135.3, 134.8, 133.2, 133.1, 128.5, 128.3, 128.3, 127.9, 127.9, 127.7, 127.6, 127.3, 126.9, 126.3, 126.1, 125.9, 110.3, 90.6, 81.7,

80.3, 74.8, 73.1, 72.2, 72.1, 70.6, 70.0, 59.0, 51.0, 12.6. LR MS (ESI) calcd for $C_{40}H_{44}N_5O_8$ [M + H]$^+$ *m/z* =722.8, found 722.5

Compound S8. Trimethylphosphine (0.45 g, 5.91 mmol) was added to a solution of compound **S7** (2.13 g, 2.95 mmol) in THF (90 ml). Aqueous NaOH solution (2N, 30 ml) was added and the reaction mixture was stirred at room temperature for 4 h. Dilutted the reaction tmixture with water (100 mL) and aqueous layer was extracted with EtOAc (2 x 100 mL). The organic phase dried (Na_2SO_4), filtered and concentrated to yield compound **S8** (1.89 g, 92%). ^{1}H NMR (300MHz, $CDCl_3$): δ 7.83 (d, *J*=7.8 Hz, 4H), 7.51 (d, *J*=6.4 Hz, 3H), 7.43 - 7.11 (m, 11H), 6.00 (m, 1H), 5.49 (d, *J*=8.6 Hz, 2H), 4.81 - 4.52 (m, 6H), 4.38 (d, *J*=6.4 Hz, 1H), 4.32 - 4.23 (m, 1H), 4.16 (m, 1H), 3.81 (m, 2H), 3.59 (m, 2H), 3.41 - 3.28 (m, 4H), 2.95 (m, 2H), 1.50 (s, 3H); ^{13}C NMR (75MHz, $CDCl_3$): δ 163.3, 150.8, 138.1, 138.0, 134.9, 134.8, 128.5, 128.2, 127.9, 127.8, 127.7, 127.6, 127.6, 127.1, 126.9, 126.2, 126.1, 126.0, 110.2, 89.4, 82.3, 81.0, 79.7, 74.7, 72.5, 72.3, 72.2, 72.2, 59.0, 41.5, 12.6; LR MS (ESI) calcd for $C_{40}H_{46}N_3O_8$ [M + H]$^+$ *m/z* =696.3242, found 696.3243.

Compound 33a. Compound **23** (6.00 g, 8.62 mmol) was dissolved in CH_2Cl_2 (30 ml). To this (2,2,6,6-Tetramethylpiperidin-1-yl)oxy (TEMPO,0.30 g, 1.90 mmol) and iodobenzene diacetate (6.12 g, 19.0 mmol) were added. After stirring at room temperature for 2 h 50% acetonitrile in water (4 mL) was added and stirring continued for additional 18 h. Solvent was removed under reduced pressure and the residue was purified by silica gel column chromatography and eluted with EtOAc to yield compound **33a** (5.8 g, 94.8%). ^{1}H NMR (300MHz, DMSO-d_6): δ 8.03 - 7.77 (m, 4H), 7.62 - 7.45 (m, 4H), 7.42 - 7.13 (m, 10H), 5.99 (d, *J*=4.5 Hz, 1H), 5.47 - 5.21 (m, 2H), 4.91 - 4.68 (m, 4H), 4.63 - 4.40 (m, 5H), 4.37 - 4.17 (m, 2H), 3.86 - 3.57 (m, 2H), 3.45 (t,

J=4.1 Hz, 2H), 3.25 - 3.15 (m, 3H), 1.36 (s, 3H); HR MS (ESI) calcd for $[M + H]^+$ *m/z* = $C_{40}H_{43}N_2O_{10}$ 711.2878, found 711.2879.

Compound 33b. To a solution of ompound **33a** (1.96 g, 2.76 mmol) in CH_2Cl_2 (4 ml) 2.0 M oxalyl chloride in CH_2Cl_2 (11.0 ml, 22 mmol) and catalytic amount of DMF (2 drops) were added. The reaction mixture was stirred at room temperature for 1 h under argon atmosphere and the solvent was removed under reduced pressure. The residue was dissolved in MeOH (4 ml) and *N,N*-diisopropylethylamine (3.27 ml, 22 mmol) was added and stirred at room temperature for 1 h. The reaction mixture concentrated under reduced pressure and the residue was purified by silica gel column chromatography and eluted with 50% EtOAc in hexane to yield compound **33b** (1.36g, 68%). ^{1}H NMR (300MHz, DMSO-d_6) δ 8.17 - 7.70 (m, 4H), 7.63 - 7.45 (m, 4H), 7.45 - 7.18 (m, 10H), 5.98 (br. s., 1H), 5.33 (s, 2H), 4.90 - 4.67 (m, 3H), 4.66 - 4.48 (m, 4H), 4.40 (br. s., 1H), 4.28 (d, *J*=2.8 Hz, 2H), 3.87 - 3.57 (m, 5H), 3.56 - 3.40 (m, 2H), 3.29 - 3.10 (m, 3H), 1.44 (s, 3H); ^{13}C NMR (75MHz, $CDCl_3$): δ 169.3, 163.2, 150.8, 138.1, 136.7, 134.9, 134.1, 133.2, 133.1, 128.6, 128.2, 128.0, 127.9, 127.7, 127.6, 127.5, 126.8, 126.1, 126.0, 125.9, 110.0, 88.8, 81.7, 81.0, 74.3, 72.7, 72.4, 72.2, 72.1, 70.5, 69.8, 58.9, 52.1, 12.4; LR MS (ESI) calcd for $[M + H]^+$ *m/z* = $C_{41}H_{45}N_2O_{10}$ 725.8, found 725.3.

Compound 34a. Compound **34a** (675 mg, 83.6%) was synthesized from compound **33b** (1.0 g, 1.38 mmol), CH_2Cl_2 (8 ml), H_2O (1.0 ml), DDQ (470 mg, 2.07 mmol) using same procedure used for the synthesis of compound **27a**. ^{1}H NMR (300MHz, $CDCl_3$): δ 7.53 - 7.31 (m, 11H), 6.05 (d, *J*=6 Hz, 1H), 5.57 - 5.39 (m, 2H), 4.91 (d, *J*=11.5 Hz, 1H), 4.68 (s, 2H), 4.58 - 4.29 (m, 4H), 4.07 - 3.95 (m, 1H), 3.88 (t, *J*=4.1 Hz, 2H), 3.81 (s, 3H), 3.77 - 3.65 (m, 1H), 3.65 - 3.45 (m, 2H), 3.37 (s, 3H), 1.45 (s, 3H); ^{13}C NMR (75MHz, $CDCl_3$): δ 169.5, 163.3, 151.0, 138.1,

136.8, 134.1, 128.6, 128.3, 128.2, 127.7, 127.6, 127.6, 110.3, 87.7, 83.7, 82.4, 72.8, 72.2, 71.7, 70.6, 70.1, 67.8, 58.9, 52.3, 12.5; LR MS (ESI) calcd for $C_{30}H_{37}N_2O_{10}$ [M + H]+ *m/z* = 585.6, found 585.2.

Compound 34b. Compound **34b** (1.51 g, 97.4%) was synthesized from compound **34a** (1.3 g, 2.23 mmol), DMF (10 ml), imidazole (1.52 g, 22.3 mmol) and TBDMSCl (1.68 g, 11.13 mmol). Using the procedure used for the synthesis of compound **27b**. ^{1}H NMR (300MHz, $CDCl_3$): δ ^{1}H NMR (300MHz, $CDCl_3$): δ 7.43 - 7.31 (m, 11H), 6.13 (d, *J*=5.3 Hz, 1H), 5.54 - 5.44 (m, 2H), 4.90 (d, *J*=11.3 Hz, 1H), 4.67 (s, 2H), 4.54 - 4.31 (m, 4H), 4.01 - 3.90 (m, 1H), 3.78 (s, 3H), 3.74 (d, *J*=5.5 Hz, 1H), 3.65 (t, *J*=4.6 Hz, 1H), 3.54 - 3.42 (m, 2H), 3.25 (s, 3H), 1.48 (s, 3H), 0.89 (s, 9H), 0.10 (s, 3H), 0.07 (s, 3H); ^{13}C NMR (75MHz, $CDCl_3$): δ 169.4, 163.3, 151.3, 138.0, 136.8, 134.3, 128.6, 128.2, 128.2, 127.6, 127.6, 127.5, 110.3, 87.2, 84.7, 81.7, 78.0, 72.8, 72.1, 72.1, 70.6, 69.6, 69.6, 58.9, 52.2, 25.7, 18.0, 12.6, -4.7, -5.1; LR MS (ESI) calcd for $C_{36}H_{51}N_2O_{10}Si$ $[M + H]^+$ *m/z* =699.8, found 699.4.

Compound 35a. Compound **35a** (0.79 g, 74.8%) was synthesized from compound **34b** (1.5 g, 2.15 mmol), MeOH (10 ml), H_2 gas and palladium hydroxide (0.5 g, 20 wt% Pd on carbon dry base) using the same procedure used for the synthesis of compound **27c**. ^{1}H NMR (300MHz, $CDCl_3$): δ 8.33 (br. s., 1H), 7.64 (s, 1H), 5.86 (d, *J*=5.1 Hz, 1H), 4.63 - 4.52 (m, 1H), 4.49 - 4.38 (m, 1H), 4.30 - 4.19 (m, 1H), 4.11 (d, *J*=5.1 Hz, 1H), 3.83 (s, 3H), 3.82 - 3.73 (m, 2H), 3.72 - 3.63 (m, 1H), 3.58 - 3.43 (m, 2H), 3.31 (s, 3H), 1.63 (s, 3H), 0.95 (s., 9H), 0.08 (s, 3H), 0.04 (s, 3H); ^{13}C NMR (75MHz, $CDCl_3$): δ 171.9, 163.9, 150.5, 137.2, 110.8, 88.8, 86.0, 81.8, 77.4, 77.0, 76.6, 72.0, 70.6, 69.8, 69.3, 58.9, 52.9, 25.6, 18.0, 12.4, -4.7, -5.2; LR MS (ESI) calcd for $C_{21}H_{37}N_2O_9Si$ $[M + H]^+$ *m/z* =489.6, found 489.2.

Compound 35b. The compound **35b** (0.77 g, 73.3%) was synthesized from compound **35a** (0.55 g, 1.13 mmol), pyridine (4 ml), 2, 6-lutidine (0.78 ml, 6.76 mmol) and DMTCl (2.29 g, 6.76 mmol) using the same procedure used for the synthesis of compound **27d**. ^{1}H NMR (300MHz, $CDCl_3$): δ 7.50 - 7.38 (m, 2H), 7.36 - 7.24 (m, 7H), 7.17 (s, 1H), 6.81 (d, *J*=8.3 Hz, 4H), 6.03 (d, *J*=2.7 Hz, 1H), 4.69 (s, d, *J*=3.2 Hz, 1H), 4.48 (br. s., 1H), 4.17 (s, 1H), 3.90 (d, *J*=10.8 Hz, 2H), 3.78 (s, 6H), 3.77 - 3.53 (m, 4H), 3.34 (s, 3H), 3.25 (s, 3H), 1.77 (s, 3H), 1.11 (s, 9H), 0.10 (s, 3H), 0.07 (s, 3H); ^{13}C NMR (75MHz, $CDCl_3$): δ 170.5, 163.4, 158.9, 150.3, 143.9, 136.3, 135.0, 130.8, 128.8, 127.8, 127.4, 113.1, 110.9, 88.6, 87.1, 85.7, 80.6, 73.0, 72.1, 70.3, 70.0, 58.9, 55.2, 51.6, 25.7, 18.0, 11.8, -4.7, -5.0; LR MS (ESI) calcd for $C_{42}H_{53}N_2O_{11}Si$ $[M - H]^-$ *m/z* = 790.0, found 789.6.

Compound **35c.** Compound **35c** (0.54 g, 85.3%) was synthesized from compound **35b** (0.74 g, 0.94 mmol), THF (5 ml), 1.0 M TBAF in THF (1.41 ml, 1.41 mmol) using the procedure used for the synthesis of compound **30a**. ^{1}H NMR (300MHz, $CDCl_3$): δ 7.51 - 7.39 (m, 2H), 7.38 - 7.23 (m, 7H), 7.18 (s, 1H), 6.81 (d, *J*=7.9 Hz, 4H), 6.05 (d, *J*=4.2 Hz, 1H), 4.62 (d, *J*=3.3 Hz, 1H), 4.43 (d, *J*=5.5 Hz, 1H), 4.19 (dd, *J*=3.3, 5.5 Hz, 1H), 4.00 - 3.84 (m, 2H), 3.79 (s, 6H), 3.74 - 3.42 (m, 4H), 3.37 (s, 3H), 3.26 (s, 3H), 1.34 (s, 3H); ^{13}C NMR (75MHz, $CDCl_3$): δ 170.3, 163.4, 159.0, 150.2, 144.0, 135.6, 135.2, 135.1, 130.8, 128.9, 127.8, 127.4, 113.1, 111.3, 88.6, 87.4, 84.6, 81.6, 72.8, 71.7, 70.3, 68.6, 58.9, 55.3, 51.7, 11.5; LR MS (ESI) calcd for $C_{36}H_{39}N_2O_{11}$ $[M - H]^-$ *m/z* = 675.7, found 675.4.

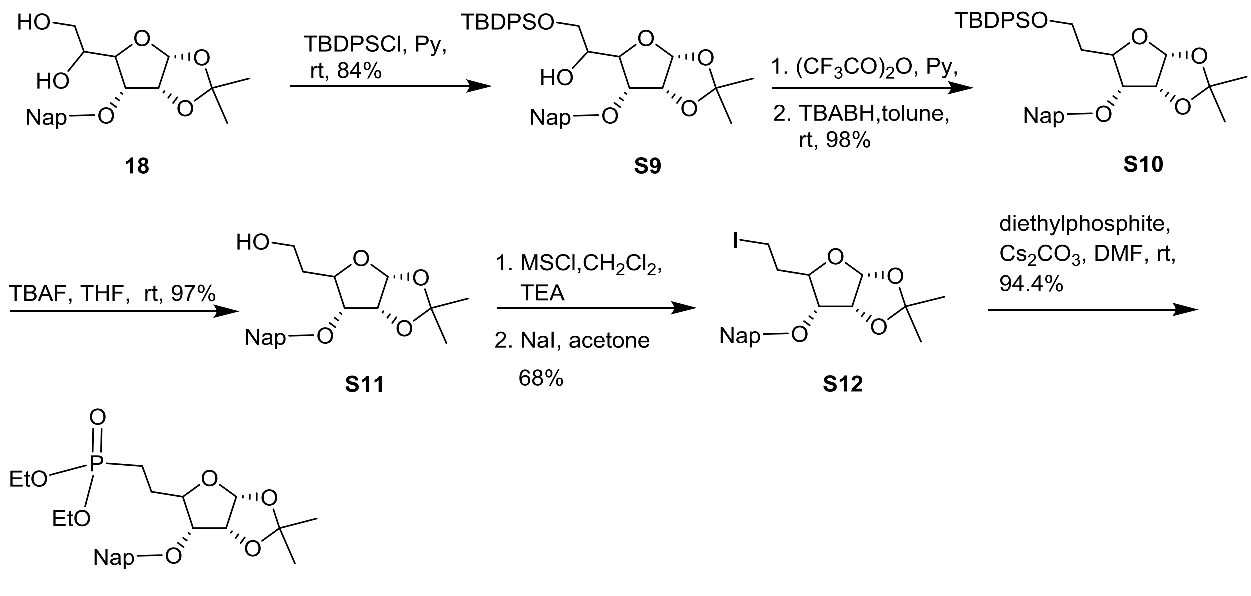

Scheme S3. Synthesis of compound **S13**; Nap = 2-(methyl)naphthalene; TBDPS = *tert*-butyldiphenylsilyl

Compound **36.** The compound **36** (0.55 g, 78.8%) was synthesized from compound **35c** (0.54 g, 0.80 mmol), DMF (2 ml), 1H-tetrazole (0.045 g, 0.64 mmol), *N*-methylimidazole (0.016 mL , 0.20 mmol) and 2-cynoethyl-*N*,*N*,*N*',*N*'-tetraisopropylphosphordiamidite (0.38 ml, 1.2 mmol) following the same procedure used for the synthesis of compound **30b**. ^{31}P NMR (121MHz, $CDCl_3$): δ 149.5, 148.8; HR MS (ESI) calcd for $C_{45}H_{56}N_4O_{12}P$ $[M - H]^-$ *m/z* = 875.4839, found 875.4837.

Compound S9. Compound **18** (49.7 g, 138.00 mmol) was co-evaporated with anhydrous pyridine (2 x 300 mL). The residue dissolved in anhydrous pyridine (500 mL) and *tert*-butyldiphenylchlorosilane (TBDPSCl, 37.00 g, 138 mmol) was added. The reaction mixture was stirred for 16 h at room temperature. Concentrated the reaction mixture to get an oil and the oil thus obtained was diluted with EtOAc (500 mL) and washed with saturated aqueous $NaHCO_3$ (3

x 300 mL), water (500 mL), brine (500 mL), dried (Na_2SO_4), filtered and concentrated under reduced pressure and the residue purified by silica gel column chromatography and eluted with 30% EtOAc in hexane to yield **S9** (65.32 g, 79.12%). ^{1}H NMR (300MHz, DMSO-d_6): δ 7.93 - 7.70 (m, 4H), 7.62 (ddd, *J*=1.4, 7.9, 11.4 Hz, 3H), 7.53 - 7.22 (m, 10H), 5.73 (d, *J*=3.8 Hz, 1H), 5.07 (d, *J*=4.7 Hz, 1H), 4.89 - 4.68 (m, 2H), 4.59 (d, *J*=12.1 Hz, 1H), 4.19 (dd, *J*=1.9, 8.7 Hz, 1H), 4.10 - 3.96 (m, 1H), 3.91 (d, *J*=6.4 Hz, 1H), 3.79 - 3.65 (m, 1H), 3.65 - 3.51 (m, 1H), 1.48 (s, 3H), 1.32 (s, 3H), 0.96 (s, 9H); LR MS (ESI) calcd for $C_{36}H_{42}O_6SiNa$ $[M + Na]^+$ m/z = 621.8, found 621.4.

Compound S10. To a solution of compound **S9** (35.50 g, 59.41 mmol) in anhydrous CH_2Cl_2 (600 mL) was added anhydrous pyridine (13.20 mL, 161.40 mmol) followed by 6-(N,N-dimethylamino)pyrinde (DMAP, 0.8 g, 6.5 mmol) and the resulting mixture was cooled in an ice bath. To this trifluoromethanesulfonic anhydride (13.7 mL, 77.81 mmol) was added slowly and reaction mixture was stirred at room temperature for 1 h. The mixture was poured into ice-cold water (500 mL). The organic layer separated and the aqueous layer extracted with CH_2Cl_2 (2 x 200 mL). The organic phase washed with 1N HCl (500 mL), 10% aqueous $NaHCO_3$ (500 mL), brine (500 mL), dried (Na_2SO_4), filtered and concentrated under reduced pressure. The residue was purified by silica gel column chromatography and eluted with 20% EtOAc in hexane to yield trifluoromethanesulfonyl derivative. The trifluoromethanesulfonyl derivative dissolved in anhydrous toluene (300 mL) and *tetra*-n-butylammoniumborohydride (TBABH, 41.7 g, 161.8 mmol) was added and heated at 86-90 ^oC for 2 h. Solvent was removed under reduced pressure and The residue was purified by silica gel column chromatography and eluted with 20-50% EtOAc in hexane to yield **S10** (33.86 g, 98 %). HR MS (ESI) calcd for $C_{36}H_{42}O_5SiNa$ $[M + Na]^+$ m/z = 605.2657, found 605.2659.

Compound S11. Compound **S11** (19.86 g, 97%) was synthesized form compound **S10** (34.6 g, 59.4 mmol), 1M TBAF in THF (260 mL) usin the procedure used for the synthesis of compound **30a**. ^{1}H NMR (300MHz, $CDCl_3$): δ 7.93 - 7.74 (m, 4H), 7.59 - 7.42 (m, 3H), 5.73 (d, *J*=4.0 Hz, 1H), 4.96 (d, *J*=11.9 Hz, 1H), 4.72 (d, *J*=11.9 Hz, 1H), 4.61 (t, *J*=4.1 Hz, 1H), 4.26 - 4.12 (m, 1H), 3.82 - 3.69 (m, 2H), 3.54 (dd, *J*=4.3, 9.0 Hz, 1H), 3.30 - 3.11 (m, 1H), 2.02 - 1.89 (m, 1H), 1.85 - 1.73 (m, 1H), 1.61 (s, 3H), 1.37 (s, 3H); ^{13}C NMR (75MHz, $CDCl_3$): δ 134.5, 133.0, 128.3, 127.7, 127.6, 126.9, 126.1, 126.0, 125.7, 112.7, 103.9, 81.5, 77.1, 76.7, 72.1, 60.1, 34.8, 26.4; LR MS (ESI) calcd for $C_{20}H_{24}O_5Na$ $[M + Na]^+$ *m/z* = 367.1503, found 367.1502.

Compound S12. Compound **S11** (20.14 g, 58.52 mmol) was dried over P_2O_5 under reduced pressure overnight. The dried **S11** was dissolved in anhydrous CH_2Cl_2 (300 mL) and cooled in ice bath under argon atmosphere. To this triethylamine (TEA, 24.52 mL, 175.92 mmol) was added followed by methanesulfonyl chloride (MSCl, 9.1 mL, 116.68 mmol) and stirred at 0 ^{o}C for 1 h under argon atmosphere. The reaction mixture was diluted with CH_2Cl_2 (150 mL) and acetic acid (40 mL) was added. The resulting organic phase was washed with water (300 mL), brine (300 mL), dried (Na_2SO_4), filtered and concentrated under reduced pressure. The residue was dissolved in acetone (60 mL) and sodium iodide (17.5 g, 117.04 mmol) was added. After refluxing for 1h the reaction mixture was cooled, diluted with EtOAc (300 mL) washed with water (300 mL), brine (300 mL), dried (Na_2SO_4), filtered and concentrated under reduced pressure to yield **S12** (21.94 g, 82 %). ^{1}H NMR (300MHz, $CDCl_3$): δ 7.94 - 7.76 (m, 4H), 7.56 - 7.43 (m, 3H), 5.68 (d, *J*=3.8 Hz, 1H), 4.94 (d, *J*=12.1 Hz, 1H), 4.69 (d, *J*=12.1 Hz, 1H), 4.57 (t, *J*=4.0 Hz, 1H), 4.21 - 4.02 (m, 1H), 3.45 (dd, *J*=4.2, 8.9 Hz, 1H), 3.30 - 3.16 (m, 2H), 2.27 (dddd, *J*=3.3, 7.4, 9.5, 14.1 Hz, 1H), 2.01 - 1.88 (m, 1H), 1.62 (s, 3H), 1.36 (s, 3H); ^{13}C NMR (75MHz, $CDCl_3$): δ 134.7, 133.1, 128.4, 127.7, 126.8, 126.2, 126.1, 125.7, 112.9, 103.8, 81.0,

77.8, 77.1, 72.1, 36.9, 26.6, 0.55; LR MS (ESI) calcd for $C_{20}H_{23}IO_4Na$ $[M + Na]^+$ *m/z* = 477.0496, found 477.0497.

Compound S13. Compound **S12** (21.92 g, 48.27 mmol) was dissolved in anhydrous DMF (240 mL) and diethylphosphite (11.24 mL, 87.12 mmol) and cesium carbonate (47.57 g 145.99 mmol) were added under argon atmosphere. The reaction mixture was stirred at room temperature for 18 h under argon atmosphere. The reaction mixture was diluted with EtOAc (300 mL) and washed with water (800 mL), brine (400 mL), dried (Na_2SO_4), filtered and concentrated under reduced pressure to yield **S13** (21.2 g, 94.4 %). ^{1}H NMR (300MHz, $CDCl_3$): δ 7.84 (td, *J*=4.4, 9.2 Hz, 4H), 7.58 - 7.39 (m, 3H), 5.69 (d, *J*=3.8 Hz, 1H), 4.93 (d, *J*=12.1 Hz, 1H), 4.72 (d, *J*=12.1 Hz, 1H), 4.58 (t, *J*=4.1 Hz, 1H), 4.20 - 3.94 (m, 5H), 3.45 (dd, *J*=4.3, 8.9 Hz, 1H), 2.15 - 1.95 (m, 1H), 1.94 - 1.69 (m, 3H), 1.61 (s, 3H), 1.34 (s, 3H), 1.31 - 1.19 (m, 6H); ^{13}C NMR (75MHz, $CDCl_3$): δ 134.7, 133.0, 132.9, 128.2, 127.7, 127.5, 126.7, 126.0, 125.9, 125.6, 112.6, 103.7, 81.1, 77.5, 77.2, 72.0, 61.4, 61.3, 26.5, 26.3, 25.1, 25.0, 22.5, 20.6; ^{31}P NMR (121MHz, $CDCl_3$): δ 31.74; HR MS (ESI) calcd for $C_{24}H_{33}O_7PNa$ $[M + Na]^+$ *m/z* = 487.1833, found 487.1833.

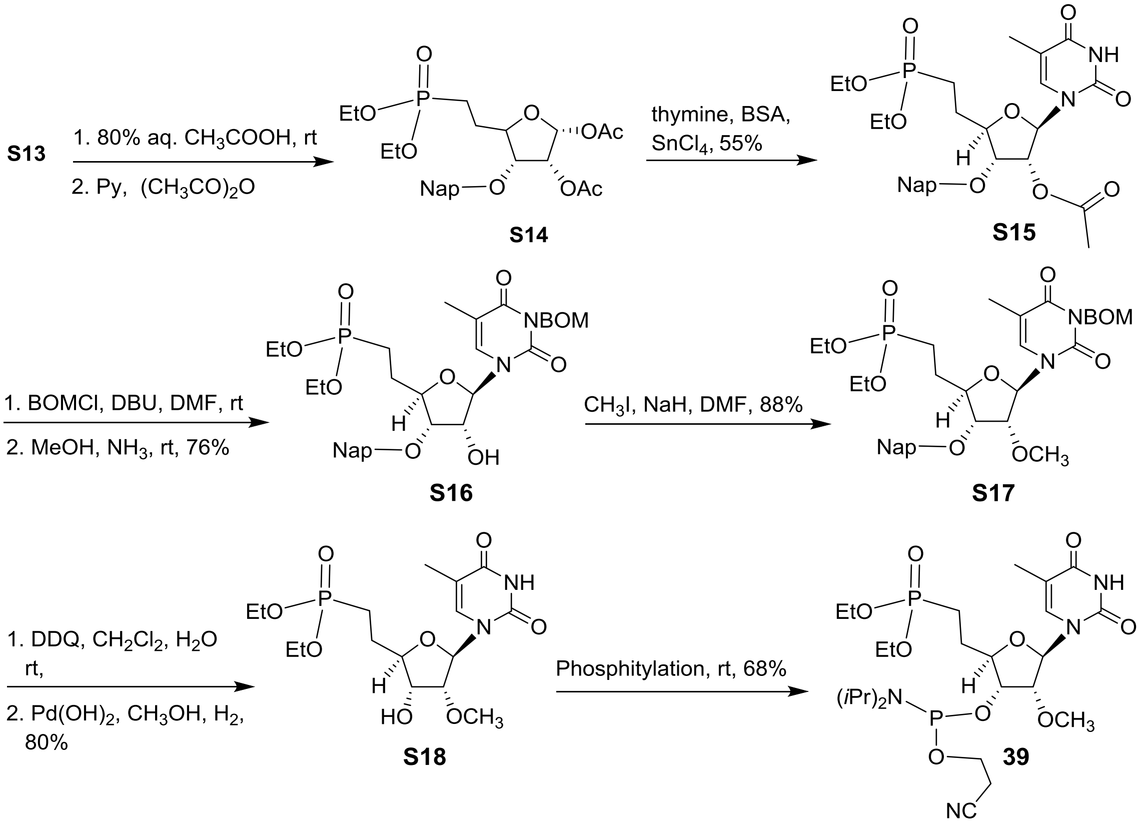

Scheme S4. Synthesis of compound **S19**; Nap = 2-(methyl)naphthalene; BOM = benzyloxy-methylene

Compound S14. Compound **S13** (4.00 g, 8.62 mmol) was dissolved in 80% aqueous acetic acid (89 mL) and heated at 90 ^{o}C for 18 h. Solvent was removed under reduced pressure. The residue was co-evaporated with anhydrous toluene (3 x 100 mL) and the residue obtained was dissolved in anhydrous pyridine (50 mL). The reaction mixture was cooled in an ice bath and acetic anhydride (8.31 mL, 88.61 mmol). The reaction mixture was removed from the ice bath and stirred at room temperature for 3 h under argon atmosphere. The reaction mixture was concentrated under reduced pressure and residue was diluted with EtOAc (150 mL) and washed washed with water (800 mL), brine (400 mL), dried (Na_2SO_4), filtered and concentrated under

reduced pressure The residue was purified by silica gel column chromatography and eluted with 5% methanol in CH_2Cl_2 to yield **S14** (3.65 g, 83 %). ^{1}H NMR (300MHz, $CDCl_3$): δ 7.90 - 7.80 (m, 4H), 7.54 - 7.42 (m, 3H), 5.35 (d, *J*=4.3 Hz, 1H), 4.86 - 4.72 (m, 1H), 4.70 - 4.54 (m, 1H), 4.21 - 3.88 (m, 7H), 2.14 (s, 3H), 2.06 (s, 3H), 1.97 - 1.64 (m, 4H), 1.37 - 1.13 (m, 6H); ^{31}P NMR (121MHz, $CDCl_3$): δ 31.61; HR MS (ESI) calcd for $C_{25}H_{33}O_9PNa$ $[M + Na]^+$ m/z = 531.1724, found 531.1726.

Compound S15. Compound **S14** (3.60 g, 7.07 mmol) and thymine (1.28 g, 10.16 mmol) were dried separately over P_2O_5 under reduced pressure over night. Thymine was suspended in anhydrous acetonitrile (9.2 mL) and *N*,*O*-bis(trimethylsilyl)acetamide (BSA, 9.2 mL) was added and heated at 60 ^{o}C for 2 h under argon atmosphere. A clear solution was obtained. The solvent was removed under reduced pressure and the residue co-evaporated with toluene (2 x 40 mL). To the residue a solution of compound **S14** in acetonitrile (55 mL) and tin tetrachloride (0.64 mL, 5.59 mmol) were added. After heating at 80 ^{o}C under argon atmosphere for 2 h the reaction mixture was poured into EtOAc (200 mL), washed with saturated aqueous $NaHCO_3$ (200 mL), water and brine (200 mL), dried (Na_2SO_4), filtered and concentrated under reduced pressure. The residue was purified by silica gel column chromatography and eluted with 5-10% methanol in CH_2Cl_2 to yield **S15** (2.23 g, 55%). LR MS (ESI) calcd for $C_{28}H_{36}N_2O_9P$ $[M + H]^+$ m/z =575.2, found 574.8

Compound S16. Compound **S16** (1.32 g, 70.5%) was synthesized from compound **S15** (2.14 g, 3.72 mmol), BOMCl (0.78 mL, 5.59 mmol), DBU (1.68 mL, 11.16 mmol), anhydrous DMF (20.1 mL) and methanolic ammonia (7M, 26 mL) using the procedure used for the synthesis of compound **22**.. ^{1}H NMR (300MHz, DMSO-d_6): δ 7.98 - 7.81 (m, 4H), 7.60 - 7.45 (m, 4H), 7.40 - 7.22 (m, 5H), 5.83 (d, *J*=5.3 Hz, 1H), 5.59 (d, *J*=6.0 Hz, 1H), 5.35 (s, 2H), 4.97 - 4.65 (m, 2H),

4.64 - 4.50 (m, 2H), 4.41 (d, *J*=5.8 Hz, 1H), 4.07 - 3.83 (m, 6H), 1.85 (s, 3H), 1.83 - 1.64 (m, 4H), 1.19 (t, *J*=7.1 Hz, 6H); ^{13}C NMR (75MHz, $CDCl_3$): δ 163.3, 150.9, 138.0, 135.6, 134.0, 133.2, 133.2, 128.7, 128.3, 127.9, 127.8, 127.7, 127.6, 127.1, 126.5, 126.4, 125.6, 110.4, 93.8, 81.4, 81.1, 73.3, 72.9, 72.3, 70.6, 61.7, 61.6, 26.4, 22.9, 16.5, 16.4, 13.1; ^{31}P NMR (121MHz, DMSO-d_6): δ 31.35; LR MS (ESI) calcd for $C_{34}H_{42}N_2O_9P$ $[M + H]^+$ *m/z* = 653.2589, found 653.2590.

Compound S17. Compound **S16** (0.86 g, 1.32 mmol) was dried over P_2O_5 under reduced pressure over night and then dissolved in anhydrous DMF (6 mL). The reaction mixture was cooled in an ice under argon atmosphere. To this sodium hydride (0.16 g, 3.96 mmol, 60% dispersion in mineral oil) and stirred for 15 min at 0 ^{o}C under argon atmosphere. Methyl iodide (0.25 mL, 3.96 mmol) was added and stiring continued for 18 h at 0-4 ^{o}C. The reaction was quenched by adding acetic acid (1 mL) and diluted with EtOAc (100 mL). The organic phase thus obtained was washed with water (100 mL) and brine (200 mL), dried (Na_2SO_4), filtered and concentrated under reduced pressure. The residue was purified by silica gel column chromatography and eluted with 5% methanol in CH_2Cl_2 to yield **S17** (0.80 g, 91%). ^{1}H NMR (300MHz, DMSO-d_6): δ 8.03 - 7.80 (m, 4H), 7.53 (d, *J*=4.7 Hz, 4H), 7.41 - 7.20 (m, 5H), 5.88 (d, *J*=4.5 Hz, 1H), 5.47 - 5.27 (m, 2H), 4.90 - 4.70 (m, 2H), 4.65 - 4.45 (m, 2H), 4.26 - 3.84 (m, 7H), 3.38 (s, 3H), 1.85 (s, 3H), 1.95 - 1.74 (m, 4H), , 1.20 (t, *J*=7.0 Hz, 6H); ^{13}C NMR (75MHz, $CDCl_3$): δ 163.3, 150.5, 137.9, 134.6, 134.2, 133.1, 133.1, 128.4, 128.3, 127.9, 127.8, 127.7, 127.6, 126.7, 126.3, 126.2, 125.7, 110.4 , 90.4, 81.7, 81.1, 80.8, 79.8, 77.2, 72.3, 70.5, 61.8, 61.7, 61.7, 61.7, 58.5, 26.5, 23.0, 21.1, 16.4, 13.3; ^{31}P NMR (121MHz, $CDCl_3$): δ 31.26; HR MS (ESI) calcd for $C_{35}H_{44}N_2O_9P$ $[M + H]^+$ *m/z* = 667.2743, found 667.2744.

Compound S18. To a sloution of compound **S17** (0.78 g, 1.17 mmol) in CH_2Cl_2 (14 mL)and 2,-4-dichloro-5,6-dicyano-1,4-benzoquinone (DDQ, 0.71 g, 3.12 mmol) and water (0.06 g, 3.12 mmol) were added. The reaction mixture was stirred at room temperature for 15 h. The reaction mixture was diluted with EtOAc (100 mL) and washed with water (100 mL) and brine (200 mL), dried (Na_2SO_4), filtered and concentrated under reduced pressure. The residue obtained was purified by silica gel column chromatography and eluted with 5% methanol in CH_2Cl_2. The 3'-Nap deprotected product thus obtained was dissolved in MeOH (12 ml) and palladium hydroxide (0.5 g, 20 wt% Pd on carbon dry base) was added. The reaction mixture was flushed with H_2 gas and stirred at room temperature under H_2 atmosphere for 16 h. The reaction mixture was filtrated through a pad of celite and washed the celite pad thoroughly with EtOAc. Combined filtrate and the washings and concentrated under reduced pressure. The residue was then dissolved in MeOH (20 ml). Et_3N (1 ml) and stirred for 3 h at room temperature. The reaction mixture was concentrated under reduced pressure and purified by silica gel column chromatography and eluted with 5-10% MeOH **S18** (0.29 g, 80%). ^{1}H NMR (300MHz, DMSO-d_6): δ 7.72 (d, *J*=1.3 Hz, 1H), 6.34 (d, *J*=4.0 Hz, 1H), 4.74 - 4.45 (m, 5H), 4.45 - 4.26 (m, 2H), 4.06 - 3.94 (m, 3H), 3.85 (br. s., 1H), 2.40 (s, 3H), 2.47 - 2.27 (m, 4H), 1.94 - 1.68 (m, 6H); ^{13}C NMR (75MHz, $CDCl_3$): δ 163.6, 150.0, 135.2, 111.4, 88.5, 83.3, 82.9, 82.7, 73.2, 61.7, 58.8, 26.2, 22.8, 20.8, 16.4, 12.6; ^{31}P NMR (121MHz, $CDCl_3$): δ 31.29; LR MS (ESI) calcd for $C_{16}H_{28}N_2O_8P$ $[M + H]^+$ *m*/*z* = 407.1568, found 407.1562.

Compound **39.** The compound **39** (0.23 g, 68%) was synthesized from compound **S18** (0.22 g, 0.54 mmol), DMF (2.3 ml), 1H-tetrazole (0.022 g, 0.48 mmol), *N*-methylimidazole (0.016 mL , 0.20 mmol) and 2-cynoethyl-*N*,*N*,*N*',*N*'-tetraisopropylphosphordiamidite (0.26 ml, 08 mmol) following the same procedure used for the synthesis of compound **30b**. ^{31}P NMR (121MHz,

$CDCl_3$): δ 150.30, 150.10, 31.50, 31.10; LR MS (ESI) calcd for $C_{25}H_{43}N_4O_9P_2$ [M - H]$^-$ *m/z* = 605.6, found 605.3.

Compound 41. To a solution of compound[1] **40** (100 g, 161.81 mmol) in pyridine (300 ml) benzoyl chloride (34.12 g, 242.72 mmol) was added and stirred at room temperature for 3 h. The solvent was removed under reduced pressure and the residue was dissolved in EtOAc (500 mL) and washed with water (3 x 500 mL), dried (Na_2SO_4), filtered. The EtOAc layer was concentrated under reduced pressure and the residue was dissolved in CH_2Cl_2 (1000 mL). To this trifluoroacetic acid (24.9 ml, 323.62 mmol) and triethylsilane (49 ml, 116.03 mmol) were added. After stirring at room temperature for 4 h, the reaction mixture was concentrated at reduced pressure. The residue thus obtained was dissolved in EtOAc (500 mL) and washed with saturated aqueous $NaHCO_3$ (400 mL), brine (400 mL), dries (Na_2SO_4) and concentrated under reduced pressure. The residue was purified by silica gel column chromatography to yield **41** (60 g, 88.2%). ^1H NMR (300MHz, DMSO-d_6)): δ 11.41 (s, 1H), 8.09 - 7.95 (m, 2H), 7.81 (s, 1H), 7.76 - 7.66 (m, 1H), 7.63 - 7.45 (m, 2H), 5.97 (d, *J*=6.8 Hz, 1H), 5.50 (td, *J*=2.9, 5.5 Hz, 1H), 5.44 - 5.28 (m, 1H), 4.48 - 4.33 (m, 1H), 4.25 (d, *J*=2.8 Hz, 1H), 3.71 (s, 4H), 3.64 - 3.47 (m, 2H), 3.42 - 3.16 (m, 4H), 1.81 (s, 3H); LR MS (ESI) calcd for $C_{20}H_{23}N_2O_8$ [M - Na]$^-$ *m/z* = 419.4, found 419.1.

Compound **42**. To a solution of compound **41** (30 g, 71.43 mmol) in DMSO (150 ml) *N,N'*-dicyclohexylcarbodiimide (DCC, 22.1 g, 107.14 mmol) and pyridiniumtrifluoroacetate (Py-TFA, 13.78 g, 71.43 mmol) were added. The reaction mixture was stirred at room temperature for 4 h to yield 5'-aldehyde of compound **41**. In a separate flask tetraethyl methylenediphosphonate (35.4 ml, 142.86 mmol) was dissolved in THF (150 ml), cooled to 0 ^oC and 1.0 M potassium *tert*-butoxide (KO*t*Bu, 142.86 ml, 142.86 mmol) was added. After stirring the reaction mixture at

0 ^{o}C for 10 min allowed the reaction to come to room temperature and stirring continued at room temperature for additional 30 min. The tetraethyl methylenediphosphonate anion solution thus obtained was cooled in an ice bath (0 ^{o}C) and to this the 5'-aldehyde solution of compound **41** was added *via* cannula. The resulting reaction mixture was stirred at 0 ^{o}C for 1h and subsequently allowed to come to room temperature. The reaction mixture was diluted with water (300 mL) and aqueous layer was extracted with EtOAc (3 x 300 mL). The EtOAc layer was washed with brine (500 mL), dried (Na_2SO_4) and concentrated under reduced pressure. The residue obtained was purified by silica gel column chromatography and eluted with EtOAc to yield **42** (21.2 g, 53.8%). ^{1}H NMR (300MHz, $CDCl_3$): δ 8.44 (s, 1H), 8.08 (dd, *J*=1.3, 8.3 Hz, 2H), 7.74 - 7.56 (m, 1H), 7.56 - 7.39 (m, 2H), 7.10 (d, *J*=1.1 Hz, 1H), 7.05 - 6.77 (m, 1H), 6.13 (ddd, *J*=1.7, 17.1, 18.7 Hz, 1H), 6.04 (d, *J*=4.5 Hz, 1H), 5.21 (t, *J*=5.8 Hz, 1H), 4.94 - 4.78 (m, 1H), 4.43 (t, *J*=5.0 Hz, 1H), 4.21 - 4.01 (m, 4H), 3.86 - 3.58 (m, 2H), 3.53 - 3.32 (m, 2H), 3.21 - 3.11 (m, 3H), 1.65 (s, 3H), 1.41 - 1.12 (m, 7H); ^{13}C NMR (75MHz, $CDCl_3$): δ 165.7, 163.3, 150.1, 146.3, 146.2, 135.6, 133.6, 129.8, 128.9, 128.5, 121.3, 118.8, 111.5, 89.7, 80.4, 79.8, 73.8, 72.1, 70.8, 62.2, 62.1, 62.0, 58.8, 16.4, 16.3, 12.6; ^{31}P NMR (121MHz, $CDCl_3$): δ 16.66; LR MS (ESI) calcd for $C_{25}H_{34}N_2O_{10}P$ $[M + H]^+$ *m/z* =553.5, found 553.2.

Compound **43a.** Compound **42** (27.4 g, 49.64 mmol) was dissolved in MeOH (300 ml) and palladium on carbon (2.72 g, 10 wt% Pd on carbon dry base) was added. The reaction mixture was flushed with H_2 gas and stirred at room temperature under H_2 atmosphere for 2 h. The reaction mixture was filtered through a pad of celite and washed the celite pad thoroughly with EtOAc. Combined filtrate and the washings concentrated under reduced pressure. The residue was dissolved in 7 *N* methanolic ammonium (200 mL) and stirred at room temperature for 18 h. The solvent was removed under reduced pressure and the residue was purified by silica gel

column chromatography and eluted first with EtOAc, then with 5% MeOH in CH_2Cl_2 to yield **43a** (7.5 g, 68%). ^{1}H NMR (300MHz, $CDCl_3$): δ 9.89 (s, 1H), 7.10 (d, *J*=1.0 Hz, 1H), 5.79 (d, *J*=2.9 Hz, 1H), 4.23 - 4.05 (m, 4H), 4.05 - 3.94 (m, 2H), 3.88 (br. s., 2H), 3.80 - 3.68 (m, 2H), 3.66 - 3.47 (m, 2H), 3.38 (s, 3H), 2.21 - 1.77 (m, 8H), 1.33 (t, *J*=7.0 Hz, 7H); ^{13}C NMR (75MHz, $CDCl_3$): δ 164.0, 150.2, 135.5, 111.0, 89.2, 83.1, 82.8, 82.1, 73.0, 71.6, 70.4, 61.7, 61.6, 61.6, 61.6, 26.3, 26.2, 22.7, 20.8, 16.4, 16.3, 12.5; ^{31}P NMR (121MHz, $CDCl_3$): δ 31.40; LR MS (ESI) calcd for $C_{18}H_{32}N_2O_9P$ $[M + H]^+$ *m/z* = 451.4, found 451.4.1.

Compound **43b**. Compound **43b** (8.45 g, 78%) was synthesized from compound **43a** (7.5 g, 16.67 mmol), DMF (10 ml), 1H-tetrazole (934 mg, 13.3 mmol), 1-methylimidazole (1.65 ml, 20.66 mmol) and 2-cynoethyl-*N*,*N*,*N*’,*N*’-tetraisopropylphosphordiamidite (0.33 ml, 4.17 mmol) using the procedure used for the synthesis of compound **30b**. ^{31}P NMR (121MHz, CD_3CN): δ 149.54, 149.50; LR MS (ESI) calcd for $C_{27}H_{47}N_4O_{10}P_2$ $[M - Na]^-$ *m/z* = 649.7, found 649.3.

Compound S19. Compound **18** (28.53 g, 79.21 mmol) was dissolved in dioxane (241 ml) and a solution of $NaIO_4$ (17.90 g, 83.69 mmol) in water (609 mL) was added over 40 min. After 90 min stirring at room temperature the reaction mixture was poured in EtOAc (300 mL) and the organic layer was separated, washed with water (300 mL), brine (300 mL), dried (Na_2SO_4) and concentrated to provide 5’-aldehyde. The residue was dissolved in 50% aqueous ethanol (624 mL) and $NaBH_4$ (6.5 g, 170.64 mmol) was added. The reaction mixture was stirred at room temperature 2 h and then neutralized with 10% aqueous acetic acid. The ethanol was removed under reduced pressure and the aqueous layer thus obtained was extracted with EtOAc (2 x 300 mL), wahed with brine (300 mL), dried (Na_2SO_4), filtered and concentrated under reduced pressure. The residue was purified by silica gel column and eluted with 20-30% EtOAc in

hexane to yield **S19** (15.66 g, 61%). ^{1}H NMR (300MHz, DMSO-d_6): δ 8.09 - 7.78 (m, 4H), 7.62 - 7.42 (m, 3H), 5.52 (d, *J*=3.6 Hz, 1H), 4.94 - 4.60 (m, 4H), 4.02 - 3.85 (m, 1H), 3.87 - 3.73 (m, 1H), 3.73 - 3.61 (m, 1H), 3.55 - 3.36 (m, 1H), 1.47 (s, 3H), 1.30 (s, 3H); ^{13}C NMR (75MHz, $CDCl_3$): δ 134.9, 133.1, 133.0, 128.2, 127.8, 127.6, 126.7, 126.10, 126.0, 125.7, 113.0, 104.0, 78.8, 77.5, 76.6, 72.3, 60.5, 26.7, 26.4; HR MS (ESI) calcd for $C_{19}H_{22}O_5Na$ $[M + Na]^+$ m/z = 353.1345, found 353.1346.

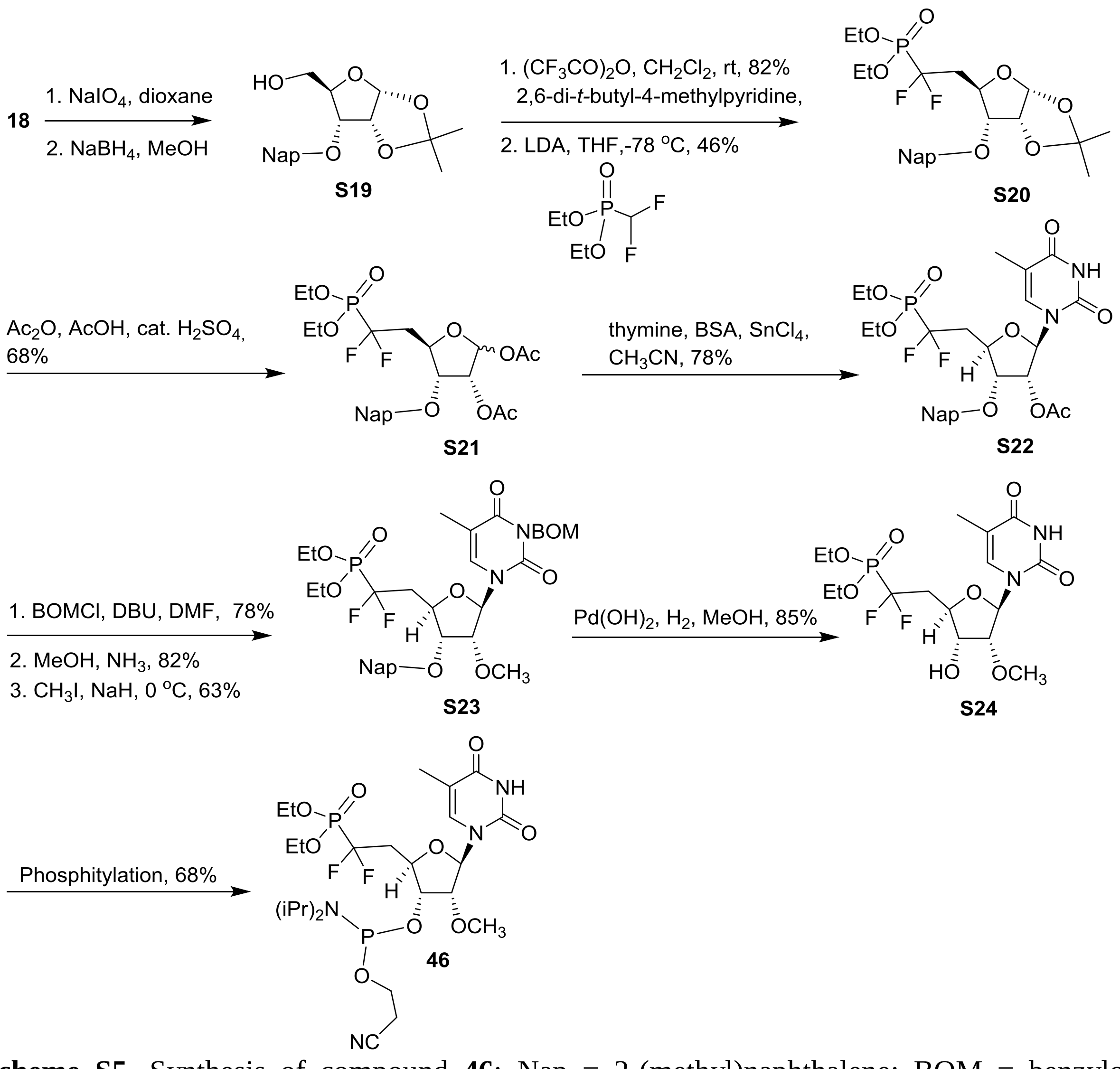

Scheme S5. Synthesis of compound **46**; Nap = 2-(methyl)naphthalene; BOM = benzyloxy-methylene

Compound S20. Compound **S19** (6.25 g, 18.93 mmol) was dried over P_2O_5 under reduced pressure overnight and it was dissolved in anhydrous CH_2Cl_2 (29 mL). To this 2,6-di-*tert*-butyl-4-methylpyridine (11.66 g, 56.79 mmol) was added and resulting mixture was cooled in a dry ice ethanol bath to -10 ^oC under argon atmosphere. To this a solution of trifluoromethanesulfonic anhydride (3.83 mL, 22.73 mmol) in anhydrous CH_2Cl_2 (4.8 mL) was added and stirred at -10 to 0 ^oC for 45 min. The reaction mixture was poured into ice cold water (100 mL) and extracted with EtOAc (200 mL). The organic layer was washed with ice cold water (200 mL) and brine (200 mL), dried (Na_2SO_4) and concentrated. The residue was purified by silica gel column chromate- graphy and eluted with 0 to 30% EtOAc in heane to get 5'-trifluoromethanesulfonyl derivative (9.17 gm 89%). To a solution of difluoromethylphosphonicacid diethyl ester (11.08 g, 58.88 mmol) was dissolved in anhydrous THF (45 mL) and resulting solution was cooled to -78 ^oC (acetone-dry ice). To this lithium diisopropylamine (LDA, 29.5 mL, 58.88 mmol, 2. M solution in heptane/hexane) was added drop wise and reaction mixture was stirred for 10 min at -70 ^oC. To this a solution of 5-trifluromethanesulphonyl derivative of **S19** from above in anhydrous THF (52 mL) was added slowly. Stirring continued for additional 30 min at -78 ^oC. The reaction mixture was diluted with aqueous saturated NH_4Cl (60 mL) and extracted with EtOAc (300 mL). The organic phase wasnhed with brine (200 mL), dried (Na_2SO_4), filtered and concentrated under reduced pressure to yield **S20** (8.23g, 97%). ^1H NMR (300MHz, $CDCl_3$): δ 8.00 - 7.74 (m, 4H), 7.59 - 7.40 (m, 3H), 5.74 (d, *J*=3.8 Hz, 1H), 4.95 (d, *J*=12.1 Hz, 1H), 4.72 (d, *J*=12.1 Hz, 1H), 4.55 (t, *J*=4.1 Hz, 1H), 4.42 (dt, *J*=1.9, 9.0 Hz, 1H), 4.38 - 4.23 (m, 4H), 3.47 (dd, *J*=4.1, 9.0 Hz, 1H), 2.44 (t, *J*=1.9 Hz, 1H), 2.33 - 2.04 (m, 1H), 1.62 (s, 3H), 1.60 (s, 3H), 1.46 - 1.25 (m, 6H); ^{13}C NMR (75MHz, $CDCl_3$): δ 134.68, 133.1, 128.5, 128.3, 127.8, 127.7, 126.9, 126.2, 126.1, 125.7, 113.0, 104.3, 81.6, 76.2, 72.3, 64.7, 64.6, 64.5, 30.8, 26.6, 16.3,16.2

^{31}P NMR (121MHz, $CDCl_3$): δ 6.52, 5.65, 4.90; ^{19}F NMR (282MHz, $CDCl_3$): δ -135.42, -135.60, -135.75, -135.92; LR MS (ESI) calcd for $C_{24}H_{31}F_2O_7PNa$ $[M + Na]^+$ *m/z* = 523.1643, found 523.1644.

Compound S21. Compound **S21** (9.00 g, 18.00 mmol) was dissolved in EtOAc (150.3 mL) and cooled to -15 ^{o}C (ethanol-dry ice bath). To this a solution of acetic anhydride (162 mL) acetic acid (125 mL) and concentrated H_2SO_4 (0.75 mL) in EtOAc (293 mL) was added. The reaction mixture was stirred at 6 ^{o}C (cold room) for 24h. The reaction mixture was washed with aqueous saturated $NaHCO_3$ (7 x 200 m), brine (200 mL), dried (Na_2SO_4), filtered and concentrated under reduced pressure. The residue was purified by silica gel column chromatography and eluted with 50% EtOAc in hexane to yield **S21** (4.67 g, 48%). ^{1}H NMR (300MHz, $CDCl_3$): δ 7.92 - 7.71 (m, 4H), 7.59 - 7.37 (m, 3H), 6.24 -5.59 (m, 2H), 5.33 (d, *J*=4.3 Hz, 1H), 4.89 - 4.72 (m, 1H), 4.72 - 4.56 (m, 1H), 4.56 - 4.46 (m, 1H), 4.39 - 4.16 (m, 7H), 4.07 (dd, *J*=4.3, 7.5 Hz, 1H), 2.13 (s, 3H), 2.06 (s, 3H), 1.45 - 1.36 (m, 6H); LR MS (ESI) calcd for $C_{25}H_{31}F_2O_9PNa$ 567.5, found 567.1.

Compound S22. Compound **S22** (3.2 g, 59%) was synthesized form compound **S21** (4.50 g, 8.27 mmol), thymine (2.07 g, 16.54 mmol) BSA (15 mL), anhydrous acetonitrile (80 mL for sugar, 15 mL for thymine) tin tetrachloride (1.06 mL, 9.10 mmol) following the same procedure used for the synthesis of compound **S15**. ^{1}H NMR (300MHz, DMSO-d_6): δ 11.43 (s, 1H), 7.98 - 7.84 (m, 4H), 7.64 - 7.43 (m, 4H), 5.84 (d, *J*=4.0 Hz, 1H), 5.45 (dd, *J*=4.1, 5.7 Hz, 1H), 4.72 (s, 2H), 4.41 - 4.10 (m, 6H), 2.17 - 2.03 (m, 4H), 1.87 - 1.69 (m, 4H), 1.49 - 1.11 (m, 6H); ^{19}F NMR (282MHz, DMSO-d_6): δ -136.00, -136.17, -136.32, -136.49; ^{31}P NMR (121MHz, $CDCl_3$): δ =

7.24, 6.37, 5.51; HR MS (ESI) calcd for $C_{28}H_{33}F_2N_2O_9PNa$ $[M + Na]^+$ *m/z* = 633.1742, found 633.1741.

Compound S23. Compound **S22** (1.05 g, 1.72 mmol) was dissolved in anhydrous DMF (9.3 mL). To this was added DBU (0.77 mL, 5.13 mmol). The reaction mixture was cooled in an ice bath and benzyl chloromethyl ether (0.35 mL, 2.57mmol) was added, and resulting reaction mixture was stirred at 0 ºC for 1 h. The reaction mixture was diluted with EtOAc (200 mL), washed with saturated aqueous $NaHCO_3$ (200 mL) and brine (200 mL) then dried (Na_2SO_4), filtered and evaporated. The residue obtained was dissolved in methanol (9.8 mL) and K_2CO_3 (0.83 g, 1.83 mmol). The reaction mixture was stirred at room temperature for 1 h. The mixture was poured into EtOAc (200 mL), washed with water (200 mL) and brine (200 mL), dried over anhydrous Na_2SO_4, filtered and evaporated. The residue was purified by silica gel column chromatography and eluted with 5% methanol in CH_2Cl_2. The residue (0.84 g, 1.22 mmol) after drying over P_2O_5 under reduced pressure overnight, was dissolved in anhydrous DMF (7 mL). The reaction mixture was cooled in an ice bath (0 ^oC) and NaH (60 % dispersion in mineral oil, 0.018 g, 1.22 mmol) was added and stirred for 30 min under argon atmosphere. To this methyl iodide (0.18 mL, 2.92 mmol) was added and stirring continued at 0 ^oC for 2 h. Acetic acid (1 mL) was added and diluted the reaction mixture with EtOAc (30 mL) and washed with water (30 mL), brine (25 mL), dried (Na_2SO_4), filtered and concentrated under reduced pressure. The residue was purified by silica gel column chromatography and eluted with 5% methanol in CH_2Cl_2 to yield **S23** (0.76 g, 63%). ^1H NMR (300MHz, $CDCl_3$): δ 7.96 - 7.73 (m, 5H), 7.56 - 7.44 (m, 3H), 7.40 - 7.26 (m, 7H), 5.71 (d, *J*=2.4 Hz, 1H), 5.47 (d, *J*=4.1 Hz, 2H), 4.72 - 4.66 (m, 2H), 4.56 (d, *J*=3.2 Hz, 1H), 4.35 - 4.19 (m, 4H), 3.98 (dd, *J*=2.6, 5.1 Hz, 1H), 3.85 (dd, *J*=5.3, 7.3 Hz, 1H), 3.54 - 3.49 (m, 3H), 2.72 - 2.22 (m, 2H), 1.95 - 1.84 (m, 4H), 1.41 - 1.32 (m, 7H);

^{13}C NMR (75MHz, $CDCl_3$): δ 163.3, 150.5, 137.9, 135.1, 134.6, 133.2, 113.1 128.37, 128.4, 127.96, 127.9, 127.7, 126.9, 126.3, 126.2, 125.7, 110.4, 91.9, 80.3, 79.6, 72.8, 72.3, 70.5, 64.8, 64.7, 60.4, 58.5, 21.0, 16.4, 16.3, , 13.24; ^{19}F NMR (282MHz, $CDCl_3$): δ -109.15 to -112.17 (m); ^{31}P NMR (121MHz, $CDCl_3$): δ 7.08, 6.20, 5.35; LR MS (ESI) calcd for $C_{35}H_{41}F_2N_2O_9PNa$ $[M + Na]^+$ *m*/*z* = 725.7, found 725.2.

Compound S24. Compound **S23** (0.42 g, 0.60 mmol) was dissolved in methanol(8.3 mL) and Pd/C (20 wt% of palladium on carbon dry base, 0.17 g). Flushed the reaction mixture with H_2 gas and stirred at room temperature under H_2 gas for 6 h. Catalyst was filtered through a pad of celite, washed throughly with methanol. The filtrate and washings were pooled together, triethylamine (0.6 mL) was added and the mixture was stirred at room temperature for 2 h. Solvent was removed under reduced pressure and the residue purified by silica gel column chromatography and eluted with 5% methanol in dichloromethane to yield **S24** (0.22 g, 85%). ^1H NMR (300MHz, DMSO-d_6): δ 11.37 (s, 1H), 7.47 (s, 1H), 5.77 (d, *J*=4.3 Hz, 1H), 5.35 (d, *J*=5.8 Hz, 1H), 4.21 (quin, *J*=7.3 Hz, 4H), 4.10 - 3.99 (m, 2H), 3.95-3.86 (m, 1H), 3.36 (s, 3H), 1.95 - 1.60 (m, 1H), 2.60-2.35 (m, 1H), 1.79 (s, 3H), 1.29 (t, *J*=7.1 Hz, 6H); ^{19}F NMR (282MHz, DMSO-d_6): δ -107.92 - -112.11 (m); ^{31}P NMR (121MHz, $CDCl_3$): δ 7.24 (s, 1P), 6.38 (s, 2P), 5.51 (s, 1P); HR MS (ESI) calcd for $C_{16}H_{26}F_2N_2O_8P$ $[M - H]^-$ *m*/*z* = 443.1371, found 443.1372.

Compound 46. The compound **46** (0.12 g, 70%) was synthesized from compound **S24** (0.12 g, 0.26 mmol), DMF (1 ml), 1H-tetrazole (0.016 g, 0.23 mmol), *N*-methylimidazole (0.008 mL , 0.10 mmol) and 2-cynoethyl-*N*,*N*,*N*',*N*'-tetraisopropylphosphordiamidite (0.13 ml, 0.39 mmol) following the same procedure used for the synthesis of compound **30b**. ^{31}P NMR (121MHz,

$CDCl_3$): δ 150.91, 150.21, 7.26, 7.18, 6.40, 6.32, 5.53, 5.45; HR MS (ESI) calcd for $C_{25}H_{41}F_2N_4O_9P_2$ $[M - H]^-$ m/z = 641.3383, found 641.3385.

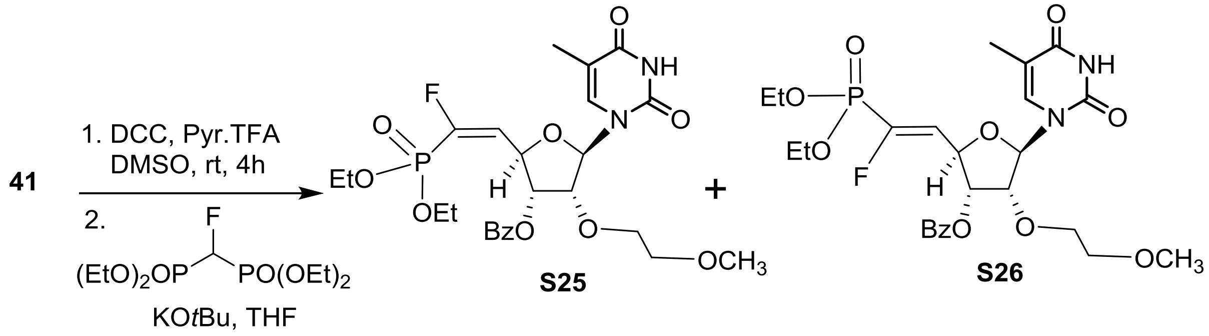

Scheme S6. Synthesis of compound **S25** and **S26**

Compound S25 and S26. DCC (2.7 mmol, 0.56 g, *Caution – Extreme irritant and sensitizer. Avoid all contact of equipment used for weighing and transferring DCC with skin. Dispose of gloves immediately after handling DCC*) was added to stirred a solution of **41** (2.1 mmol, 0.89 g) and pyridinium trifluoroacetate (1.1 mmol, 0.2 g) in DMSO (10 mL) and the reaction was stirred at room temperature for three hours. In a separate flask, potassium *tert*-butoxide (3.3 mL of a 1M soultion in *t*-BuOH) was added to a cold (0 ^{o}C) solution of tetraethylfluoromethyldiphosphonate (3.2 mmol, 0.98 g) in THF (10 mL). After stirring for 10 minutes, the THF solution was cannulated into the reaction and the stirring was continued for another 10 minutes. The reaction was diluted ethyl acetate and the organic phase was washed with water, saturated sodium bicarbonate, brine, dried (Na_2SO_4) and concentrated. Purification by chromatography (silica gel, eluting with 0 to 30% acetone in dichloromethane) provided partially pure **S26** (0.84 g, less polar spot, R_f = 0.4 40% acetone in dichloromethane) and partially pure **S25** (0.89 g, more polar spot, R_f = 0.3 40% acetone in dichloromethane). **S25** ^{1}H NMR (300 MHz, $CDCl_3$): δ 9.05 (br s, 1H), 8.08 (d, *J*=7.7 Hz, 2H), 7.67–7.55 (m, 1H), 7.53–7.42 (m, 2H), 7.13 (s, 1H), 6.22 (td, *J*=7.5, 37.3

Hz, 1H), 5.91 (d, *J*=4.0 Hz, 1H), 5.35–5.18 (m, 2H), 4.53 (t, *J*=4.4 Hz, 1H), 4.29–4.08 (m, 4H), 3.85–3.63 (m, 2H), 3.48–3.35 (m, 2H), 3.23–3.12 (m, 3H), 1.97 (s, 3H), 1.37 (t, *J*=7.1 Hz, 3H), 1.30 (t, *J*=7.1 Hz, 3H). ^{13}C NMR (75 MHz, $CDCl_3$): δ 165.6, 163.7, 153.5, 150.1, 136.2, 133.6, 129.9, 129.1, 128.5, 121.2, 111.4, 90.9, 80.1, 74.5, 74.3, 72.1, 71.0, 63.7, 63.6, 58.9, 16.2, 12.6; ^{31}P NMR (121MHz, $CDCl_3$): δ 3.08 (d, *J*=96.8 Hz, 1P); ^{19}F NMR (282 MHz, $CDCl_3$): δ -122.23 (dd, *J*=36.7, 96.4 Hz, 1F). LR MS (ESI) calcd for $C_{25}H_{33}FN_2O_{10}P$ 571.5, found 571.1; LRMS-ESI calcd for $C_{25}H_{33}FN_2O_{10}P$ $[M + H]^+$ m/z = 571.2, found 571.1. **S26** ^{1}H NMR (300 MHz, $CDCl_3$): δ 9.01 (s, 1H), 8.12 (d, *J*=7.3 Hz, 2H), 7.65–7.53 (m, 1H), 7.51–7.40 (m, 2H), 7.10 (s, 1H), 6.30 (ddd, *J*=10.1, 25.6, 29.9 Hz, 1H), 5.86–5.75 (m, 1H), 5.70 (d, *J*=3.8 Hz, 1H), 5.35 (t, *J*=6.1 Hz, 1H), 4.66 (dd, *J*=4.1, 5.7 Hz, 1H), 4.30–3.97 (m, 4H), 3.79–3.62 (m, 2H), 3.48–3.32 (m, 2H), 3.17 (s, 3H), 1.96 (s, 3H), 1.36 (t, *J*=7.1 Hz, 3H), 1.20 (t, *J*=7.1 Hz, 3H). ^{13}C NMR (75 MHz, $CDCl_3$): δ 165.7, 163.8, 154.1, 149.9, 137.8, 133.48, 130.0, 129.3, 128.4, 123.5, 111.1, 93.1, 80.1, 75.6, 75.4, 75.1, 72.1, 71.1, 63.5, 58.9, 16.1, 12.5. ^{31}P NMR (121 MHz, $CDCl_3$): δ 2.07 (d, *J*=98.7 Hz, 1P). ^{19}F NMR (282 MHz, $CDCl_3$): δ -113.10 (dd, *J*=26.0, 99.8 Hz, 1F). LR MS (ESI) calcd for $C_{25}H_{33}FN_2O_{10}P$ $[M + H]^+$ m/z = 571.5, found 571.1.

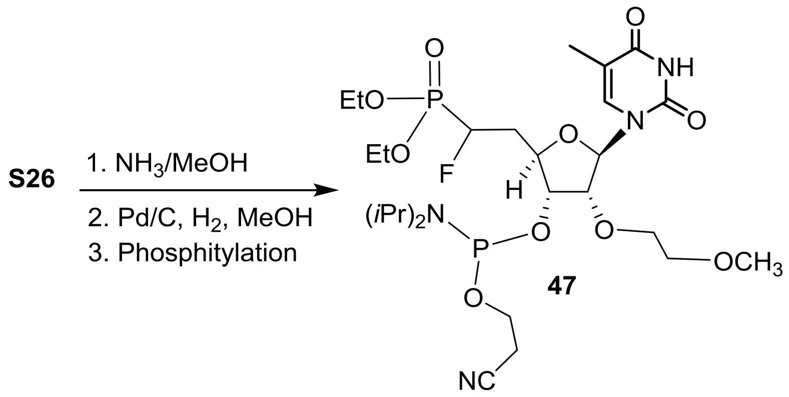

Scheme S7. Synthesis of compound **S47**

Compound 47. A solution of mixture of **S26** (0.2 g) in methanolic ammonia (5 mL of a 7N solution) was aged at room temperature for 8 hours after which the solvent was evaporated under reduced pressure. The residue was purified by chromatography (silica gel, eluting with 20 to 75% acetone in dichloromethane) to yield 3'-hydroxy derivative (0.18 g). A solution of 3'-hydroxyderivative (0.18 g, 0.39 mmol) was hydrogenated using palladium on carbon (10% w/w, 20 mg) in methanol (4 mL) using a hydrogen balloon for 10 h. The reaction was filtered through celite and the filtrate was concentrated under reduced pressure. Purification by chromatography (silica gel, eluting with 20 to 75% acetone in dichloromethane) provided the saturated nucleoside (0.15 g, 86%) which was dried under high vacuum. ^{31}P NMR (121 MHz, $CDCl_3$): δ 17.57 (d, *J*=71.4 Hz, 1P), 17.19 (d, *J*=73.8 Hz, 1P). ^{19}F NMR (282 MHz, $CDCl_3$): δ -207.33 (m, 1F), -211.38 (m, 1F). LR MS (ESI) calcd for $C_{18}H_{30}FN_2O_9P$ 469.2, found 469.1. 2-Cyanoethyl tetraisopropylphosphordiamidite (0.14 mL, 0.47 mmol) was added to a solution of the nucleoside from above (0.15 g, 0.31 mmol), 1*H*-tetrazole (18 mg, 0.25 mmol) and *N*-methylimidazole (1 drops) in DMF (1.5 mL). After stirring at room temperature for 4 h the reaction was diluted EtOAc and the organic layer was washed with brine, dried (Na_2SO_4) and concentrated. Purification by chromatography (silica gel, eluting with 30% acetone in dichloromethane) provided **47** (0.14 g, 70%) as a white solid. ^{31}P NMR (121 MHz, $CDCl_3$): δ 150.19 (s, 1P), 150.07 (s, 1P), 17.37 (d, *J*=73.8 Hz, 1P), 17.06 (d, *J*=73.8 Hz, 1P). ^{19}F NMR (282 MHz, $CDCl_3$): δ -206.98 (m, 1F), -211.37 (m, 1F). LR MS (ESI) calcd for $C_{27}H_{46}FN_4O_{10}P_2$ $[M - H]^-$ *m/z* = 667.3725, found 667.3727.

Compound S27. Compound[2] **40** (24 g, 38.79 mmol) and imidazole (15.84 g, 232.74 mmol) was dissolved in anhydrous DMF (50 mL). To this TBDPSCl (30.3 mL, 116.37 mmol) was added and stirred the resulting mixture at room temperature for 18 h under argon atmosphere. The

reaction mixture was diluted with EtOAc (500 mL) and the organic phase was washed with saturated $NaHCO_3$ (500 mL), brine (500 mL), dried (Na_2SO_4) and concentrated. Purification by chromatography (silica gel, eluting with 50% EtOAc in hexane) provided 3'-*O*-TBDPS analog of compound **40** (25.9 g, 78%). 3'-*O*-TBDPS derivative of compound **40** (13.13 g, 17.92 mmol) was dried over P_2O_5 under reduced pressure and then dissolved in anhydrous DMF (99 mL). The resulting solution was cooled in an ice bath (0 ^{o}C) and 1,8-diazabicyclo-[5-4-0]undec-7ene (DBU, 8.04 mL, 53.76 mmol) was added. The reaction mixture was stirred at 0 ^{o}C for 10 min under argon atmosphere. To this benzylchloromethyl ether (3.73 mL, 26.88 mmol) wad added. The reaction mixture was removed from the ice bath and allowed to stirrer at room temperature for 14 h and then poured into aqueous saturated $NaHCO_3$ solution (800 mL). The white precipitate formed was collected by filtration. The solid thus obtained was washed thoroughly with water and dried under reduced pressure and dissolved in CH_3OH/acetic acid/water (4:1:3, 132 mL) and stirred at room temperature for 18 h. The reaction mixture was diluted with EtOAc (300 mL) the organic phase was washed with water (400 mL), aqueous saturated sodium $NaHCO_3$ (300 mL), brine (300 mL), dried (Na_2SO_4) and concentrated. The residue was purified by silica gel column chromatography and eluted with 0-5% methanol in dichloromethane to yield **S27** (8.23 g, 83.5%). ^{1}H NMR (300MHz, DMSO-d$_6$): δ 7.89 (d, *J*=1.1 Hz, 1H), 7.39 - 7.23 (m, 5H), 5.86 (d, *J*=4.9 Hz, 1H), 5.43 - 5.32 (m, 2H), 5.32 - 5.20 (m, 1H), 4.64 - 4.52 (m, 2H), 4.28 (t, *J*=4.8 Hz, 1H), 4.08 - 3.94 (m, 1H), 3.86 (d, *J*=4.3 Hz, 1H), 3.77 - 3.48 (m, 4H), 3.41 (t, *J*=5.0 Hz, 2H), 3.18 (s, 3H), 1.83 (s, 3H), 0.88 (s, 9H), 0.15 (s, 3H), 0.12 (s, 3H); ^{13}C NMR (75MHz, $CDCl_3$): δ 163.4, 151.0, 137.9, 137.1, 128.3, 128.0, 127.7, 127.6, 109.9, 92.5, 85.4, 81.0, 72.2, 71.9, 70.4, 70.0, 69.9, 61.3, 58.9, 25.7, 18.1, 13.1, -4.7, -5.0; LR MS (ESI) calcd for $C_{27}H_{43}N_2O_8Si$ $[M + H]^+$ m/z = 551.2758, found 551.2770.

Compound S28. To a solution of compound **S27** (2.17 g, 3.95 mmol) in anhydrous DMF (13 mL) NaH (60% dispersion in mineral oil, 0.24 g, 5.93 mmol) was added. The reaction mixture was cooled to -10 ^{o}C (dry ice/acetone)and diethyliodomethylphophonate (1.65 g, 5.93 mmol) was added. The reaction mixture was stirred at -10 ^{o}C for 3 h and ethanol (1 mL) was added to quench the reaction. The reaction mixture was diluted with EtOAc (100 mL) and the organic phase was washed with water (100 mL), brine (100 mL), dried (Na_2SO_4) and concentrated. Purification by chromatography (silica gel, eluting with 50% EtOAc in hexane followed by 5% methanol in dichloromethane) to provided **S28** (1.62 g, 58.7%). ^{1}H NMR (300MHz, $CDCl_3$): δ 7.49 (d, *J*=1.1 Hz, 1H), 7.42 - 7.32 (m, 5H), 6.02 (d, *J*=4.0 Hz, 1H), 5.50 (d, *J*=1.9 Hz, 2H), 4.93 (s, 1H), 4.76 - 4.59 (m, 2H), 4.29 - 3.93 (m, 8H), 3.93 - 3.72 (m, 4H), 3.66 - 3.47 (m, 2H), 3.31 (s, 3H), 1.93 (s, 3H), 1.46 - 1.30 (m, 6H), 0.95 (m, 9H), 0.15 (s, 3H), 0.13 (s, 3H); ^{31}P NMR (121MHz, $CDCl_3$): δ 20.4; LR MS (ESI) calcd for $C_{32}H_{54}N_2O_{11}PSi$ $[M + H]^+$ m/z = 701.8, found 701.3.

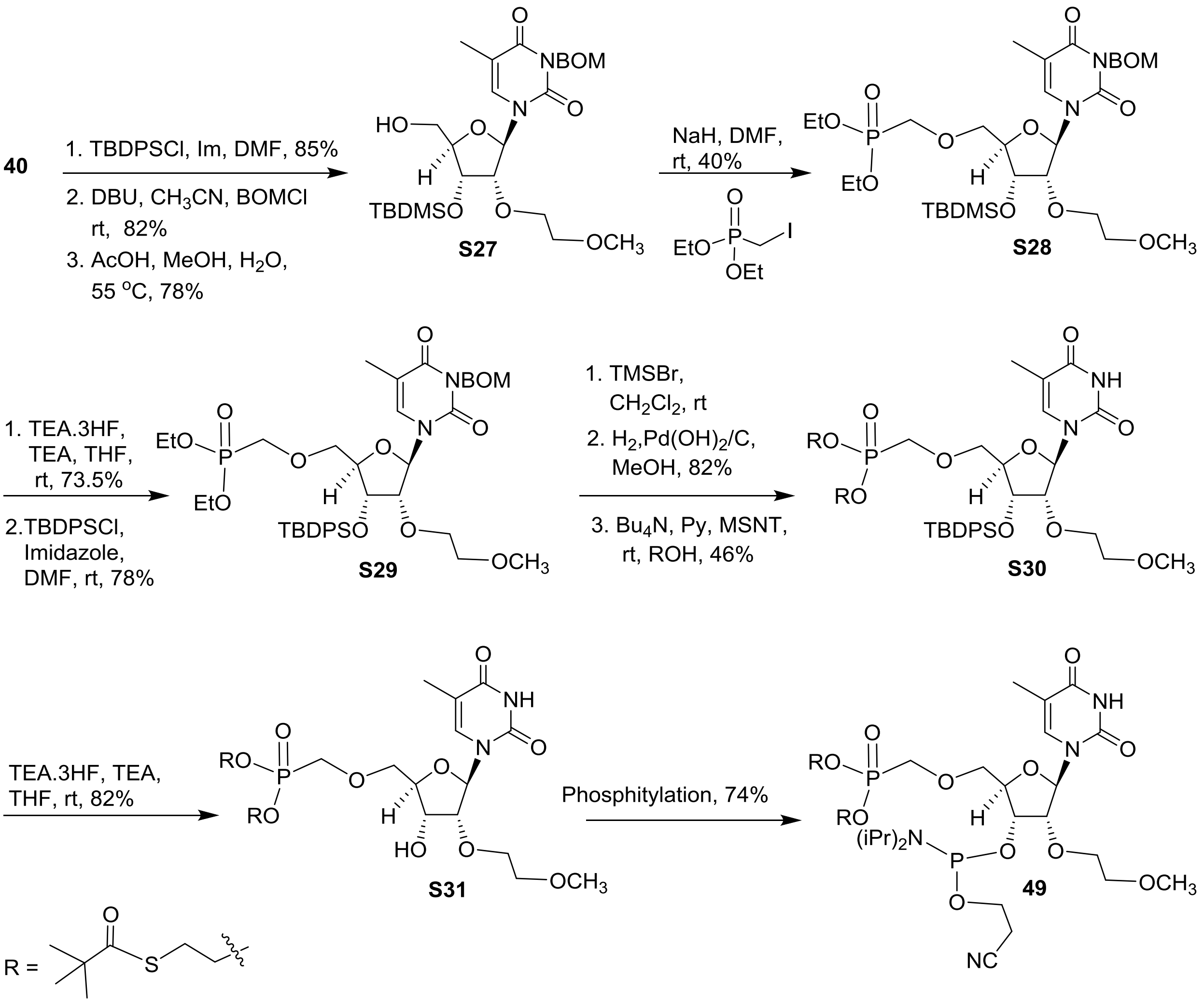

Scheme S8. Synthesis of compound **49**

Compound S29. To a solution of compound **S28** (1.62 g, 2.32 mmol) in THF (20 mL) triethylamine (0.80 mL, 5.78 mmol) and triethylamine trihydrofluoride (1.88 mL, 11.56 mmol) were added. After stirring for 18 h at room temperature the reaction mixture was diluted with EtOAc (50 mL). The organic phase thus obtained was washed with water (50 mL), aqueous saturated sodium bicarbonate (50 mL), brine (50 mL), dried (Na_2SO_4), filtered and concentrated. The residue was purified by silica gel column chromatography and eluted first with 50% EtOAc in hexane and then with 5% methanol in dichloromethane to yield 1.16 g (73.5%) of 3’-

hydroxyderivative. HR MS (ESI) calcd for $C_{26}H_{40}N_2O_{11}P$ $[M + H]^+$ m/z = 587.2332, found 587.2332. The 3'-hydroxyderivative (0.66 g, 1.12 mmol) and imidazole (0.46 g, 6.72 mmol) was dissolved in anhydrous DMF (1.4 mL). To this TBDPSCl (0.92 g, 3.36 mmol) was added and stirred the resulting mixture at room temperature for 18 h under argon atmosphere. The reaction mixture was diluted with EtOAc (50 mL) and the organic phase was washed with saturated $NaHCO_3$ (50 mL), brine (50 mL), dried (Na_2SO_4) and concentrated. Purification by chromatography (silica gel, eluting with 5% methanol in dichloromethane) provided compound **S29** (0.79 g, 85%). ^1H NMR (300MHz, DMSO-d_6): δ 7.74 - 7.56 (m, 4H), 7.54 - 7.37 (m, 7H), 7.38 - 7.19 (m, 5H), 5.94 (d, *J*=4.1 Hz, 1H), 5.45 - 5.27 (m, 2H), 4.82 (s, 1H), 4.63 - 4.50 (m, 2H), 4.26 (d, *J*=3.8 Hz, 2H), 4.19 - 3.79 (m, 8H), 3.71 (q, *J*=4.5 Hz, 2H), 3.50 - 3.39 (m, 2H), 3.19 (s, 3H), 1.47 (s, 3H), 1.22 (q, *J*=7.0 Hz, 6H), 1.04 (s, 9H); ^{13}C NMR (75MHz, $CDCl_3$): δ 163.3, 151.0, 138.0, 135.5, 135.2, 133.9, 132.9, 132.3, 130.1, 130.0, 128.3, 128.2, 128.0, 127.9, 127.6, 127.6, 110.4, 87.6, 82.6, 81.6, 78.0, 77.9, 72.2, 72.1, 70.5, 70.1, 65.6, 63.4, 63.3, 62.5, 62.5, 62.4, 58.9, 27.0, 19.4, 16.4, 12.5; ^{31}P NMR (121MHz, $CDCl_3$): δ 20.4; LR MS (ESI) calcd for $C_{42}H_{58}N_2O_{11}PSi$ $[M + H]^+$ m/z = 826.0, found 825.3.

Compound S30. Compound **S29** (0.79 g, 0.96 mmol) was dissolved in anhydrous dichloromethane (48 mL) and bromotrimethylsilane (2.63 mL, 20.34 mmol) was added. The reaction mixture was stirred at room temperature for 18 h. Solvent was removed under reduced pressure and the residue obtained was co-evaporated with methanol (5 mL). The residue was dissolved in methanol (5 mL) and triethylamine (1 mL) was added. Concentrated under reduced pressure and the residue was dissolved in methanol and co-evaporated with toluene. The residue dissolved in methanol (30 mL) and palladium hydroxide (20 wt% Pd on carbon base, 1.8 g) was added. Flushed the reaction mixture with H_2 gas and stirred the reaction mixture under H_2 gas

(balloon) at room temperature for 18 h. The reaction mixture was filtered through a pad of celite. The elite pad was washed thoroughly with methanol. Combined the filtrate and washing and concentrated under reduced pressure. The residue obtained was dissolved in methanol (25 mL) and tributylamine (1.38 mL, 5.78 mmol) was added. After stirring for 5 min solvent was removed under reduced pressure. The residue thus obtained was co-evaporated with toluene (3 x 30 mL). The residue was dissolved in anhydrous pyridine (28 mL) and S-*tert*-butyl-2-hydroxythioethanol (5.72 g, 35.30 mmol) and 1-(2-mesitylenesulfonyl)-3-nitro-1,2,4-triazole (MSNT, 2.24 g, 7.55 mmol) were added. The reaction mixture was stirred at room temperature for 48 h under argon atmosphere. Aqueous saturated sodium bicarbonate solution (200 mL) and the solution was extracted with EtOAc (3 x 100 mL). Organic phase was washed with brine (300 mL), dried (Na_2SO_4) and concentrated. The residue was purified by silica gel column chromatography and eluted first with 50% EtOAc in hexane and then with 0-5% methanol in dichloromethane to yield **S30** (0.50 g, 54%). ^{1}H NMR (300MHz, DMSO-d$_6$): δ 7.68-7.62 (m, 4H), 7.52 - 7.40 (m, 6H), 7.35 (s, 1H), 5.89 (d, *J*=5.3 Hz, 1H), 4.25 (d, *J*=2.3 Hz, 2H), 4.16 - 3.97 (m, 8H), 3.97 - 3.79 (m, 2H), 3.77 - 3.61 (m, 2H), 3.50 - 3.41 (m, 2H), 3.20 (s, 3H), 3.10 (q, *J*=6.5 Hz, 4H), 1.43 (s, 3H), 1.17 (s, 9H), 1.16 (s, 9H), 1.04 (s, 9H); ^{13}C NMR (75MHz, $CDCl_3$): δ 164.0, 150.6, 135.7, 135.4, 133.1, 132.4, 130.4, 130.2, 128.2, 128.1, 111.3, 87.0, 82.9, 82.0, 78.6, 78.4, 72.4, 70.4, , 65.1, 65.0, 63.7, 63.4, 59.1, 46.6, 29.0, 27.4, 27.2, 19.6, 12.5; ^{31}P NMR (121MHz, $CDCl_3$): δ 21.0; LR MS (ESI) calcd for $C_{44}H_{65}N_2O_{12}PS_2SiNa$ $[M + Na]^+$ *m*/*z* = 959.3319, found 959.3321.

Compound S31. To a solution of compound **S30** (0.43 g, 0.46 mmol) in THF (5.2 mL) triethylamine (0.32 mL, 2.3 mmol) and triethylamine trihydrofluoride (0.75 mL, 4.6 mmol) were added. After stirring for 18 h at room temperature the reaction mixture was diluted with EtOAc (

50 mL). The organic phase thus obtained was washed with water (50 mL), aqueous saturated sodium bicarbonate (50 mL), brine (50 mL), dried (Na_2SO_4), filtered and concentrated. The residue was purified by silica gel column chromatography and eluted first with 50% EtOAc in hexane and then with 5% methanol in dichloromethane to yield **S31** (0.24 g, 75%). ^{1}H NMR (300MHz, $CDCl_3$): δ 8.62 (br. s, 1H), 7.80 (d, *J*=1.1 Hz, 1H), 5.74 (d, *J*=2.3 Hz, 1H), 4.38 - 4.06 (m, 10H), 4.06 - 3.67 (m, 7H), 3.57 (dt, *J*=2.2, 3.5 Hz, 2H), 3.35 (s, 3H), 3.20 - 3.11 (m, 4H), 1.92 (m, 3H), 1.24 (s, 9H), 1.23 (s, 9H); ^{13}C NMR (75MHz, $CDCl_3$): δ 205.8, 163.6, 150.1, 137.2, 110.4, 90.3, 82.6, 80.2, 72.1, 70.0, 65.2, 64.9, 64.8, 63.1, 62.0, 60.5, 59.0, 46.6, 28.8, 28.7, 27.3,12.44; ^{31}P NMR (121MHz, $CDCl_3$): δ 21.37; LR MS (ESI) calcd for $C_{28}H_{47}N_2O_{12}PS_2Na$ [M + Na]$^+$ *m/z* = 721.2159, found 721.2159.

Compound 49. The compound **49** (0.14 g, 70%) was synthesized from compound **S31** (0.14 g, 0.2 mmol), DMF (0.8 ml), 1H-tetrazole (0.012 g, 0.17 mmol), *N*-methylimidazole (0.006 mL , 0.066 mmol) and 2-cynoethyl-*N*,*N*,*N*',*N*'-tetraisopropylphosphordiamidite (0.10 ml, 0.31 mmol) following the same procedure used for the synthesis of compound **30b**. ^{31}P NMR (121MHz, $CDCl_3$): δ 149.88, 147.82, 20.69, 20.58; LR MS (ESI) calcd for $C_{37}H_{63}N_4O_{13}P2S_2$ [M - H]$^-$ *m/z* = 898.0, found 897.3.

Compound 51. Compound **40** (15 g, 24.27 mmol) was dissolved in anhydrous DMF (100 ml). and cooled in an ice bath (0 ^{o}C). To this DBU (5.13 mL, 36.41 mmol) was added. The reaction mixture was stirred at 0 ^{o}C for 10 min under argon atmosphere. To this benzylchloromethyl ether (5.05 mL, 36.41 mmol) wad added. The reaction mixture was removed from the ice bath and allowed to stirrer at room temperature for 4 h and then poured into water (800 mL). The aqueous solution was extracted with EtOAc (3 x 300 mL). The organic phase dried (Na_2SO_4),

filtered and concentrated under reduced pressure. The residue was purified by silica gel column chromatography and eluted with 20-70% EtOAc in hexane to get 3-*N*-benzyloxymethyl (BOM) analog of compound **40** (22 g, 29.81 mmol). It was dissolved in anhydrous DMF (100 mL) and cooled the reaction mixture in an ice bath (0 ^{o}C). To this NaH (60% dispersion in mineral oil, 1.79 g, 44.71 mmol) was added and stirred at 0 ^{o}C for 30 min under argon atmosphere. 2-(Bromomethyl)naphthalene (Nap-Br, 9.87 g, 44.71 mmol) was added and stirring continued at 0 ^{o}C for additional 1 h. To this reaction mixture water (800 mL) and aqueous layer thus obtained was extracted with EtOAc (3 x 300 mL). The organic phase dried (Na_2SO_4), filtered and concentrated under reduced pressure. The residue was purified by silica gel column chromatography and eluted with 20-50% EtOAc in hexane to get 3'-*O*-((naphthalene-2-yl)methyl)-3-*N*-benzyloxymethyl analog of compound **40**. It was then dissolved in dichloromethane (100 mL) and trifluoroacetic acid (4.4 mL, 56.64 mmol) and triethylsilane (9.5 mL, 59.62 mmol) were added and stirred at room temperature for 2 h. The solvent was removed under reduced pressure. The residue obtained was partitioned between EtOAc (400 mL) and water (300 mL). Organic phase separated and washed with aqueous saturated sodium bicarbonate solution (300 mL), brine (300 mL), dried (Na_2SO_4), filtered and concentrated under reduced pressure. The residue obtained was purified by silica gel column chromatography and eluted with 0-5% methanol in dichloromethane to yield **51** (14.23 g, 69.4%). ^{1}H NMR (300MHz, DMSO-d$_6$) δ 8.02 - 7.85 (m, 4H), 7.59 - 7.47 (m, 4H), 7.41 - 7.21 (m, 5H), 5.92 (d, *J*=4.0 Hz, 1H), 5.46 - 5.24 (m, 3H), 4.89 - 4.68 (m, 2H), 4.60 (s, 2H), 4.33 - 4.06 (m, 3H), 3.85 - 3.55 (m, 4H), 3.22 (s, 3H), 1.83 (s, 3H); LR MS (ESI) calcd for $[M + H]^+$ m/z = $C_{32}H_{37}N_2O_8$ 577.7, found 577.3.

Compound 52a. Compound **51** (1.51 g, 2.61 mmol) and 2,6-di-*tert*-butyl-4-methylpyridine (2.30 g, 11.23) were mixed together and dried over P_2O_5 under reduced pressure. The dried mixture was dissolved in anhydrous dichloromethane (17 mL) and the reaction mixture was cooled to -20 ^oC (dry ice/acetone bath) under argon atmosphere. To this trifluoromethanesulfonic anhydride (0.67 mL, 3.93 mmol) was added and the reaction mixture was stirred at -20 ^oC for 1 h. Reaction mixture was diluted with EtOAc (100 mL) and washed with ice cold water (100 mL). The organic phase dried (Na_2SO_4), filtered and evaporated to yield 5'-trifluromethanesulfonyl derivative. In a separate flask tetraethyl methylenebis(phosphonate) (1.13 g, 3.93 mmol) was dissolved in anhydrous THF (11 mL) and cooled to 0 ^oC. To this 1 M potassium *tert*-butoxide in THF (3.93 mL, 3.93 mmol) was added and stirred at 0 ^oC for 30 min under argon atmosphere. To this a solution of 5'-trifluormethanesufonyl derivative of compound **51** was added in anhydrous THF (6 mL). After stirring for additional 2 h at 0 ^oC the reaction mixture was diluted with EtOAc (100 mL) and washed with water (100 mL). The organic phase dried (Na_2SO_4), filtered and concentrated. The residue obtained was purified by silica gel column chromatography and eluted with first with EtO Ac, then with 5% methanol in dicloromethane to yield **52**a (1.72 g, 78%). ^{1}H NMR (300MHz, DMSO-d_6): δ 8.01 - 7.82 (m, 4H), 7.61 - 7.44 (m, 4H), 7.41 - 7.21 (m, 5H), 5.87 (d, *J*=4.0 Hz, 1H), 5.36 (s, 2H), 4.91 - 4.68 (m, 2H), 4.60 (s, 2H), 4.31 (t, *J*=4.6 Hz, 2H), 4.14 - 3.93 (m, 10H), 3.72 (td, *J*=4.6, 9.1 Hz, 2H), 3.56 - 3.43 (m, 2H), 3.22 (s, 3H), 1.86 (s, 3H), 1.29 - 1.11 (m, 14H); ^{31}P NMR (121MHz, DMSO-d_6): δ 23.35, 23.05 - 13.46; LR MS (ESI) calcd for $C_{41}H_{57}N_2O_{13}P_2$ $[M + H]^+$ m/z = 847.9, found 847.6.

Compound 52b. To a sloution of compound **52a** (1.65 g, 1.95 mmol) in CH_2Cl_2 (14 mL) DDQ (0.70 g, 3.08 mmol) and water (0.055 g, 3.08 mmol) were added. The reaction mixture was stirred at room temperature for 3 h. The reaction mixture was diluted with CH_2Cl_2 (100 mL) and

washed with 5% aqueous sodium bisulfite solution (100 mL) and brine (100 mL), dried (Na_2SO_4), filtered and concentrated under reduced pressure. The residue obtained was purified by silica gel column chromatography and eluted with 5% methanol in CH_2Cl_2 and fractions were concentrated. The residue was dissolved in MeOH (20 ml) and palladium hydroxide (0.6 g, 20 wt% Pd on carbon dry base) was added. The reaction mixture was flushed with H_2 gas and stirred at room temperature under H_2 atmosphere for 16 h. The reaction mixture was filtrated through a pad of celite and washed the celite pad thoroughly with EtOAc. Combined filtrate and the washings and concentrated under reduced pressure. The residue was then dissolved in MeOH (20 mL) and triethylamine (1 mL was added) and stirred for 3 h at room temperature. The reaction mixture was concentrated under reduced pressure and purified by silica gel column chromatography and eluted with 5-10% CH_3OH to yield **52b**. ^{1}H NMR (300MHz, DMSO-d_6): δ 11.36 (s, 1H), 7.46 (s, 1H), 5.79 (d, *J*=4.9 Hz, 1H), 5.11 (d, *J*=5.7 Hz, 1H), 4.15 - 3.92 (m, 12H), 3.77 - 3.59 (m, 2H), 3.51 - 3.36 (m, 2H), 3.21 (s, 3H), 1.80 (s, 3H), 1.24 (q, *J*=7.2 Hz, 14H); ^{31}P NMR (121MHz, DMSO-d_6): δ 23.64, 23.11; LR MS (ESI) calcd for $C_{22}H_{41}N_2O_{12}P_2$ $[M + H]^+$ *m/z* = 587.5, found 587.2.

Compound 53. The compound **53** (0.55 g, 75.3%) was synthesized from compound **52b** (0.55 g, 0.94 mmol), DMF (3 mL), 1H-tetrazole (0.057 g, 0.82 mmol), *N*-methylimidazole (0.020 mL , 0.26 mmol) and 2-cynoethyl-*N*,*N*,*N*',*N*'-tetraisopropylphosphordiamidite (0.48 mL, 1.53 mmol) following the same procedure used for the synthesis of compound **30b**. ^{31}P NMR (121MHz, DMSO-d_6): δ 149.15, 149.02, 23.44, 23.32, 23.02, 22.75; HR MS (ESI) calcd for $C_{31}H_{56}N_4O_{13}P_3$ $[M - H]^-$ *m/z* = 785.4173, found 785.4176.

Compound S32 Oxalyl chloride (34.0 mmol, 2.90 mL) was added drop-wise to a cold (-78 ^{o}C) solution of DMSO (68 mmol, 4.80 mL) in dichloromethane (200 mL). After stirring for 30 min,

a solution of the alcohol **S19** (24 mmol, 7.9 g) in dichloromethane (30 mL) was added over 5 minutes *via* a cannula. The reaction was stirred for another 30 min after which triethylamine (72 mmol, 9.4 mL) was added and the reaction was removed from the ice bath. After stirring for another 45 min, the starting alcohol was completely consumed as analyzed by TLC. The reaction was poured into a separatory funnel and the organic layer was sequentially washed with 5% aqueous HCl, aqueous saturated $NaHCO_3$ solution, brine, dried (Na_2SO_4) and concentrated to provide the aldehyde which was used without any further purification.

Triphenyl phosphine (91.5 mmol, 24.0 g) was added in portions to a cold (0 ^oC) solution of carbon tetrabromide (45.7 mmol, 15.2 g) in dichloromethane (220 mL). The reaction was stirred for 20 minutes after which a solution of the aldehyde from above in dichloromethane (50 mL) was added *via* a cannula. The reaction was stirred for another 4 h and the organic layer was washed with saturated NH_4Cl solution, brine, dried (Na_2SO_4) and concentrated. The white solid thus formed was suspended in a mixture of hexanes and EtOAc and filtered to remove the triphenylphosphine oxide. Purification by column chromatography (silica gel, eluting with 0 to 25% ethyl acetate in hexanes) provided **S32** (6.1 g, 58% from S19). ^1H NMR (300MHz, $CDCl_3$): δ 7.99 - 7.75 (m, 4H), 7.61 - 7.41 (m, 3H), 6.28 (d, *J*=8.5 Hz, 1H), 5.67 (d, *J*=3.6 Hz, 1H), 5.02 - 4.84 (m, 1H), 4.84 - 4.73 (m, 2H), 4.57 (t, *J*=3.9 Hz, 1H), 3.62 (dd, *J*=4.1, 8.9 Hz, 1H), 1.65 (s, 3H), 1.37 (s, 3H); LR MS (ESI) calcd for $C_{20}H_{20}Br_2O_4Na$ $[M + Na]^+$ m/z = 507.2, found 506.9.

Compound S33. Concentrated sulfuric acid (5 drops) was added to a solution of **S32** (13.0 mmol, 6.6 g) in glacial acetic acid (39 mL) and acetic anhydride (8 mL). The reaction was stirred at room temperature for 2 hours after which the acetic acid was removed at room temperature under high vacuum on a rotary evaporator. The residue was dissolved in ethyl acetate and the organic layer was washed with water, saturated sodium bicarbonate (caution – effervescence),

brine, dried (Na_2SO_4) and concentrated to provide a mixture of anomeric diacetates which was used without any further purification. *N*,*O*-Bis-trimethylsilylamide (42.9 mmol, 10.6 mL) was added to a suspension of thymine (19.5 mmol, 2.4 g) and the crude diacetate from above (13.0 mmol) in acetonitrile (65 mL). The reaction was warmed until a clear solution formed after which it was cooled in an ice-bath and trimethylsilyl trifluoromethanesulfonate (TMSOTf, 19.5 mmol, 3.5 mL) was added. The reaction was warmed to room temperature and refluxed for 2 hours and cooled back to room temperature. The solvent was evaporated under reduced pressure on a rotary evaporator and the residue was diluted with EtOAc. The organic layer was washed with water, aqueous saturated $NaHCO_3$ (caution – effervescence), brine, dried (Na_2SO_4) and concentrated to provide the crude nucleoside which was used without any further purification. Methanolic ammonia (50 mL) was added to the nucleoside from above and the reaction was aged at room temperature for 12 h. The solvent was removed under reduced pressure on a rotary evaporator and the residue was purified by column chromatography (silica gel, eluting with 40–75% ethyl acetate in hexanes) to provide the 2'-deprotected nucleoside (4.16 g, 61% over 3 steps). ^{1}H NMR (300MHz, $CDCl_3$): δ 9.00 (br. s., 1H), 7.93 - 7.75 (m, 4H), 7.57 - 7.40 (m, 3H), 6.98 (s, 1H), 6.56 (td, *J*=1.5, 8.5 Hz, 1H), 5.54 - 5.39 (m, 1H), 4.92 - 4.80 (m, 2H), 4.76 (t, *J*=6.9 Hz, 1H), 4.48 (br. s., 1H), 4.27 (t, *J*=5.6 Hz, 1H), 3.34 (br. s., 1H), 2.07 - 2.01 (m, 1H), 1.89 (s, 3H), 1.26 (tt, *J*=1.5, 7.2 Hz, 1H). ^{13}C NMR (75MHz, $CDCl_3$): δ 163.7, 150.2, 138.0, 135.1, 133.9, 133.3, 133.2, 128.7, 128.0, 127.8, 127.2, 126.5, 126.4, 125.7, 111.2, 95.1, 95.0, 82.4, 80.4, 77.5, 77.2, 77.0, 76.6, 73.4, 72.8, 12.8; LRMS (ESI) calcd for $C_{22}H_{21}Br_2N_2O_5$ $[M + H]^+$ m/z = 552.98; found 552.9.

Sodium hexamethyldisilane (17.4 mL, 17.4 mmol) was added to a cold (-78 °C) solution of the nucleoside (7.9 mmol, 4.5 g) from above in THF (40 mL). The reaction was stirred for 30

minutes after which it was transferred to an ice bath. Iodomethane (87 mmol, 5.4 mL) was added to the reaction and the stirring was continued for another 20h at 0 °C. The reaction was quenched by adding saturated NH_4Cl solution (5 mL) and ~80% of the solvent was removed under reduced pressure on a rotary evaporator. The residue was dissolved in EtOAc and the organic layer was washed with water, brine, dried (Na_2SO_4) and concentrated. Purification by column chromatography (silica gel, eluting with 10–25% EtOAc in hexanes) provided nucleoside **S33** and the corresponding mono-bromo alkynyl nucleoside (3.2 g) as an unseparable mixture. ^{1}H NMR (300MHz, $CDCl_3$): δ 9.08 (s, 1H), 8.96 (s, 1H), 7.93 - 7.76 (m, 6H), 7.60 - 7.41 (m, 4H), 6.97 (d, *J*=0.9 Hz, 1H), 6.64 (td, *J*=1.7, 8.3 Hz, 1H), 6.15 - 6.04 (m, 1H), 5.69 - 5.54 (m, 1H), 5.37 - 5.22 (m, 1H), 4.92 - 4.77 (m, 4H), 4.24 (t, *J*=4.5 Hz, 1H), 4.17 - 4.08 (m, 1H), 4.07 - 4.00 (m, 1H), 4.00 - 3.95 (m, 1H), 3.54 (s, 1H), 3.51 - 3.44 (m, 4H), 2.07 - 2.02 (m, 1H), 1.92 (d, *J*=1.3 Hz, 1H), 1.89 (d, *J*=1.1 Hz, 3H), 1.71 - 1.64 (m, 1H), 1.26 (tt, *J*=1.6, 7.1 Hz, 1H). ^{13}C NMR (75MHz, $CDCl_3$): δ 163.8, 150.2, 149.9, 137.6, 135.1, 134.5, 134.2, 133.2, 133.2, 128.5, 128.5, 128.5, 128.0, 127.8, 126.9, 126.4, 126.3, 126.2, 125.7, 125.6, 111.4, 95.0, 92.5, 88.7, 82.5, 82.1, 80.5, 79.3, 77.5, 77.0, 76.6, 76.5, 72.9, 72.7, 58.7, 12.9, 12.5, 0.01; LRMS (ESI) calcd for $C_{23}H_{23}Br_2N_2O_5$ $[M + H]^+$ m/z = 567.00; found 566.9.

Compound S34. A solution of the mono- and di-bromo nucleoside mixture from above (3.2 g) in DMF (80 mL) was added *via* cannula to a solution of palladium acetate (1.2 mmol, 0.27 g) and 1,1'-Bis(diphenylphosphino)ferrocene (dppf, 2.4 mmol, 1.3 g) in DMF (32 mL).2-Methyloxirane (18 mL, 1.26 mL) and diethylphosphite (12 mmol, 1.5 mL) were added and the reaction was heated at 80 ^{o}C for 10 hours. The reaction was cooled to room temperature and diluted with EtOAc. The organic layer was washed with water, brine, dried (Na_2SO_4) and concentrated. Purification by column chromatography (silica gel, 25 – 95% ethyl acetate in

hexanes) provided **S34** (2.18 g, 67%). ^{1}H NMR (300MHz, $CDCl_3$): δ 8.78 (br. s., 1H), 7.93 - 7.77 (m, 4H), 7.58 - 7.43 (m, 3H), 7.31 - 7.19 (m, 2H), 6.11 (d, *J*=4.5 Hz, 1H), 4.95 - 4.81 (m, 4H), 4.32 - 4.08 (m, 7H), 4.02 (t, *J*=4.5 Hz, 1H), 3.48 (s, 4H), 1.94 (d, *J*=0.9 Hz, 3H), 1.36 (t, *J*=7.1 Hz, 6H); ^{13}C NMR (75MHz, $CDCl_3$): δ 163.4, 150.1, 134.9, 133.9, 133.2, 128.6, 128.0, 127.8, 127.0, 126.5, 126.4, 125.6, 111.8, 95.1, 94.4, 88.8, 82.2, 81.3, 80.2, 77.5, 77.2, 77.0, 76.6, 73.1, 71.6, 71.5, 63.7, 63.6, 58.7, 16.1, 16.0, 12.6, 0.0; ^{31}P NMR (121MHz, $CDCl_3$): δ -8.14 (s, 1P); LRMS (ESI) calcd for $C_{27}H_{32}N_2O_8P$ $[M + H]^+$ *m/z* = 543.19; found 543.1.

Compound S35. DDQ (8.0 mmol, 1.61 g) was added to a solution of **S34** (4.0 mmol, 2.18 g) in dichloromethane (40 mL) and water (2 mL). The reaction mixture was stirred at room temperature for 16 h after which the solvent was removed using a rotary evaporator and the residue was dissolved in EtOAc. The organic later was washed with sodium bisulfite, $NaHCO_3$ (caution – effervescence), brine and dried (Na_2SO_4). Each aqueous layer was further extracted with dichloromethane, dried (Na_2SO_4) and the combined organic layers were concentrated. Purification by column chromatography (silica gel, 10–40% acetone in dichloromethane) provided the 3'-deprotected nucleoside (1.44 g, 90%). ^{1}H NMR (300MHz, $CDCl_3$): δ 7.30 (d, *J*=0.9 Hz, 1H), 6.08 (d, *J*=4.0 Hz, 1H), 4.74 (dd, *J*=3.5, 4.6 Hz, 1H), 4.42 (q, *J*=5.1 Hz, 1H), 4.30 - 4.11 (m, 4H), 4.00 (t, *J*=4.3 Hz, 1H), 3.57 (s, 3H), 3.44 (br. s., 1H), 1.98 (s, 3H), 1.40 (t, *J*=7.1 Hz, 6H). ^{13}C NMR (75MHz, $CDCl_3$): δ 163.6, 150.2, 134.8, 112.0, 95.1, 94.5, 88.3, 83.0, 81.1, 77.4, 77.2, 77.1, 76.6, 74.3, 74.2, 73.8, 73.7, 63.8, 63.7, 58.8, 16.2, 16.1, 12.7, 0.0; ^{31}P NMR (121MHz, $CDCl_3$): δ -8.10 (s, 1P); LRMS (ESI) calcd for $C_{16}H_{24}N_2O_8P$ $[M + H]^+$ *m/z* = 403.15; found 403.1. It was dissolved in ahydrous DMF (13 mL) and imidazole (2.20 g, 32.31 mmol) and *tert*-butyldimethylchlorosilane (2.68 g, 17.82 mmol) were added. The reaction mixture was stirred at room temperature under argon atmosphere for 4 h. The reaction mixture was diluted

with EtOAc (200 mL) and washed with water (200 mL), aqueous saturated sodium bicarbonate (200 mL) and brine, dried (Na_2SO_4), filtered and concentrated. The residue obtained was purified by silica gel column chromatography and eluted with 85% EtOAc in hexane to yield **S35** (1.16 g, 63%). ^{1}H NMR (300MHz, $CDCl_3$): δ 8.32 (br. s., 1H), 7.29 (s, 1H), 6.04 (d, *J*=4.9 Hz, 1H), 4.66 (t, *J*=3.7 Hz, 1H), 4.39 (t, *J*=4.2 Hz, 1H), 4.21 (t, *J*=7.2 Hz, 4H), 3.90 (t, *J*=4.6 Hz, 1H), 3.46 (s, 3H), 1.99 (s, 3H), 1.40 (t, *J*=7.1 Hz, 6H), 0.93 (s, 9H), 0.15 (s, 6H); ^{31}P NMR (121MHz, $CDCl_3$): δ 33.12; LR MS (ESI) calcd for $C_{22}H_{38}N_2O_8PSi$ $[M + H]^+$ *m/z* = 517.6, found 517.2.

Compound S36. Compound **S35** (0.91 g, 2.04 mmol) was dissolved in methanol (8 mL) and Lindlar catalyst (0.44 g) was added. Air was replaced by applying vacuum and flushed the reaction with H_2 gas. This process was repeated for 3 times. The reaction mixture was stirred under H_2 gas for 5 h. Filtered the reaction mixture through a pad of celite and washed the celite pad thoroughly with methanol. The filtrate and the washings were combined together and concentrated under reduced pressure. The residue obtained was purified by silica gel column chromatography and eluted with 0-10% methanol in dichloromethane to yield **S36** (0.70 g, 94.5%). ^{1}H NMR (300MHz, $CDCl_3$): δ 8.90 (s, 1H), 7.15 (s, 1H), 6.68 - 6.31 (m, 1H), 5.89 (dd, *J*=13.4, 15.7 Hz, 1H), 5.61 (d, *J*=4.1 Hz, 1H), 5.30 (s, 1H), 4.21 - 4.03 (m, 6H), 3.46 (s, 3H), 1.94 (s, 3H), 1.33 (t, *J*=7.0 Hz, 6H), 0.92 (s, 9H), 0.13 (s, 3H), 0.12 (s, 3H); ^{31}P NMR (121MHz, $CDCl_3$): δ 14.18; LR MS (ESI) calcd for $C_{22}H_{40}N_2O_8PSi$ $[M + H]^+$ *m/z* = 519.6, found 519.2.

Compound 54. To a solution of compound **60** (34.7 g, 82.62 mmol) in anhydrous acetonitrile (180 mL) 1H-tetrazole (4.63 g, 66.1 mmol), followed by 1-methylimidazole (1.65 ml, 20.66 mmol) and 2-cynoethyl-*N*,*N*,*N*',*N*'-tetraisopropylphosphorodiamidite (32.8 ml, 103.28 mmol) were added. The reaction mixture was stirred at room temperature for 3 h and diluted with

EtOAc (400 mL) washed with aqueous saturated sodium bicarbonate solution (400 mL), brine (400 mL), dried (Na_2SO_4), filtered and evaporated under reduced pressure. The residue obtained was purified by silica gel column chromatography and eluted with 50% acetone in dichloromethane to yield **54** (40 g, 78%). ^{31}P NMR (121MHz, $CDCl_3$): δ 150.40, 150.06, 20.40, 19.68; HR MS (ESI) calcd for $C_{25}H_{41}N_4O_{10}P_2$ [M - H]$^-$ *m/z* = 619.2285, found 619.2286.

Compound 55. Compound **S36** (0.69 g, 1.34 mmol) was dissolved in THF (4 mL) and triethylamine trihydrofluoride (1.1 mL, 6.72 mmol) was added and stirred at room temperature for 6h. The solvent was removed under reduced pressure and the residue was purified by silica gel column chromatography and eluted with 5% methanol in dichloromethane to yield 3'-hydroxyderivative. ^{1}H NMR (300MHz, $CDCl_3$): δ 9.31 (s, 1H), 7.09 (s, 1H), 6.79 - 6.42 (m, 1H), 5.98 - 5.81 (m, 1H), 5.74 (d, *J*=2.4 Hz, 1H), 5.38 (t, *J*=7.7 Hz, 1H), 4.27 - 3.98 (m, 6H), 3.93 (dd, *J*=2.4, 5.4 Hz, 1H), 3.57 (s, 3H), 1.94 (s, 3H), 1.43 - 1.30 (m, 6H); ^{13}C NMR (75MHz, $CDCl_3$): δ 163.7, 150.0, 148.9, 136.2, 119.0, 111.1, 90.7, 82.5, 79.9, 74.7, 62.3, 62.2, 58.9, 16.3, 16.2, 12.5; ^{31}P NMR (121MHz, $CDCl_3$): δ 16.04; LR MS (ESI) calcd for $C_{16}H_{26}N_2O_8P$ [M + H]$^+$ *m/z* = 404.4, found 404.1. The 3'-hydroxy derivative (0.50 g, 1.24 mmol) was phosphitylated to yield compound **55** (0.63 g, 84%), acetonitrile (3 mL), 1H-tetrazole (0.069 g, 0.94 mmol), *N*-methylimidazole (0.025 mL, 0.31 mmol) and 2-cynoethyl-*N*,*N*,*N*',*N*'-tetraisopropylphosphordiamidite (0.59 mL, 1.86 mmol) following the same procedure used for the synthesis of compound **30b**. ^{31}P NMR (121MHz, $CDCl_3$): δ 150.15, 149.53, 14.19, 14.12; HR MS (ESI) calcd for $C_{25}H_{41}N_4O_9P_2$ [M - H]$^-$ *m/z* = 603.2339, found 603.2343.

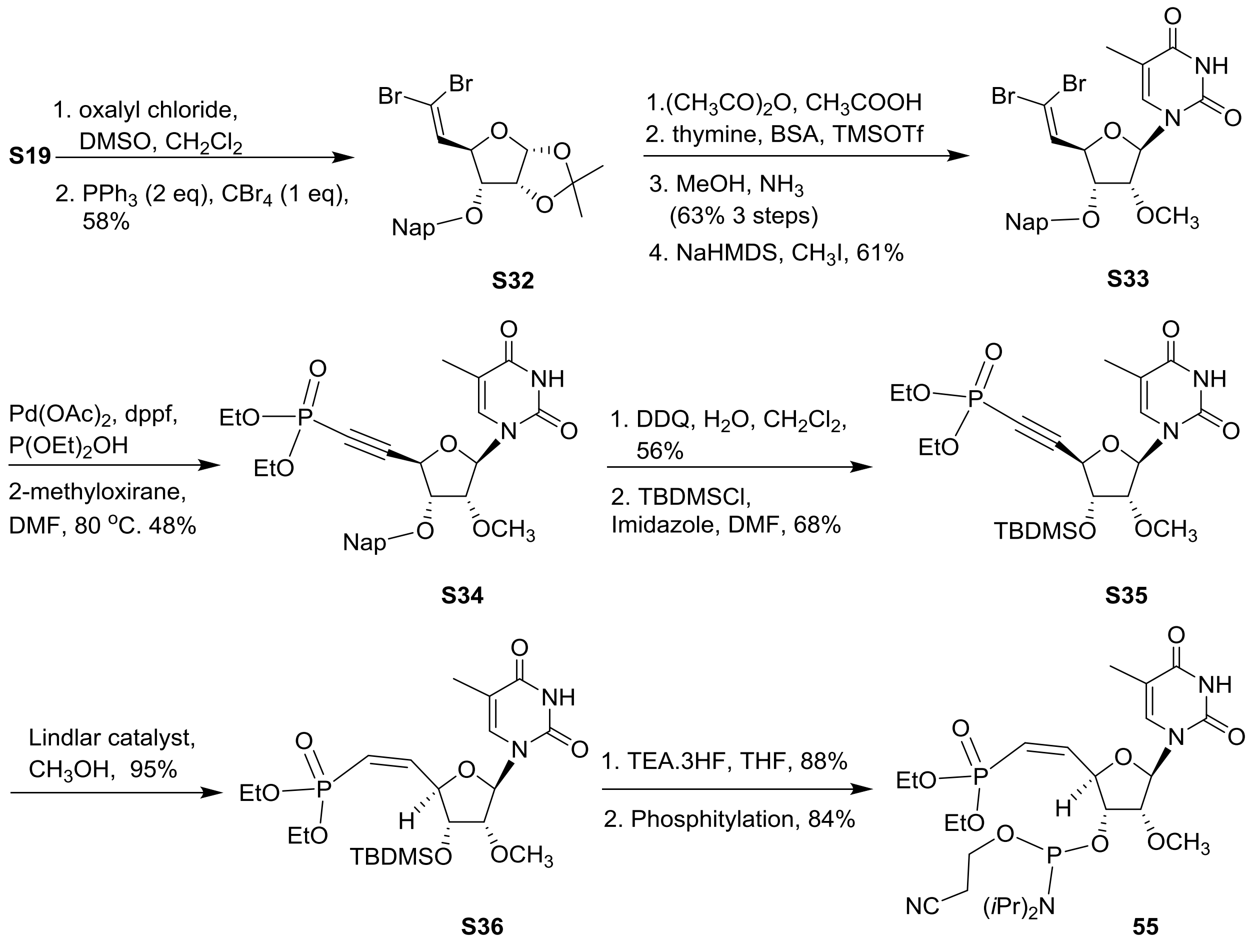

Scheme S9. Synthesis of compound **55**

Compound S37. A solution of partially pure **S26** (0.83 g) in methanolic ammonia (5 mL of a 7N solution) was aged at room temperature for 8 h after which the solvent was evaporated under reduced pressure. Purification by chromatography (silica gel, eluting with 20 to 75% acetone in dichloromethane) provided **S37** (0.38 g). ^{1}H NMR (300 MHz, $CDCl_3$): δ 9.1 (br s, 1H), 7.10 (s, 1H), 6.14 (td, *J*=8.1, 37.7 Hz, 1H), 5.80 (d, *J*=3.0 Hz, 1H), 4.88 (t, *J*=7.1 Hz, 1H), 4.34–3.96 (m, 7H), 3.88–3.69 (m, 2H), 3.67–3.48 (m, 2H), 3.40 (s, 3H), 1.94 (s, 3H), 1.48–1.31 (m, 6H). ^{13}C NMR (75 MHz, $CDCl_3$): δ 163.6, 156.9, 153.5, 135.8, 121.8, 121.9, 121.6, 111.3, 90.1, 81.7, 76.7, 73.8, 71.6, 70.5, 63.6, 58.9, 16.3, 12.6. ^{31}P NMR (121MHz, $CDCl_3$): δ 3.47 (d, *J*=98.0 Hz,

1P). ^{19}F NMR (282 MHz, $CDCl_3$): δ -123.55 (dd, *J*=37.9, 96.4 Hz, 1F). LR MS (ESI) calcd for $C_{18}H_{29}FN_2O_9P$ $[M + H]^+$ *m/z* = 467.4, found 467.1.

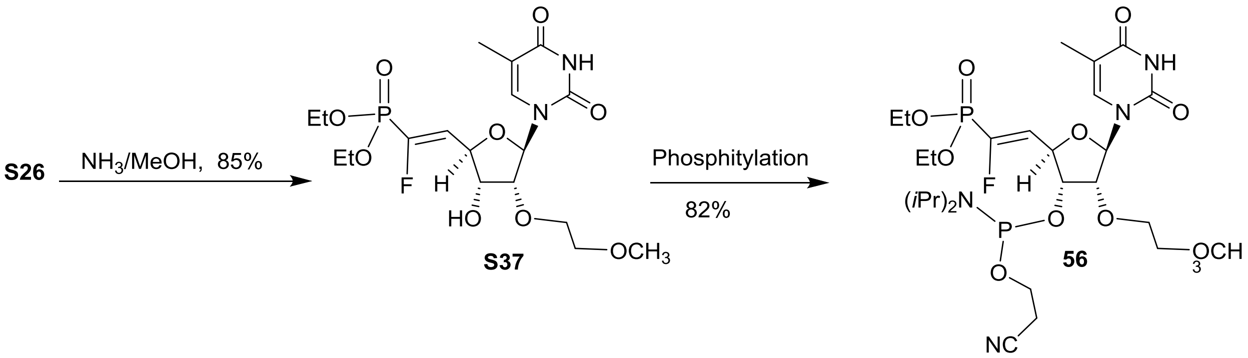

Scheme S10. Synthesis of compound **56**

Compound 56. 2-Cyanoethyl tetraisopropylphosphordiamidite (0.19 mL, 0.63 mmol) was added to a solution of **S37** (0.20 g, 0.42 mmol), 1*H*-tetrazole (24 mg, 0.34 mmol) and *N*-methylimidazole (1 drops) in DMF (2.1 mL). After stirring at room temperature for 4 h the reaction was diluted EtOAc and the organic layer was washed with brine, dried (Na_2SO_4) and concentrated. Purification by chromatography (silica gel, eluting with 30% acetone in dichloromethane) provided **56** (0.25 g, 89%) as a white solid. ^{31}P NMR (121MHz, $CDCl_3$): δ 150.66 (1P), 149.85 (1), 3.56 (d, *J*=98.7 Hz, 1P), 3.28 (d, *J*=98.7 Hz, 1P). ^{19}F NMR (282MHz, $CDCl_3$): δ -122.55 (dd, *J*=37.9, 97.5 Hz, 1F), -123.33 (dd, *J*=37.9, 97.5 Hz, 1F). LR MS (ESI) calcd for $C_{27}H_{44}FN_4O_{10}P_2$ $[M - H]^-$ *m/z* = 665.6, found 665.2.

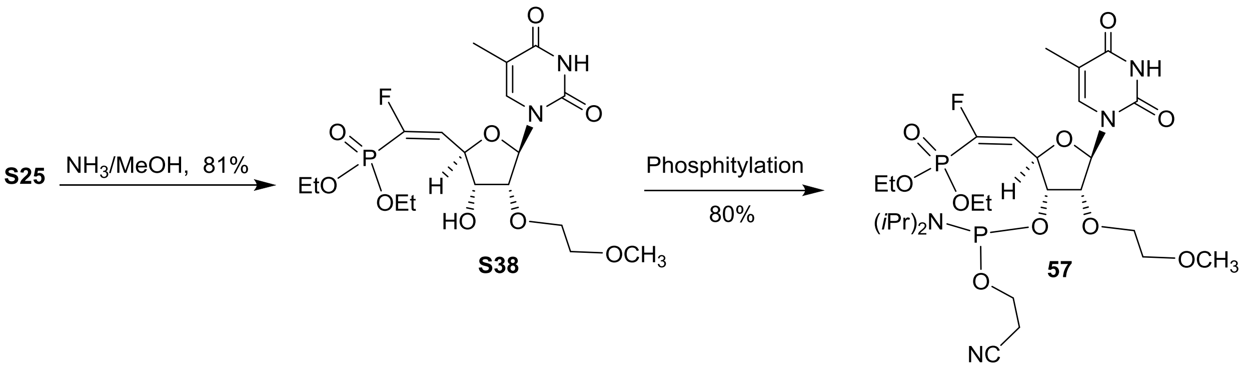

Scheme S11. Synthesis of compound **57**

Compound S38. A solution of partially pure **S25** (0.84 g) in methanolic ammonia (4 mL of a 7N solution) was aged at room temperature for 8 hours after which the solvent was evaporated under reduced pressure. Purification by chromatography (silica gel, eluting with 20 to 75% acetone in dichloromethane) provided **S38** (0.20 g) which was dried under high vacuum for 16 hours. ^{1}H NMR (300 MHz, $CDCl_3$): δ 7.10 (s, 1H), 6.20 (ddd, *J*=9.6, 26.0, 30.0 Hz, 1H), 5.66 (d, *J*=1.5 Hz, 1H), 5.41–5.23 (m, 1H), 4.34–3.77 (m, 7H), 3.68–3.50 (m, 2H), 3.38 (s, 3H), 1.94 (s, 3H), 1.48–1.31 (m, 6H). ^{13}C NMR (75 MHz, $CDCl_3$): δ 164.2, 153.2, 149.8, 136.7, 124.4, 111.0, 91.7, 81.5, 76.9, 76.8, 74.6, 71.8, 70.5, 63.6, 58.9, 16.2, 12.5. ^{31}P NMR (121 MHz, $CDCl_3$): δ 2.72 (d, *J*=100.5 Hz, 1P). ^{19}F NMR (282 MHz, $CDCl_3$): δ -114.54 (dd, *J*=26.4, 99.8 Hz, 1F). LR MS (ESI) calcd $[M + H]^+$ *m/z* = 467.2, found 467.1.

Compound 57. 2-Cyanoethyl tetraisopropylphosphorodiamidite (0.10 mL, 0.32 mmol) was added to a solution of **S38** (0.1 g, 0.21 mmol), 1*H*-tetrazole (12 mg, 0.17 mmol) and *N*-methylimidazole (1 drops) in DMF (1.0 mL). After stirring at room temperature for 4 h the reaction was diluted EtOAc and the organic layer was washed with brine, dried (Na_2SO_4) and concentrated. Purification by chromatography (silica gel, eluting with 30% acetone in dichloromethane) provided **57** (0.12 g, 85%) as a white solid. ^{31}P NMR (121 MHz, $CDCl_3$): δ

149.42 (s, 1P), 149.15 (s, 1P), 1.87 (d, *J*=100.5 Hz, 1P), 1.83 (d, *J*=100.5 Hz, 1P). ^{19}F NMR (282 MHz, $CDCl_3$): δ -112.11 (dd, *J*=26.4, 99.8 Hz, 1F), -112.80 (dd, *J*=26.4, 102.1 Hz, 1F); HR MS (ESI) calcd for $C_{27}H_{44}FN_4O_{10}P_2$ $[M - H]^-$ *m/z* = 665.3572, found 665.3572.

Compound 58. To a solution of compound **40** (105 g, 169.9 mmol) in DMF (600 ml) was added imidazole (116 g, 1669 mmol), followed by TBDPSCl (217 ml, 849.5 mmol). The reaction mixture was stirred at room temperature for 18 h and diluted with EtOAc (800 mL) and washed with water (3 x 1 L). The organic phase was washed aqueous saturated sodium bicarbonate (500 mL), brine (500 mL), dried (Na_2SO_4) and concentrated. The residue was purified by silica gel column chromatography and eluted first with 30% EtOAc in hexane to afford 3'-TBDPS analog of compound **40** (145 g, 100%). This was dissolved in CH_2Cl_2 (600 ml) was added TFA (30.2 ml, 391.1 mmol), followed by Et_3SiH (59.3 ml, 371.5 mmol). The reaction mixture was stirred for 1 h and solvent was removed under reduced pressure. Residue was redissolved in EtOAc (600 mL) and organic phase was with aqueous saturated sodium bicarbonate (500 mL), brine (500 mL), dried (Na_2SO_4), filtered and concentrated. The residue was purified by silica gel column chromatography and eluted first with 10-70% EtOAc in hexane to yield **58** (93.3 g, 97%). ^{1}H NMR (300MHz, DMSO-d_6): δ 11.32 (s, 1H), 7.76 - 7.57 (m, 5H), 7.54 - 7.33 (m, 6H), 5.98 (d, *J*=5.9 Hz, 1H), 5.12 (t, *J*=4.5 Hz, 1H), 4.32 (t, *J*=4.0 Hz, 1H), 3.91 (d, *J*=2.8 Hz, 1H), 3.77 (t, *J*=5.3 Hz, 1H), 3.54 - 3.38 (m, 2H), 3.37 - 3.17 (m, 4H), 3.14 (s, 3H), 1.74 (s, 3H), 1.05 (s, 9H); ^{13}C NMR (75MHz, $CDCl_3$): δ 164.1, 150.3, 138.1, 135.8, 135.7, 134.7, 133.2, 132.9, 130.1, 130.0, 127.8, 127.7, 127.6, 110.4, 90.8, 85.3, 80.9, 71.8, 70.5, 69.7, 61.0, 58.9, 26.8, 19.2, 12.2; LR MS (ESI) calcd for $C_{29}H_{39}N_2O_7Si$ $[M + H]^+$ *m/z* = 555.7, found 555.4.

Compound 59: To the solution of compound **58** (93.3g, 168.41mmol) in DMSO (320ml) was added DCC (52.03 g, 252.6 mmol), followed by PyTFA (32.5 g, 168.4 mmol), the reaction

mixture was stirred at room temperature for 1.5 h. In a separate round bottom flask tetramethyl methylenediphosphonate (71.05 ml, 336.82 mmol) was dissolved in THF (200 ml), cooled to 0 ^{o}C and added 1.0 M potassium-*tert*-butoxide in THF (320 ml, 320 mmol), stirred at 0 ^{o}C for 10 min, then warmed up to room temperature fo 30 min. The round bottom flask containing compound **58** was cooled to 0 ^{o}C. To this the solution of tetramethylbisphosphonate anion solution from above was added *via* cannula. The reaction mixture was stirred at 0 ^{o}C for 1 h and then allowed to warmed up to room temperature in 1 h. To the reaction mixture water (500 mL) was added and extracted with EtOAc (2 x 400 mL). The organic phase dried (Na_2SO_4), filtered and concentrated. The residue was purified by silica gel column chromatography and eluted with EtOAc to yield **59** (74 g, 67.9%). ^{1}H NMR (300MHz, DMSO-d$_6$): δ 11.39 (s, 1H), 7.75 - 7.58 (m, 4H), 7.53 - 7.37 (m, 7H), 6.69 - 6.46 (m, 1H), 5.96 - 5.79 (m, 2H), 4.50 - 4.39 (m, 1H), 4.32 (t, *J*=5.2 Hz, 1H), 3.77 (t, *J*=4.9 Hz, 1H), 3.63 - 3.53 (m, 6H), 3.46 - 3.36 (m, 1H), 3.32 - 3.25 (m, 2H), 3.20 (dd, *J*=4.8, 10.4 Hz, 1H), 3.15 (s, 3H), 1.75 (s, 3H), 1.05 (s, 9H); ^{13}C NMR (75MHz, $CDCl_3$): δ 163.8, 150.0, 148.2, 148.1, 135.8, 135.7, 135.3, 132.5, 130.1, 130.0, 119.4, 116.9, 110.9, 88.7, 82.7, 82.4, 80.8, 74.4, 71.7, 69.6, 58.9, 52.4, 52.4, 52.3, 26.7, 19.1, 12.4; ^{31}P NMR (121MHz, $CDCl_3$): δ 19.60; LR MS (ESI) calcd for $C_{32}H_{44}N_2O_9PSi$ $[M + H]^+$ m/z = 659.8, found 659.4.

Compound 60. Compound **59** (72 g, 114.19 mmol) was dissolved in THF (260 ml) and triethylaminetrihydrofluoride (93 ml, 571 mmol) was added. The reaction mixure was stirred at room temperature for 18 h. The reaction mixture was concentrated under reduced pressure and the residue obtained was purified by silica gel column chromatography and eluted with 50% acetone in CH_2Cl_2 to afford compound **60** (38.7 g, 80.7%). ^{1}H NMR (300MHz, DMSO-d$_6$): δ 11.42 (br. s., 1H), 7.47 (d, *J*=1.0 Hz, 1H), 6.88 - 6.67 (m, 1H), 6.16 - 5.90 (m, 1H), 5.83 (d,

J=4.1 Hz, 1H), 5.41 (d, *J*=5.8 Hz, 1H), 4.45 - 4.26 (m, 1H), 4.21 - 4.06 (m, 2H), 3.80 - 3.57 (m, 9H), 3.22 (s, 3H), 1.79 (s, 3H); ^{13}C NMR (75MHz, $CDCl_3$): δ 163.9, 150.2, 148.5, 135.5, 116.0, 111.2, 89.5, 82.9, 81.5, 72.9, 71.6, 70.4, 58.8, 52.6, 52.5, 12.5; ^{31}P NMR (121MHz, $CDCl_3$): δ 19.90; LR MS (ESI) calcd for $C_{16}H_{26}N_2O_9P$ $[M + H]^+$ *m*/*z* = 421.4, found 421.1.

Compound S40. To a suspension of palmitic acid (1.93 g, 7.52 mmol) in DMF (25 ml) *O*-(Benzotriazol-1-yl)-*N,N,N',N'*-tetramethyluronium tetrafluoroborate (TBTU, 2.66 g, 8.27 mmol) and *N*,*N*-diisopropylethylamine (5.24 ml, 30 mmol) were added. After stirring for 10 min at room temperature a solution of 2'-*O*-hexylamino-5'-*O*-(4,4'-dimethoxy)tritylthymidine[6] S39 in DMF (50 mL) was added. The reaction mixture was stirred at room temperature for 3 h under argon atmosphere. EtOAc (200 mL) was added and the organic phase was washed with saturated aqueous $NaHCO_3$ solution (200 mL), brine (200 mL), dried (Na_2SO_4), filtered and concentrated under reduced pressure. The residue was purified by silica gel column chromatography and eluted with 70% EtOAc in hexane to yield S40 (8.0 g, 100%). ^{1}H NMR (300MHz, $CDCl_3$): δ 8.90 - 8.75 (m, 1H), 7.66 (d, *J*=1.2 Hz, 1H), 7.35 - 7.20 (m, 9H), 6.84 (d, *J*=8.8 Hz, 4H), 5.99 (d, *J*=3.1 Hz, 1H), 5.74 - 5.58 (m, 1H), 4.53 - 4.38 (m, 1H), 4.18 - 4.07 (m, 1H), 4.05 - 3.97 (m, 1H), 3.91 - 3.82 (m, 1H), 3.90-3.82 (m, 1H), 3.79 (s, 6H), 3.73 - 3.61 (m, 1H), 3.61 - 3.50 (m, 1H), 3.50 - 3.37 (m, 1H), 3.24 (d, *J*=6.3 Hz, 2H), 2.14 (d, *J*=7.9 Hz, 2H), 1.82 (s, 3H), 1.62 (d, *J*=6.1 Hz, 4H), 1.51 - 1.43 (m, 2H), 1.41 - 1.14 (m, 28H), 0.98 - 0.78 (t, *J*=6 Hz, 3H); ^{13}C NMR (75MHz, $CDCl_3$): δ 173.3, 163.6, 158.7, 150.1, 144.3, 139.4, 135.4, 135.2, 130.1, 129.1, 128.2, 128.0, 127.8, 127.7, 127.2, 113.3, 111.1, 87.3, 86.9, 83.6, 82.1, 70.9, 69.1, 62.1, 55.2, 39.2, 36.9, 31.9, 29.7, 29.6, 29.5, 29.4, 29.3, 26.5, 26.3, 25.8, 25.5, 22.7, 14.1, 11.7; LR MS (ESI) calcd for $C_{53}H_{74}N_3O_9$ $[M -H]^-$ *m*/*z* = 897.2, found 896.8.

Compound S41. Compound **S40** (6.8 g, 7.57 mmol) dissolved in anhydrous DMF (20 ml) and imidazole (5.15 g, 75.7 mmol) and *tert*-butyldimethylchlorosilane (9.7 ml, 37.86 mmol) were added. The reaction mixture was stirred at room temperature for 6 h under argon atmosphere. EtOAc (200 mL) was added and organic phase thus obtained was washed with water (200 mL), brine (200 mL), dried (Na_2SO_4), filtered and concentrated under reduced pressure. The residue was purified by silica gel column chromatography and eluted with 50% EtOAc in hexane to yield **S41** (6.36 g, 74%). ^{1}H NMR (300MHz, $CDCl_3$): δ 8.61 (s, 1H), 7.66 (s, 1H), 7.45 - 7.16 (m, 19H), 6.81 - 6.75 (m, 4H), 6.09 (d, *J*=4.5 Hz, 1H), 5.63 - 5.55 (m, 1H), 4.38 - 4.29 (m, 1H), 4.22 - 4.09 (m, 1H), 3.78 (s, 6H) 3.81 - 3.72 (m, 1H), 3.70 - 3.60 (m, 1H), 3.58-3.34 (m, 2H), 3.28 - 3.11 (m, 2H), 2.99-2.90 (m, 1H), 2.16 (t, *J*=7.6 Hz, 2H), 1.68-1.55 (m, 2H), 1.55 - 1.38 (m, 4H), 1.29 (s, 3H), 1.30 - 1.18 (m, 28H), 1.00 (s, 9H), 0.93 - 0.81 (t, *J*=6 Hz, 3H); ^{13}C NMR (75MHz, $CDCl_3$): δ 173.2, 163.6, 158.7, 150.1, 144.2, 135.9, 135.7, 135.5, 135.4, 135.2, 133.1, 130.1, 130.0, 128.2, 128.0, 127.9, 127.7, 127.5, 127.1, 113.2, 110.9, 86.9, 83.9, 81.7, 71.4, 70.2, 62.6, 55.2, 39.4, 36.9, 31.9, 29.7, 29.6, 29.5, 29.4, 29.3, 26.8, 26.7, 25.8, 25.7, 22.7, 19.3, 14.1, 11.6; LR MS (ESI) calcd for $C_{69}H_{92}N_3O_9Si$ $[M - H]^-$ *m/z* = 1135.6, found 1135.2.

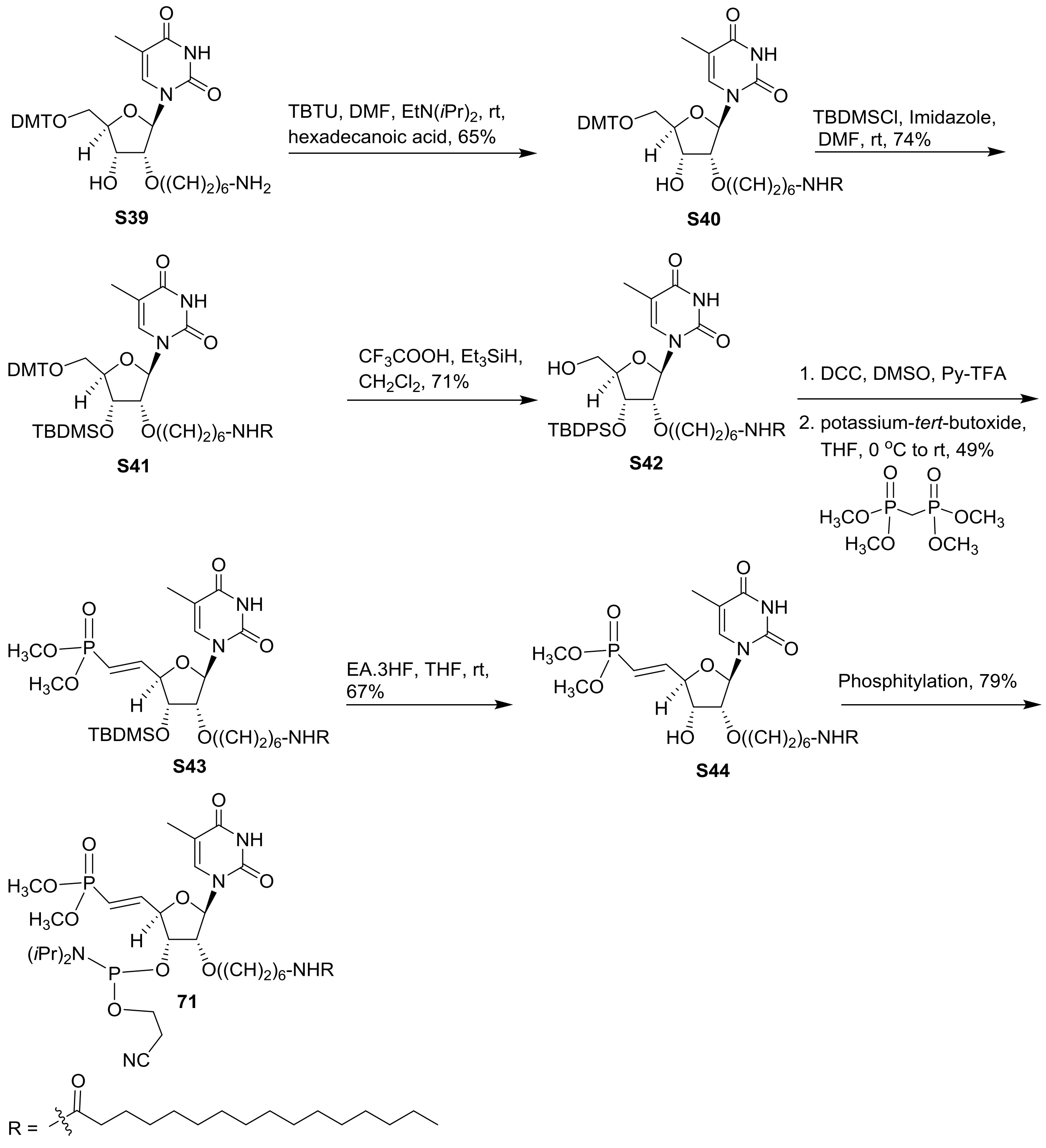

Scheme S12. Synthesis of compound **71**

Compound S42. Trifluoroacetic acid (0.45 ml, 5.80 mmol) was added to a solution of compound **S41** (3.3 g, 2.90 mmol) in CH_2Cl_2 (10 mL) followed by triethylsilane (0.88 ml, 5.51 mmol). After stirring at room temperature for 1 h solvent was removed under reduced pressure. The residue was re-dissolved in methanol (20 mL) and 0.3 M aqueous HCl (5 mL) was added and stirred at room temperature for 30 min. The solvent was removed under reduced pressure and residue was dissolved in EtOAc (200 mL) and washed with water (200 mL), brine (200 mL), dried (Na_2SO_4), filtered and concentrated. The residue obtained was purified by silica gel column chromatography and eluted with EtOAc to yield **S42** (2.4 g, 99.2%). ^{1}H NMR (300MHz, DMSO-d_6): δ 11.35 (s, 1H), 7.75 - 7.56 (m, 6H), 7.50 - 7.35 (m, 5H), 5.96 (d, *J*=5.9 Hz, 1H), 5.15 (m, 1H), 4.31 (m., 1H), 3.90 (m, 1H), 3.66 (m, 1H), 3.58 - 3.46 (m, 1H), 3.29 - 3.18 (m, 2H), 3.18 - 3.06 (m, 1H), 2.97 (m, 2H), 2.02 (t, *J*=7.4 Hz, 2H), 1.73 (s, 3H), 1.46 (m, 2H), 1.37 - 1.15 (m, 32H), 1.04 (s, 9H), 0.91 - 0.74 (t, *J*=6 Hz, 3H); ^{13}C NMR (75MHz, $CDCl_3$): δ 173.3, 164.0, 150.3, 137.7, 135.8, 135.7, 133.3, 133.0, 127.8, 127.6, 110.5, 90.1, 85.3, 80.7, 70.6, 70.4, 61.0, 39.3, 36.8, 31.9, 29.6, 29.5, 29.4, 29.3, 29.3, 26.8, 26.5, 25.8, 25.5, 22.6, 19.2, 14.1 12.3; LR MS (ESI) calcd for $C_{48}H_{75}N_3O_7Si$ $[M - H]^-$ *m/z* = 835.2; found 834.8.

Compound S43. Compound **S43** (2.78 g g, 46%) was synthesized from **S42** (5.2o g, 6.23 mmol), DMSO (10 mL) *N,N'*-dicyclohexylcarbodiimide (2.57 g, 12.46 mmol) and Py-TFA (1.80 g, 9.34 mmol), tetramethyl methylenediphosphonate (2.63 mL, 12.46 mmol) THF (150 ml), 1.0 M potassium-*tert*-butoxide in THF (12.50 mL, 12.46 mmol) using the procedure used for the synthesis of compound **59**. ^{1}H NMR (300MHz, $CDCl_3$): δ 7.77 - 7.59 (m, 4H), 7.51 - 7.32 (m, 6H), 6.80 (s, 1H), 6.73-6.56 (m, 1H), 5.96 (d, *J*=3.7 Hz, 1H), 5.93-5.75 (m, 1H), 5.66 - 5.50 (m, 1H), 4.69 - 4.53 (m, 1H), 3.92 (m, 1H), 3.82-3.66 (m, 6H), 3.37 (d, *J*=4.5 Hz, 2H), 3.29 - 3.09 (m, 2H), 2.16 (t, *J*=7.7 Hz, 2H), 1.85 (s, 3H), 1.62 (br. s., 2H), 1.56 - 1.40 (m, 2H), 1.36 - 1.20

(m, 32H), 1.10 (s, 9H), 0.91 - 0.82 (m, 3H); ^{13}C NMR (75MHz, $CDCl_3$): δ 173.2, 163.5, 149.9, 148.2, 148.1, 135.8, 135.0, 132.6, 130.2, 130.1, 127.9, 127.7, 119.5, 117.0, 111.3, 88.5, 82.9, 82.6, 74.5, 70.4, 52.5, 52.4, 39.4, 36.9, 31.9, 29.7, 29.6, 29.5, 29.4, 29.3, 26.8, 26.59, 25.8, 25.6, 19.2, 14.1, 12.5; ^{31}P NMR (121MHz, $CDCl_3$): δ 19.53; LR MS (ESI) calcd for $C_{51}H_{81}N_3O_9PSi$ $[M + H]^+$ m/z = 939.3; found 938.9.

Compound S44. To a solution of compound **S43** (2.78 g, 2.91 mmol) in THF (7.0 ml) triethylamine trihydrofluoride (2.37 ml, 14.55 mmol) was added and reaction mixture thus obtained was stirred at room temperature for 18 h. The reaction mixture was diluted with EtOAc (200 mL) and washed with water (200 mL), brine (200 mL), dried (Na_2SO_4), filtered and concentrated. The residue obtained was purified by silica gel column chromatography and eluted with EtOAc to yield **S44** (1.4 g, 67.3%). ^{1}H NMR (300MHz, $CDCl_3$): δ 8.99 (br. s., 1H), 7.06 (d, *J*=1.2 Hz, 1H), 6.98-6.88 (m, 1H), 6.19 - 5.97 (m, 1H), 5.92 (d, *J*=2.3 Hz, 1H), 5.66 - 5.50 (m, 1H), 4.54 - 4.41 (m, 1H), 4.05-3.95 (m, 1H), 3.92-3.87 (m, 1H), 3.83 - 3.75 (m, 7H), 3.66-3.54 (m, 1H), 3.30 - 3.22 (m, 2H), 2.21 - 2.13 (m, 2H), 1.78 (s, 3H), 1.61 (d, *J*=6.1 Hz, 4H), 1.53 - 1.46 (m, 2H), 1.46 - 1.22 (m, 30H), 0.95 - 0.81 (m, 3H); ^{13}C NMR (75MHz, $CDCl_3$): δ 173.3, 163.7, 150.1, 148.3, 148.2, 135.0, 118.7, 116.2, 111.5, 89.0, 82.9, 82.6, 81.2, 72.9, 72.9, 71.1, 52.6, 52.5, 39.1, 36.8, 31.8, 29.6, 29.5, 29.4, 29.3, 26.3, 25.8, 25.3, 22.6, 14.0, 12.6; ^{31}P NMR (121MHz, $CDCl_3$): δ 19.90; HR MS (ESI) calcd for $C_{35}H_{63}N_3O_9P$ [M + H]+ m/z = 700.4425; found 700.4427.

Compound 71. The compound **71** (1.2 g, 67%) was synthesized from compound **S44** (1.4 g, 1.96 mmol), DMF (5 ml), 1H-tetrazole (0.11 g, 1.56 mmol), *N*-methylimidazole (0.040 mL , 0.49 mmol) and 2-cynoethyl-*N*,*N*,*N*',*N*'-tetraisopropylphosphordiamidite (0.93 mL, 2.93 mmol)

following the same procedure used for the synthesis of compound **30b**. ^{31}P NMR (121MHz, $CDCl_3$): δ 150.39, 150.04, 20.38, 19.65; LR MS (ESI) calcd for $C_{44}H_{78}N_5O_{10}P_2$ [M - H]- *m/z* = 899.1; found 898.7.

Compound S46. Compound[6] **S46** (9.02 g, 66%) was synthesized from palmitic acid (3.97 g, 15.5 mmol), DMF (10.8 ml), TBTU (5.97 g, 18.60 mmol), *N*,*N*-diisopropylethylamine (10.80 ml, 62.00 mmol) and 2'-*O*-hexylamino-5'-*O*-(4,4'-dimethoxy)trityluridine (Reference) **S45** (10.00 g, 15.5 mmol in DMF (150 mL) using the same procedure used for the synthesis of compound **S40** . ^{1}H NMR (300MHz, $CDCl_3$): δ 8.03 (d, *J*=8.2 Hz, 1H), 7.52 - 7.18 (m, 10H), 6.93 - 6.76 (m, 4H), 5.95 (d, *J*=1.4 Hz, 1H), 5.64 (s, 1H), 5.27 (d, *J*=8.1 Hz, 1H), 4.46 (d, *J*=5.5 Hz, 1H), 4.03 (d, *J*=7.6 Hz, 1H), 3.96 - 3.86 (m, 2H), 3.80 (s, 7H), 3.65 (d, *J*=9.5 Hz, 1H), 3.58 - 3.48 (m, 2H), 2.85 (d, *J*=8.7 Hz, 1H), 2.14 (t, *J*=7.6 Hz, 2H), 1.75 - 1.56 (m, 4H), 1.56 - 1.42 (m, 3H), 1.42 - 1.12 (m, 31H), 0.98 - 0.78 (m, 3H); LR MS (ESI) calcd for $C_{52}H_{72}N_3O_9$ $[M - H]^-$ *m/z* = 883.2; found 882.8.

Compound 72. Compound **72** (7.3 g, 90%) was synthesized from compound **S46** (6.60 g,7.46 mmol), DMF (25 ml), 1H-tetrazole (0.42 g, 5.97 mmol), *N*-methylimidazole (0.15 mL, 1.88 mmol) and 2-cynoethyl-*N*,*N*,*N*',*N*'-tetraisopropylphosphordiamidite (2.96 mL, 9.33 mmol) following the same procedure used for the synthesis of compound **30b**. ^{31}P NMR (121MHz, $CDCl_3$): δ 150.36, 150.20; HR MS (ESI) calcd for $C_{61}H_{89}N_5O_{10}P$ $[M - H]^-$ *m/z* = 1082.6317; found 1083.6320.

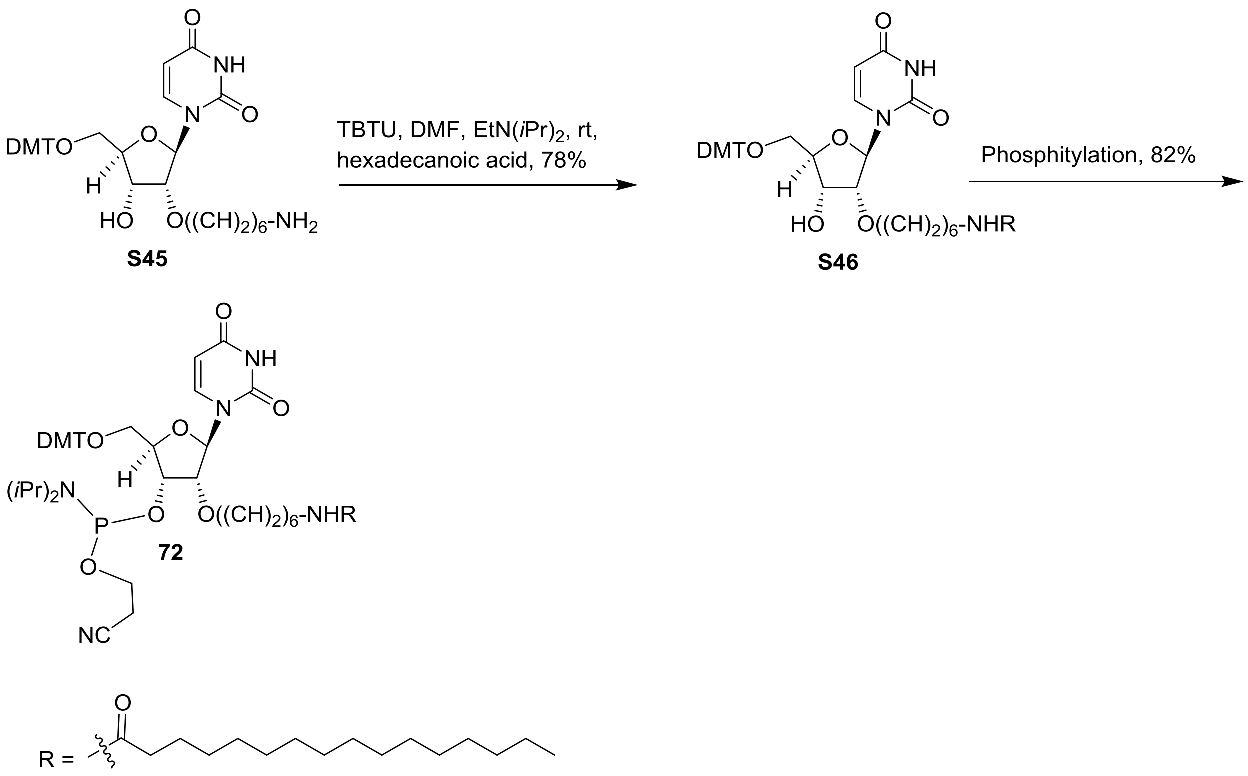

Scheme S13. Synthesis of compound **72**

REFERENCES

(1) Egli, M., Minasov, G.; Tereshko, V.; Pallan, P. S.; Teplova, M.; Inamati, G.; Lesnik, E.; Owens, S., Ross, B.; Prakash, T. P.; Manoharan, M. *Biochemistry* **2005,** *44,* 9045.

(2) Prakash, T. P.; Barber-Peoc'h, I.; Bhat, B.; Vasquez, G.; Ross, B. S.; Cook, P. D. *J. Org. Chem*. **1999**, *64*, 6468.

(3) Mesmaeker, A. D.; Lebreton, J.; Jouanno, C.; Fritsch, V.; wolf, R. M.; Wendeborn, S. *Synlett*, **1997**, 1287.
